# Supplementary material for: Recovery of homogeneous photocatalysts by covalent organic framework membranes
Source: Nat Commun. 2023 May 11;14:2726. doi: 10.1038/s41467-023-38424-6 (PMC10175538; doi:10.1038/s41467-023-38424-6)
Supplement: Supplementary file 1 — Supplementary Information [file 41467_2023_38424_MOESM1_ESM.pdf]

Supplementary Information

Recovery of Homogeneous Photocatalysts by Covalent Organic Framework Membranes

Hao Yang[1†], Jinhui Xu[2†], Hui Cao[2], Jie Wu[2*], Dan Zhao[1*]

[1] Department of Chemical and Biomolecular Engineering, National University of Singapore, 4 Engineering Drive 4, 117585 Singapore

[2] Department of Chemistry, National University of Singapore, 3 Science Drive 3, 117543 Singapore

† These authors contributed equally to this work.

* Corresponding author. Email: chmjie@nus.edu.sg (J.W.); chezhao@nus.edu.sg (D.Z.)

Supplementary Methods

1. Materials and Methods

1.1 Chemicals and materials

Polyacrylonitrile (PAN) substrate (molecular weight cut-off of 100,000 Da) was purchased from Shandong Lanjing Co Ltd. Calcium nitrate (>99%) was purchased from Merck Pte Ltd. Ethanol (EtOH, >99.8%), *N,N*-dimethylformamide (DMF, >99.5%), *N*,*N*-dimethylacetamide (DMA, >99.5%), dichloromethane (DCM, >99.5%) methanol (MeOH, >99.5%), tetrahydrofuran (THF, >99.5%), acetonitrile (MeCN, >99.5%), dimethyl sulfoxide (DMSO, >99.5%), Ethyl acetate (EtOAc, >99.5%)and 1,4-dioxane (>99.5%) were obtained from Avantor Performance Materials Inc. Polyethylene oxide (PEO, 98%) was purchased from Merck Pte Ltd. *p*-Phenylenediamine (PDA, 97%) was purchased from Alfa Aesar. 1,3,5-Tris(4-aminophenyl)benzene (TAPB, 98%), mesitylene (>99.5%), 3,3-dihydroxybenzidine (DHBD, 98%), pyrrolidine (>98%), Ethyl 2-mercaptopropionate (>97%), and 1-hexene (>99.5%) were purchased from Tee Hai Chem Pte Ltd. Hydrazine hydrate (HZ, >98%) was purchased from Merck Pte Ltd. *N*-acetyl-L-phenylalanine (Ac-Phe-OH, >95%) was purchased from BLD Pharmatech Ltd. 1,3,5-Triformylphloroglucinol (Tp, 98%) was purchased from Yanshen Technology Co Ltd. 4-Bromobenzotrifluoride (>99%), 4-methylquinoline (>99%), 1,4-diazabicyclo[2.2.2]octane (DABCO, >99%), 4-bromobenzenesulfonyl chloride (>98%), tert-butyl piperazine-1-carboxylate (>99%), and hexafluoro-2-propanol (HFIP, >99.5%) were purchased from Oakwood. Quinoline (>97%), fasudil monohydrochloride (>98%), *p*-toluenesulfonic acid monohydrate ($TsOH \cdot H_2O$, >98.5%), *N*-fluorobenzenesulfonimide (NFSI, >97%), nickel(II) bromide 2-methoxyethyl ether complex ($NiBr_2 \cdot$glyme, >97%), $[(Ru(bpy)_3](PF_6)_2$ (denoted as **$[Ru](PF_6)_2$**, >97%), $[Ir\{dF(CF_3)ppy\}_2dtbbpy]PF_6$ (denoted as **$[Ir\text{-}1]PF_6$**, >99%), $[Ir(ppy)_2dtbbpy]PF_6$ (denoted as **$[Ir\text{-}2]PF_6$**, >99%), $[Ir\{dF(CF_3)ppy\}_2bpy]PF_6$ (denoted as **$[Ir\text{-}3]PF_6$**, >99%), sodium decatungstate (denoted as **NaDT**, >97%), and (*R*)-3,3'-Bis(2,4,6-triisopropylphenyl)-1,1'-bi-2-naphthol cyclic monophosphate (denoted as **(*R*)-TRIP**, >95%) were purchased from Sigma-Aldrich Pte Ltd. All the purchased materials were directly used without further purification. Tert-butyl-4-((4-bromophenyl)sulfonyl)piperazine-1-carboxylate[1], adamantane trifluoroborate potassium salt[2], $Ni(bpy)Br_2$[2], 1,3-dioxoisoindolin-2-yl acetyl-*L*-phenylalaninate[3], *N*-phenylbenzothioamide[4], 1-(2-vinylphenyl)-propenone[5], and $Co^{III}(dmgH)_2(4\text{-}NMe_2Py)Cl$[4] were prepared according to the reported procedures.

Preparation of tertbutyl-4-((4-bromophenyl)sulfonyl)piperazine-1-carboxylate

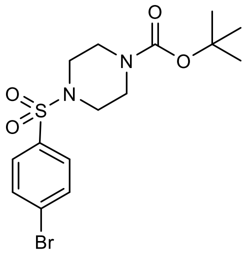

At 0°C, *N*-Boc-piperazine (6g, 36 mmol, 5 eq) was added to a solution of 4-bromobenzene sulfonyl chloride (2g, 7.8 mmol, 1 eq) in 10 mL THF. The reaction mixture was stirred at room temperature overnight. Water was added and the solution was

extracted with DCM, washed with saturated aqueous $NaHCO_3$, and dried over Na_2SO_4. The solvent was removed to afford the desired compound. **^{1}H NMR** (400 MHz, $CDCl_3$) δ 7.68 (d, *J* = 8.6 Hz, 2H), 7.60 (d, *J* = 8.7 Hz, 2H), 3.54-3.48 (m, 4H), 2.97 (t, *J* = 5.0 Hz, 4H), 1.41 (s, 9H). **^{13}C NMR** (125 MHz, $CDCl_3$) δ 154.11, 134.65, 132.57, 129.26, 128.23, 80.50, 45.89, 28.34.

Preparation of adamantane trifluoroborate potassium salt

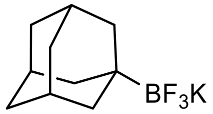

In a round bottom flask, a solution of 1-adamantane pinacolboronate ester (2.62 g, 10 mmol, 1 equiv.) in MeOH was cooled to 0 °C. After 5 minutes, an aqueous solution of KHF_2 (3.5 equiv, 4.5 M) was slowly added dropwise. The reaction mixture was then stirred at room temperature overnight. The solvent was removed under vacuum by rotary evaporation, and the resulting crude solid was dissolved in boiling acetone and filtered through a glass funnel to remove any inorganic salts. The filtrate was concentrated via rotary evaporation, and the resulting crude solid was washed successively with a 1:1 mixture of pentane/DCM and DCM to yield the pure potassium salt of adamantane trifluoroborate.**^{1}H NMR** (400 MHz, DMSO-d_6) δ 1.70 (br, 3H), 1.62 (m, 6H), 1.45 (m, 6H). **^{19}F NMR** (377 MHz, DMSO-d_6) δ -152.95. **^{11}B NMR** (128 MHz, DMSO-d_6) δ 4.38. **^{13}C NMR** (125 MHz, DMSO-d_6) δ 38.63, 38.34, 28.07.

Preparation of Ni(bpy)Br$_2$

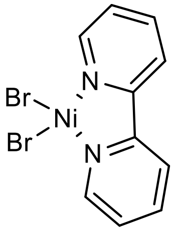

To a flask, $NiBr_2$·glyme (0.616 g, 2.0 mmol, 1.0 equiv.) and 2,2'-bipyridine (0.328 g, 2.1 mmol, 1.05 equiv.) were added, evacuated, and purged with argon three times. Absolute EtOH (8 mL) was then added, and the mixture was heated to vigorous reflux for 24 hours. After cooling the resulting suspension to room temperature, it was filtered through a medium porosity fritted glass funnel and washed with Et_2O (3×15 mL). The resulting dull yellow/green powder was dried under a high vacuum. **HRMS-ESI**: calcd for $C_{10}H_8BrN_2Ni^+$ ($[M-Br]^+$) m/z 292.9219, found 292.9220.

Preparation of 1,3-dioxoisoindolin-2-yl acetyl-L-phenylalaninate

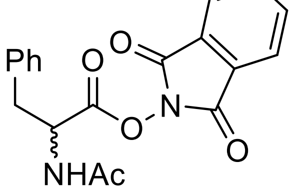

In a round bottom flask, *N*-Acetyl-*L*-phenylalanine (10 mmol, 1.0 equiv.), *N*-hydroxyphthalimide (NHPI, 1.1 equiv.), N,N'-dicyclohexylcarbodiimide (DCC, 1.2 equiv.) and 4-Dimethylaminopyridine (0.1 equiv.), and DCM (0.2 M) were added at room temperature. After completion (as indicated by thin layer chromatography), the solid mixture was filtered and washed with DCM. The filtrate was then concentrated in vacuo, and the desired product was obtained by triturating the crude mixture with EtOH, followed by isolation through vacuum filtration. **^{1}H NMR** (400 MHz, $CDCl_3$) δ 7.91 – 7.89 (m, 2H), 7.82-7.80 (m, 2H), 7.40 – 7.27 (m, 5H), 5.88 (d, *J* = 8.3 Hz, 1H), 5.36 (dt, *J* = 8.1, 5.8 Hz, 1H), 3.34 (qd, *J* = 14.2, 5.8 Hz, 2H), 1.99 (s, 3H). **^{13}C NMR** (125 MHz, $CDCl_3$) δ 169.89, 168.60, 161.56, 135.05, 134.65, 129.84, 128.90, 128.65, 127.63, 124.24, 51.27, 37.88, 23.05.

Preparation of N-phenylbenzothioamide

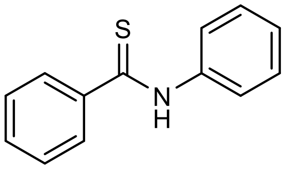

A mixture of the *N*-phenylbenzamide (1.97 g, 10 mmol, 1 equiv.) and Lawesson's reagent (0.6 equiv.) in dry toluene (80 mL) was heated at reflux under argon atmosphere for 2 h. The mixture was then concentrated. The product was purified by column chromatography (EtOAc/hexanes 1:4) and recrystallized from hexane/EtOAc. **^{1}H NMR** (400 MHz, $CDCl_3$) δ 9.03 (s, 1H), 7.85 (d, *J* = 7.5 Hz, 2H), 7.78 (d, *J* = 7.9 Hz, 2H), 7.54 – 7.37 (m, 5H), 7.30 (t, *J* = 7.5 Hz, 1H). **^{13}C NMR** (125 MHz, $CDCl_3$) δ 198.55, 143.15, 139.08, 131.36, 129.11, 128.70, 127.08, 126.81, 123.87.

Preparation of 1-(2-vinylphenyl)-propenone

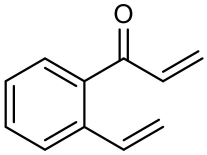

50 mL solution of DCM and 1-(2-vinylphenyl)propenol (1.60 g, 10 mmol, 1 equiv.) was cooled to 0°C using an ice/water bath. Dess-Martin reagent (12 mmol,1.2 equiv.) was added, and the mixture was stirred for two hours while the bath gradually warmed to room temperature. Next, a saturated aqueous solution of $NaHCO_3$ and $Na_2S_2O_3$ (in a 1:1 ratio, 60 mL) was added, and the mixture was stirred for an additional two hours until the aqueous and organic layers separated. The organic phase was dried using Na_2SO_4 and then concentrated under vacuum to produce a yellow oil. The product was purified via column chromatography using an EtOAc/hexane solvent (1:20). **^{1}H NMR** (400 MHz, $CDCl_3$) δ 7.66-7.59 (m, 1H), 7.49 – 7.41 (m, 2H), 7.33 (ddd, *J* = 8.3, 7.1, 1.2 Hz, 1H), 6.94 (dd, *J* = 17.5, 11.0 Hz, 1H), 6.77 (dd, *J* = 17.5, 10.5 Hz, 1H), 6.15 (dd, *J* = 17.5, 1.2 Hz, 1H), 6.01 (dd, *J* = 10.6, 1.2 Hz, 1H), 5.70 (dd, *J* = 17.4, 1.1 Hz, 1H), 5.33 (dd, *J* = 11.0, 1.1 Hz, 1H). **^{13}C NMR** (100 MHz, $CDCl_3$) δ 196.60, 137.48, 137.08, 136.77, 134.75, 131.78, 130.98, 128.49, 127.38, 126.60, 116.87.

Preparation of $Co^{III}(dmgH)_2(4\text{-}NMe_2Py)Cl$

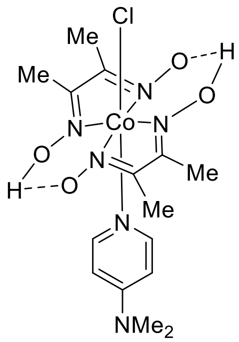

In a 100 mL flask, 500 mg (1.48 mmol) of [Co(dmgH)$_2$Cl$_2$] and 50 mL of methanol were added, followed by the addition of 4-Dimethylaminopyridine (361 mg, 2.96 mmol, 2 equiv.). The complex was dissolved within an hour. After filtration, the resulting precipitate was washed with 10 mL each of water, ethanol, and diethyl ether, yielding the desired product. **^{1}H NMR** (400 MHz, CDCl$_3$) δ 7.59 (d, *J* = 7.5 Hz, 2H), 6.28 (d, *J* = 7.1 Hz, 2H), 2.96 (s, 6H), 2.37 (s, 12H). **^{13}C NMR** (125 MHz, CDCl$_3$) δ 154.67, 152.08, 148.95, 108.24, 39.27, 13.02.

1.2 Characterization methods

Scanning electron microscope (SEM) images of the membranes were observed via a field-emission scanning electron microscope (FESEM, JSM-7610F, JEOL). The crystal phase was characterized by X-ray diffraction (XRD) on an X-ray powder diffractometer (Rigaku MiniFlex 600) at a scan rate of 3° min^{-1}. Fourier-transform infrared spectroscopy (FTIR) spectra were obtained with a Nicolet 6700 FTIR spectrometer. The UV-Vis absorption spectra were tested through a UV3600 instrument. The product mixtures were analyzed by thin layer chromatography (TLC) using TLC silica gel plates (Merck Schuchardt) with a fluorescent indicator (λ = 254 nm). The purification of the products was performed by flash column chromatography using silica gel 60 (63-200 μm) from SANPONT. ^{1}H NMR, ^{13}C NMR, ^{31}P NMR, and ^{19}F NMR spectra were recorded on a Bruker AV-III400 (400 MHZ) or AMX500 (500 MHz) spectrometer. Chemical shifts were calibrated using residual undeuterated solvent as an internal reference (CDCl$_3$: 7.26 ppm ^{1}H NMR, 77.16 ppm ^{13}C NMR). Multiplicity was indicated as follows: s (singlet), d (doublet), t (triplet), q (quartet), m (multiplet), dd (doublet of doublet), td (triplet of doublets), dt (doublet of triplets), ddd (doublet of doublets of doublets), brs (broad singlet). All high-resolution mass spectra (HRMS) were obtained on a Finnigan/MAT95XL-T spectrometer. Analytic high performance liquid chromatography (HPLC) was performed using a Shimadzu Prominence System equipped with a Welch Ultimate@ XB-C18 column (10 μM, 250 mm×4.60 mm i.d.) at room temperature in a mixed solvent system of water and methanol. Chiral HPLC analysis was performed on a Shimadzu Prominence System equipped with chiralpak IC columns in a mixed solvent system of n-hexane and isopropanol (Hexane/*i*PrOH = 70/30, 1.0 mL/min, 30 °C).

XRD experiments for NaDT

A specimen of $H_{22}Na_4O_{43}W_{10}$, approximate dimensions 0.044 mm × 0.073 mm × 0.114 mm, was used for the X-ray crystallographic analysis. The X-ray intensity data were measured (λ = 0.71073 Å). The total exposure time was 0.64 hours. The frames were integrated with the Bruker SAINT software package using a narrow-frame algorithm. The

integration of the data using a monoclinic unit cell yielded a total of 172686 reflections to a maximum θ angle of 30.57° (0.70 Å resolution), of which 11200 were independent (average redundancy 15.418, completeness = 99.5%, R_{int} = 7.26%, R_{sig} = 2.79%) and 9362 (83.59%) were greater than $2\sigma(F^2)$. The final cell constants of *a* = 11.4274(4) Å, *b* = 15.8405(5) Å, *c* = 21.0258(8) Å, β = 105.5440(10)°, volume = 3666.8(2) $Å^3$, are based upon the refinement of the XYZ-centroids of 9760 reflections above 20 σ(I) with 6.942° < 2θ < 61.06°. Data were corrected for absorption effects using the Multi-Scan method (SADABS). The ratio of minimum to maximum apparent transmission was 0.546.

The structure was solved and refined using the Bruker SHELXTL Software Package, using the space group P 1 21/n 1, with Z = 4 for the formula unit, $H_{22}Na_4O_{43}W_{10}$. The final anisotropic full-matrix least-squares refinement on F2 with 580 variables converged at R1 = 2.03%, for the observed data and wR2 = 3.96% for all data. The goodness-of-fit was 1.054. The largest peak in the final difference electron density synthesis was 1.178 $e^-/Å^3$, and the largest hole was -1.503 $e^-/Å^3$ with an RMS deviation of 0.291 $e^-/Å^3$. On the basis of the final model, the calculated density was 4.783 g/cm^3 and F(000), 4600 e^-.

2. Experimental

2.1 Aryl amination via single-electron transfer (SET)

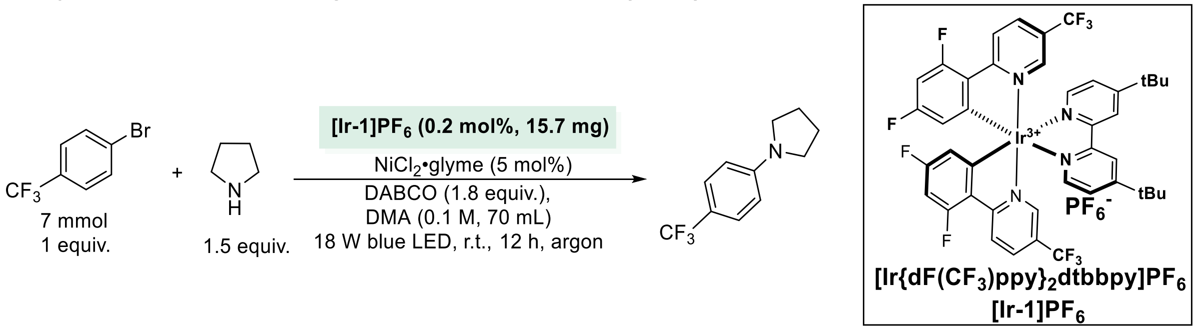

A solution of DMA (70 mL) with photocatalyst **[Ir-1]PF6** (15.7 mg, 0.014 mmol, 0.2 mol%), $NiBr_2$·glyme (12.6 mg, 0.35 mmol, 5 mol%), and DABCO (1.41 g, 12.6 mmol, 1.8 equiv.) in a 100 mL round bottom flask (RBF) was bubbled with argon for 15 min. Then 4-bromobenzotrifluoride (980 µL, 7 mmol, 1.0 equiv.) and pyrrolidine (877 µL, 10.5 mmol, 1.5 equiv.) were added to the above solution. The reaction was irradiated by 18 W blue LEDs under an argon atmosphere and maintained at ambient temperature by cooling with a fan. After 12 h, 1 mL reaction mixture was taken out to examine the yield using dibromomethane as an internal standard. The remaining solution was filtered through the COF Tp-TAPB membrane under an argon atmosphere (2 bar). After the nanofiltration, the COF membrane, together with the retentate, was washed with DMA (5×10 mL) under sonication for 30 min, and the solution of the recovered photocatalyst was obtained. The recovery rate of the photocatalyst was measured by UV-Vis absorption. A total volume of 70 mL of DMA solution containing the recovered photocatalyst was placed in a 100 mL RBF, bubbled with argon for 15 min, and used for the next cycle. Ten cycles of reactions and separations were conducted. The product yield in each cycle was determined by crude ^1H NMR spectra using CH_2Br_2 as an internal standard (**Supplementary Figs. 17** and **20**). The recovery rate of the photocatalyst is shown in **Supplementary Fig. 18**. The

recovered photocatalyst was analyzed by ^{1}H, ^{19}F, and ^{31}P NMR (**Supplementary Figs. 21-23**).

The spectral data of product 1-(4-(trifluoromethyl)phenyl)pyrrolidine matched that in the reported literature[6]:

^{1}H NMR (400 MHz, $CDCl_3$) δ 7.44 (d, *J* = 8.2 Hz, 2H), 6.56 (d, *J* = 8.8 Hz, 2H), 3.35 – 3.28 (m, 4H), 2.08 – 1.99 (m, 4H).

^{13}C NMR (125 MHz, $CDCl_3$) δ 149.88, 127.59, 126.53, 126.48, 126.43, 123.67, 116.96, 116.47, 110.95, 47.65, 25.60.

^{19}F NMR (377 MHz, $CDCl_3$) δ –60.6 (s).

The spectral data of recovered photocatalyst **[Ir-1]PF_6** after ten cycles:

^{1}H NMR (400 MHz, Acetone-*d_6*) δ 8.93 (d, *J* = 2.2 Hz, 2H), 8.61 (dd, *J* = 8.5, 2.4 Hz, 2H), 8.40 (dd, *J* = 8.5, 2.4 Hz, 2H), 8.18 (d, J = 5.9 Hz, 2H), 7.84 – 7.77 (m, 4H), 6.86 (ddd, *J* = 12.2, 9.3, 2.4 Hz, 2H), 5.97 (dd, *J* = 8.4, 2.4 Hz, 2H), 1.43 (s, 18H).

^{19}F NMR (377 MHz, Acetone-*d_6*) δ -63.69, -72.67 (d, *J* = 707.2 Hz), -104.75 (d, *J* = 12.0 Hz), -108.07 (d, *J* = 12.1 Hz).

^{31}P NMR (162 MHz, Acetone-*d_6*) δ -135.53 – -152.99 (hept, *J* = 707.13).

2.2 Intermolecular cycloaddition via energy transfer (EnT)

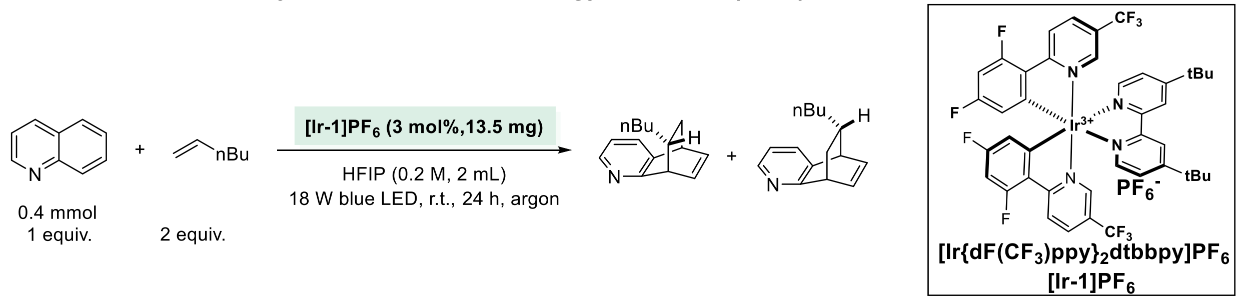

A mixture of quinoline (51.6 mg, 0.4 mmol, 1 equiv.) and **[Ir-1]PF_6** (13.5 mg, 3 mol%) in HFIP (2 mL, 0.2 M) was degassed by sparging with argon for 5 min in a 10 mL Schlenk tube. After adding 1-hexene (101 μL, 0.8 mmol, 2 equiv.), the reaction was stirred and irradiated with 18 W blue LEDs. After the reaction, 18 mL HFIP was added to the solution to obtain a diluted mixture. The mixture was then filtered through the COF Tp-TAPB membrane under an argon atmosphere (2 bar). After the nanofiltration, the COF membrane, together with the retentate, was washed with EtOAc (5×10 mL) under sonication for 30 min. The resulting solution was analyzed by UV-Vis absorption to calculate the recovery rate of the **[Ir-1]PF_6**. The solution was concentrated by rotary evaporation and then dried under vacuum. The recovered **[Ir-1]PF_6** was used for the next cycle. The permeate solution after nanofiltration was evaporated under reduced pressure. Dibromomethane (14 μL, 0.2 mmol) was added to the residue for crude ^{1}H NMR measurement in $CDCl_3$ to determine the yield. Ten cycles of reactions and separations were conducted. The product yield in each cycle was determined by crude ^{1}H NMR spectra using CH_2Br_2 as an internal standard (**Supplementary Figs. 28** and **31**). The recovery rate of the photocatalyst is shown in **Supplementary Fig. 29**. The recovered photocatalyst was analyzed by ^{1}H, ^{19}F, and ^{31}P NMR (**Supplementary Figs. 32-34**).

The spectral data of products matched that in the reported literature[7]:

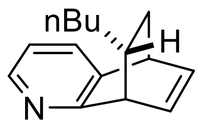

^{1}H NMR (400 MHz, $CDCl_3$) δ 8.22 (dd, *J* = 5.1, 1.6 Hz, 1H), 7.35 (dd, *J* = 7.4, 1.7 Hz, 1H), 6.99 (dd, *J* = 7.3, 5.1 Hz, 1H), 6.59 (ddd, *J* = 7.7, 6.3, 1.5 Hz, 1H), 6.44 (ddd, *J* = 7.6, 6.0, 1.4 Hz, 1H), 3.94 – 3.89 (m, 1H), 3.86 (dt, *J* = 4.4, 1.6 Hz, 1H), 1.93 – 1.79 (m, 2H), 1.38 – 1.28 (m, 1H), 1.22 – 1.15 (m, 3H), 1.05 – 0.96 (m, 1H), 0.89 (ddd, *J* = 11.1, 3.8, 2.2 Hz, 2H), 0.81 (t, *J* = 7.1 Hz, 3H), 0.74 – 0.63 (m, 1H).
^{13}C NMR (125 MHz, $CDCl_3$) δ 162.82, 145.08, 137.92, 135.87, 134.41, 129.06, 120.49, 47.86, 39.72, 37.86, 36.17, 33.69, 30.16, 22.81, 14.15.

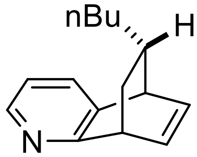

^{1}H NMR (400 MHz, $CDCl_3$) δ 8.22 (dd, *J* = 5.1, 1.6 Hz, 1H), 7.36 (dd, *J* = 7.3, 1.6 Hz, 1H), 6.99 (dd, *J* = 7.3, 5.1 Hz, 1H), 6.59 – 6.45 (m, 2H), 4.00 (dq, *J* = 5.2, 2.3 Hz, 1H), 3.73 (dt, *J* = 5.9, 2.0 Hz, 1H), 1.90 (ddd, *J* = 11.7, 9.8, 3.1 Hz, 1H), 1.87 – 1.77 (m, 2H), 1.26 – 1.15 (m, 8H), 0.96 (ddd, *J* = 11.7, 4.4, 2.4 Hz, 2H), 0.94 – 0.86 (m, 2H), 0.82 (t, *J* = 7.1 Hz, 4H), 0.82 – 0.69 (m, 2H).
^{13}C NMR (125 MHz, $CDCl_3$) δ 164.90, 145.25, 136.05, 135.11, 134.36, 131.54, 120.28, 44.56, 43.26, 37.74, 35.96, 32.89, 29.84, 22.84, 14.20.

The spectral data of recovered photocatalyst **[Ir-1]PF_6** after ten cycles:
^{1}H NMR (400 MHz, Acetone-d_6) δ 8.93 (d, *J* = 1.8 Hz, 2H), 8.61 (dd, *J* = 8.9, 2.7 Hz, 2H), 8.40 (dd, *J* = 8.8, 2.1 Hz, 2H), 8.17 (d, *J* = 5.9 Hz, 2H), 7.84 – 7.76 (m, 4H), 6.85 (ddd, *J* = 12.8, 9.3, 2.3 Hz, 2H), 5.96 (dd, *J* = 8.4, 2.4 Hz, 2H), 1.42 (s, 18H).
^{19}F NMR (377 MHz, Acetone-d_6) δ -63.67, -72.64 (d, *J* = 707.4 Hz), -104.73 (d, *J* = 11.9 Hz), -108.04 (d, *J* = 12.1 Hz).
^{31}P NMR (162 MHz, Acetone-d_6) δ -135.53 – -153.00 (hept, *J* = 707.53).

2.3 Intramolecular cycloaddition via EnT

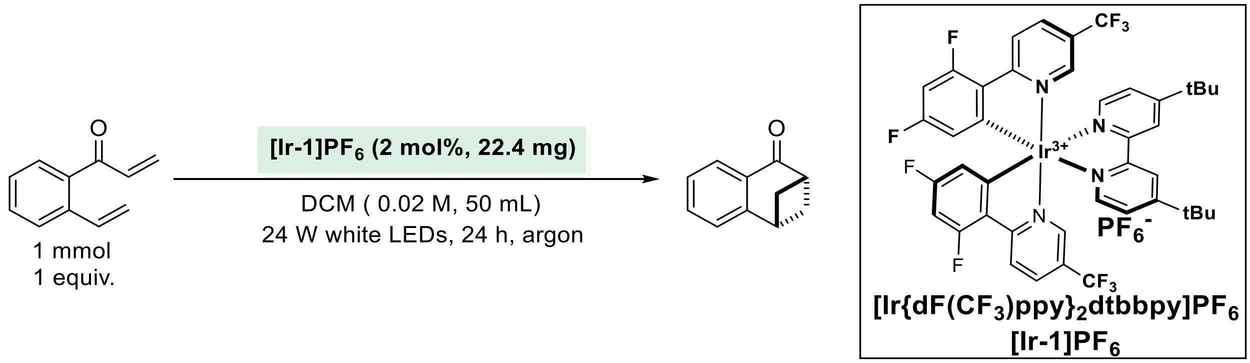

A mixture of 1-(2-vinylphenyl)-propenone (158 mg, 1 mmol, 1 equiv.), **[Ir-1]PF_6** (22.4 mg, 2 mol%), and DCM (50 mL, 0.02 M) in a 100 mL RBF was degassed by sparging with argon for 5 min in an ice/water bath. The solution was stirred vigorously under irradiation with 24 W white LEDs. After 24 hours of reaction, the mixture was filtered through the COF Tp-TAPB membrane under an argon atmosphere (2 bar). After the nanofiltration,

the COF membrane, together with the retentate, was washed with EtOAc (5×10 mL) under sonication for 30 min. The resulting solution was analyzed by UV-Vis absorption to calculate the recovery rate of the photocatalyst. Then the solution was concentrated by rotary evaporation and dried under vacuum. The recovered **[Ir-1]PF$_6$** was used for the next cycle. The permeate solution after nanofiltration was evaporated under reduced pressure. Dibromomethane (70 μL, 1 mmol) was added to the residue for crude ^{1}H NMR measurement in $CDCl_3$ to determine the yield. A total of 10 cycles of reactions and separations were conducted. The product yield in each cycle was determined by crude ^{1}H NMR spectra using CH_2Br_2 as an internal standard (**Supplementary Figs. 38** and **41**). The recovery rate of the photocatalyst is shown in **Supplementary Fig. 39**. The recovered photocatalyst was analyzed by ^{1}H, ^{19}F, and ^{31}P NMR (**Supplementary Figs. 42-44**).

The spectral data of the product matched that in the reported literature[5]:

^{1}H NMR (400 MHz, $CDCl_3$) δ 8.01 – 7.92 (m, 1H), 7.42 (td, *J* = 7.4, 1.5 Hz, 1H), 7.33 (td, *J* = 7.5, 1.3 Hz, 1H), 7.24 – 7.17 (m, 1H), 3.30 (q, *J* = 5.7 Hz, 1H), 3.21 (q, *J* = 5.8 Hz, 1H), 2.97 – 2.89 (m, 2H), 2.37 – 2.32 (m, 2H).

^{13}C NMR (125 MHz, $CDCl_3$) δ 201.88, 151.92, 133.31, 128.70, 127.19, 126.73, 125.03, 49.74, 44.04, 40.23.

The spectral data of recovered photocatalyst **[Ir-1]PF$_6$** after ten cycles:

^{1}H NMR (400 MHz, Acetone-d_6) δ 8.92 (d, *J* = 2.1 Hz, 2H), 8.61 (dd, *J* = 8.8, 2.8 Hz, 2H), 8.40 (dd, *J* = 8.7, 2.2 Hz, 2H), 8.17 (d, *J* = 6.0 Hz, 2H), 7.83 – 7.76 (m, 4H), 6.85 (ddd, *J* = 12.7, 9.3, 2.3 Hz, 2H), 5.95 (dd, *J* = 8.4, 2.4 Hz, 2H), 1.42 (s, 18H).

^{19}F NMR (377 MHz, Acetone-d_6) δ -63.66, -72.61 (d, *J* = 707.3 Hz), -104.72 (d, *J* = 12.1 Hz), -108.00 (d, *J* = 11.7 Hz).

^{31}P NMR (162 MHz, Acetone-d_6) δ -135.55 – -153.02 (hept, *J* = 707.53).

2.4 Alkylation of heteroarenes via SET

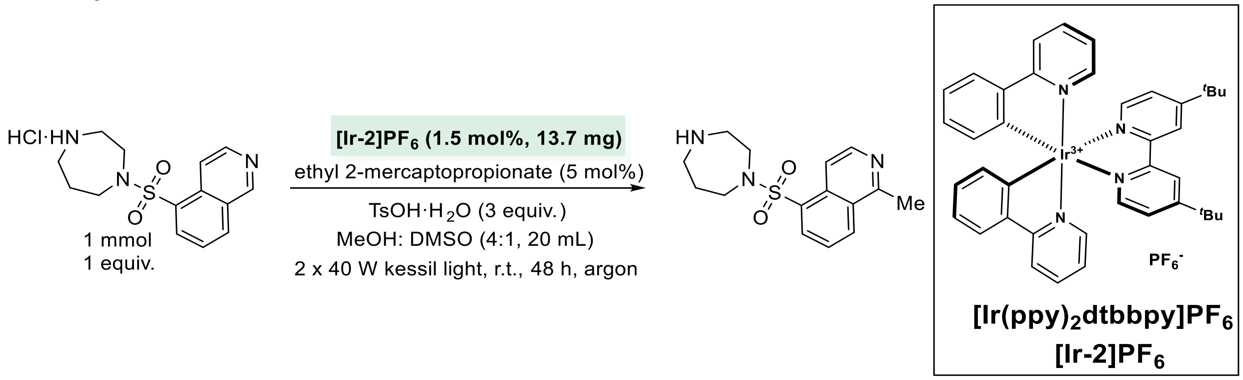

Fasudil monohydrochloride (327 mg, 1 mmol, 1.0 equiv.), **[Ir-2]PF$_6$** (13.7 mg, 1.5 mol%), *p*-toluenesulfonic acid monohydrate ($TsOH·H_2O$, 570 mg, 3 mmol, 3.0 equiv.), 16 mL MeOH, and 4 mL DMSO were added into a 50 mL Schlenk tube. The reaction mixture was degassed by sparging with argon for 10 min. After adding ethyl 2-mercaptopropionate (5 mol%), the mixture was irradiated with 2×40 W blue LED at room temperature with a fan for cooling. After 48 hours of reaction, the mixture was filtered through the COF Tp-TAPB membrane under an argon atmosphere (2 bar). After the nanofiltration, the COF membrane, together with the retentate, was washed with EtOAc (5×10 mL) under sonication for 30 min. The resulting solution was analyzed by UV-Vis

absorption to calculate the recovery rate of photocatalyst. Then the solution was concentrated by rotary evaporation and dried under vacuum. The recovered **[Ir-2]PF₆** was used for the next cycle. The permeate solution was diluted with 1 M sodium hydroxide aqueous solution (4 mL) and DCM (30 mL), washed with brine (3×10 mL), dried over sodium sulfate, and concentrated under reduced pressure. Dibromomethane was added to the residue for crude ^{1}H NMR in $CDCl_3$ to determine the yield. Ten cycles of reactions and separations were conducted. The product yield in each cycle was determined by crude ^{1}H NMR spectra using CH_2Br_2 as an internal standard (**Supplementary Figs. 48** and **51**). The recovery rate of the photocatalyst is shown in **Supplementary Fig. 49**. The recovered photocatalyst was analyzed by ^{1}H, ^{19}F, and ^{31}P NMR (**Supplementary Figs. 52-54**).

The spectral data of the product matched that in the reported literature[8]:

^{1}H NMR (500 MHz, $CDCl_3$): δ 8.53 (d, *J* = 6.2 Hz, 1H), 8.37 (d, *J* = 8.5 Hz, 1H), 8.33 (d, *J* = 8.0 Hz, 1H), 8.30 (d, *J* = 7.2 Hz, 1H), 7.66 (t, *J* = 7.9 Hz, 1H), 3.48 (t, *J* = 6.1 Hz, 2H), 3.43 (dd, *J* = 6.4, 4.1 Hz, 2H), 3.02 (s, 3H), 2.96 (dd, *J* = 6.3, 4.2 Hz, 2H), 2.93 (t, *J* = 5.8 Hz, 2H), 1,86 (br s, 1H),1.85 – 1.81 (m, 2H).

^{13}C NMR (125 MHz, $CDCl_3$): δ 159.39, 143.65, 135.03, 132.42, 131.81, 131.01, 128.05, 125.31, 116.00, 51.18, 50.24, 47.67, 47.35, 31.22, 23.02.

The spectral data of recovered photocatalyst **[Ir-2]PF₆** after ten cycles:

^{1}H NMR (400 MHz, Acetone-*d*6) δ 8.88 (d, *J* = 2.0 Hz, 2H), 8.23 (d, *J* = 8.3 Hz, 2H), 8.01 – 7.92 (m, 4H), 7.89 (d, *J* = 7.9 Hz, 2H), 7.79 (dt, *J* = 5.8, 1.3 Hz, 2H), 7.70 (dd, *J* = 5.9, 1.9 Hz, 2H), 7.13 (ddd, *J* = 7.3, 5.8, 1.4 Hz, 2H), 7.03 (td, *J* = 7.5, 1.2 Hz, 2H), 6.91 (td, *J* = 7.4, 1.3 Hz, 2H), 6.34 (dd, *J* = 7.6, 1.2 Hz, 2H), 1.41 (s, 18H).

^{19}F NMR (377 MHz, Acetone-*d*6) δ -72.59 (d, *J* = 707.6 Hz).

^{31}P NMR (162 MHz, Acetone-*d*6) δ -135.53 – -152.99 (hept, *J* = 707.13).

2.5 Aromatic C−H thiolation via SET

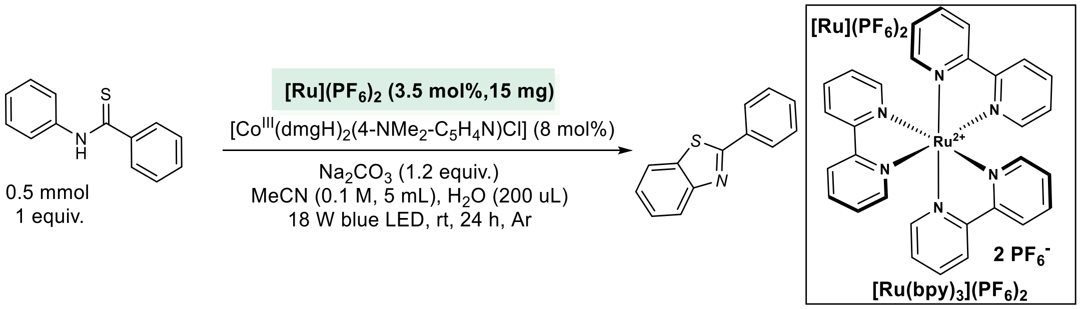

Under an argon atmosphere, a mixture of *N*-phenylbenzothioamide (106.5 mg, 0.5 mmol, 1 equiv.), $Co^{III}(dmgH)_2(4\text{-}NMe_2Py)Cl$ (17.8 mg, 8 mol%), **[Ru](PF₆)₂** (15 mg, 3.5 mol%), Na_2CO_3 (64 mg, 0.6 mmol, 1.2 equiv.), H_2O (200 μL), and degassed dry MeCN (5 mL) in a 10 mL Schlenk tube was stirred under irradiation of 18 W blue LED for 24 hours. After completion of the reaction, the solid was filtered off by filter paper and washed with 5 mL MeCN. The resulting homogenous solution was filtered through the COF Tp-HZ membrane under an argon atmosphere (4 bar). After nanofiltration, the COF membrane, together with the retentate, was washed with EtOAc (5×10 mL) under sonication for 30 min. The resulting solution was analyzed by UV-Vis absorption to calculate the recovery rate of photocatalyst. Then the solution was concentrated by rotary evaporation and dried under vacuum. The recovered **[Ru](PF₆)₂** was used for the next

cycle. The permeate solution after nanofiltration was evaporated under reduced pressure. The yield was analyzed through crude ^1H NMR measurement in $CDCl_3$ using 1,3,5-trimethoxybenzene (28 mg, 0.167 mmol) as an internal standard. Ten cycles of reactions and separations were conducted. The product yield in each cycle was determined by crude ^1H NMR spectra using trimethoxybenzene as an internal standard (**Supplementary Figs. 58** and **61**). The recovery rate of the photocatalyst is shown in **Supplementary Fig. 59**. The recovered photocatalyst was analyzed by ^1H, ^{19}F, and ^{31}P NMR (**Supplementary Figs. 62-64**).

The spectral data of the product 2-phenylbenzo[d]thiazole matched that in the reported literature[4]:

^{1}H NMR (400 MHz, $CDCl_3$) δ 8.13 – 8.07 (m, 3H), 7.91 (d, J = 7.9 Hz, 1H), 7.52 – 7.48 (m, 4H), 7.41 – 7.37 (m, 1H).

^{13}C NMR (100 MHz, $CDCl_3$) δ 168.14, 154.23, 135.15, 133.70, 131.04, 129.10, 127.64, 126.40, 125.27, 123.32, 121.70.

The spectral data of recovered photocatalyst **[Ru](PF_6)$_2$** after ten cycles:

^{1}H NMR (400 MHz, Acetone-d_6) δ 8.81 (dd, J = 8.1, 1.2 Hz, 6H), 8.20 (td, J = 7.9, 1.5 Hz, 6H), 8.05 (dd, J = 5.6, 1.5 Hz, 6H), 7.57 (ddd, J = 7.2, 5.6, 1.3 Hz, 6H).

^{19}F NMR (377 MHz, Acetone-d_6) δ -67.27 (d, J = 707.8 Hz).

^{31}P NMR (162 MHz, Acetone-d_6) δ -130.34 – -147.82 (hept, J = 707.94).

2.6 Enantioselective Minisci-type addition via SET

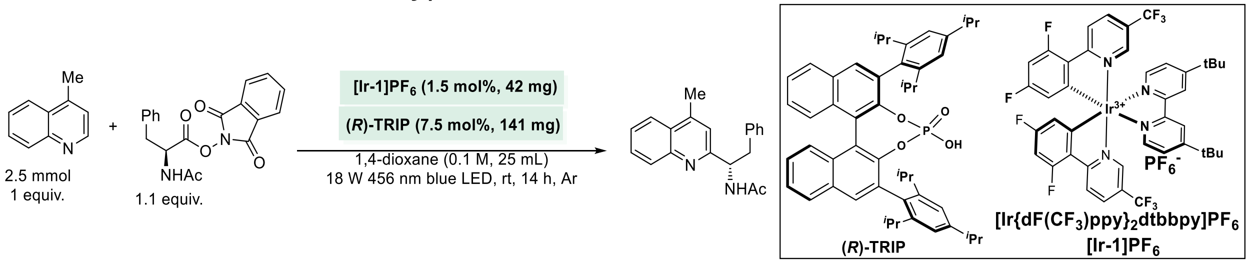

A mixture of 4-methylquinoline (338 µL, 2.5 mmol, 1.0 equiv.), 1,3-dioxoisoindolin-2-yl acetyl-L-phenylalaninate (969 mg, 2.75 mmol, 1.1 equiv.), **[Ir-1]PF_6** (42 mg, 1.5 mol%), (R)-TRIP (141 mg, 7.5 mol%), and 1,4-dioxane (25 mL) in a 100 mL RBF was degassed by sparging with argon for 5 min. The reaction was irradiated with 18 W 456 nm blue LEDs and maintained at room temperature with a fan. After 14 hours, the mixture was filtered through the COF Tp-TAPB membrane under an argon atmosphere (2 bar). After nanofiltration, the COF membrane, together with the retentate, was washed with EtOAc (5×10 mL) under sonication for 30 min. The resulting solution was analyzed by UV-Vis absorption to calculate the recovery rate. Then the retentate was concentrated by rotary evaporation and dried under vacuum. The recovered **[Ir-1]PF_6** and (R)-TRIP were used for the next cycle. The permeate solution after nanofiltration was evaporated under reduced pressure. Dibromomethane (175 µL, 2.5 mmol) was added to the residue for crude ^1H NMR measurement in $CDCl_3$ to determine the yield. Ten cycles of reactions and separations were conducted. The product yield in each cycle was determined by crude ^1H NMR spectra using CH_2Br_2 as an internal standard (**Supplementary Figs. 68** and **71**). The recovery rate of the photocatalyst is shown in **Supplementary Fig. 69**. The recovered photocatalyst was analyzed by ^1H, ^{19}F, and ^{31}P NMR (**Supplementary Figs. 73-75**).

The spectral data of the product matched that in the reported literature[3]:
^{1}H NMR (400 MHz, $CDCl_3$) δ 8.02 (dd, *J* = 8.5, 1.3 Hz, 1H), 7.95 (dd, *J* = 8.4, 1.4 Hz, 1H), 7.69 (ddd, *J* = 8.4, 6.8, 1.4 Hz, 1H), 7.54 (ddd, *J* = 8.3, 6.8, 1.3 Hz, 1H), 7.23 (br s, 1H), 7.15 (dt, *J* = 4.5, 1.7 Hz, 3H), 6.98 – 6.91 (m, 2H), 6.80 (d, *J* = 1.1 Hz, 1H), 5.39 (td, *J* = 7.7, 5.2 Hz, 1H), 3.35 (dd, *J* = 13.3, 5.2 Hz, 1H), 3.17 (dd, *J* = 13.3, 7.9 Hz, 1H), 2.58 (s, 3H), 2.07 (s, 3H).
^{13}C NMR (100 MHz, $CDCl_3$) δ 169.53, 158.87, 147.28, 144.58, 137.36, 129.81, 129.59, 129.35, 128.19, 127.53, 126.52, 126.22, 123.92, 121.63, 55.59, 42.29, 23.68, 18.75.
The spectral data of recovered catalysts of **[Ir-1]PF_6** and (*R*)-TRIP after ten cycles:
^{1}H NMR (400 MHz, Acetone-*d*$_6$) δ 8.11 (d, *J* = 8.1 Hz, 2H), 8.01 (s, 2H), 7.55 (t, *J* = 7.5 Hz, 2H), 7.35 (td, *J* = 7.6, 6.7, 1.3 Hz, 2H), 7.24 (d, *J* = 8.5 Hz, 2H), 7.20 (s, 2H), 7.13 (s, 2H), 2.98 (p, *J* = 6.9 Hz, 2H), 2.83 (p, *J* = 6.7 Hz, 2H), 2.71 (p, *J* = 6.8 Hz, 2H),1.31 (d, *J* = 6.9 Hz, 12H), 1.21 (dd, *J* = 9.0, 6.7 Hz, 12H), 1.12 (d, *J* = 6.8 Hz, 6H), 0.96 (d, *J* = 6.7 Hz, 6H). (for (*R*)-TRIP)
^{1}H NMR (400 MHz, Acetone-*d*$_6$) δ 8.93 (d, *J* = 2.1 Hz, 2H), 8.62 (dd, *J* = 9.0, 2.8 Hz, 2H), 8.40 (dd, *J* = 8.9, 2.2 Hz, 2H), 8.18 (d, *J* = 5.8 Hz, 2H), 7.83 – 7.80 (m, 4H), 6.85 (ddd, *J* = 11.8, 9.3, 2.4 Hz, 2H), 5.97 (dd, *J* = 8.5, 2.4 Hz, 2H), 1.42 (s, 18H). (for **[Ir-1]PF_6**)
^{19}F NMR (377 MHz, Acetone-*d*$_6$) δ -63.64, -72.63 (d, *J* = 707.7 Hz), -104.65 (d, *J* = 11.8 Hz), -107.99 (d, *J* = 11.9 Hz). (for **[Ir-1]PF_6**)
^{31}P NMR (162 MHz, Acetone-*d*$_6$) δ 0.76. (for (*R*)-TRIP)
^{31}P NMR (162 MHz, Acetone-*d*$_6$) δ -135.52 – -152.98 (hept, *J* = 707.13). (for **[Ir-1]PF_6**)

2.7 Large-scale photocatalyst recovery

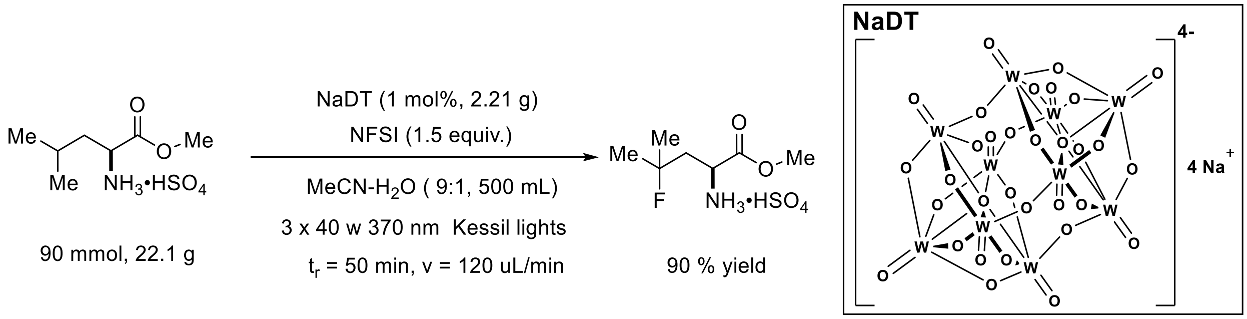

(S)-Methyl-2-amino-4-methylpentanoate sulfate (22.1 g, 90 mmol), N-fluorobenzenesulfonimide (NFIS, 1.5 equiv.), and sodium decatungstate (NaDT, 2.21 g, 1 mol%) were dissolved in 500 mL mixed solution of MeCN-H_2O (9:1) then degassed by argon sparging for 15 min. The homogenous reaction mixture was pumped via syringe pump through a photoreactor (3×40 W 370 nm Kessil lights, total volume of 6 mL, 1/16 inch I.D. tubing) at 120 µL/min (residence time of 50 min) and collected in a receiving bottle. The 1H NMR yield was analysed by taking 0.3 mL sample diluting with 0.3 mL CD_3CN/D_2O (9/1). After the reaction, the collected mixture was filtered through the COF Tp-DHBD membrane under argon (4 bar). After nanofiltration, the COF membrane, together with the retentate, was washed with water (5×20 mL) under sonication for 1 h. The recovered catalyst was obtained by vacuum drying and weighted to calculate the recovery rate (recovered NaDT 2.1g, 95% recovery rate). The permeate solution was concentrated to around 100 mL and dried by azeotropic distillation with 2-MeTHF (2×200 mL). The product was precipitated by adding 2-MeTHF (500 mL), filtered, washed with 2-MeTHF, and dried under a nitrogen stream. The product yield was determined by crude

^{1}H NMR spectra (96%, **Supplementary Figs. 79**). (*S*)-methyl 2-amino-4-fluoro-4-methylpentanoate sulfate was isolated as a white amorphous solid with a yield of 90%. The recovered photocatalyst was analyzed by UV-Vis spectra (**Supplementary Figs. 80**).

2.8 Stepwise separation of photocatalysts and products

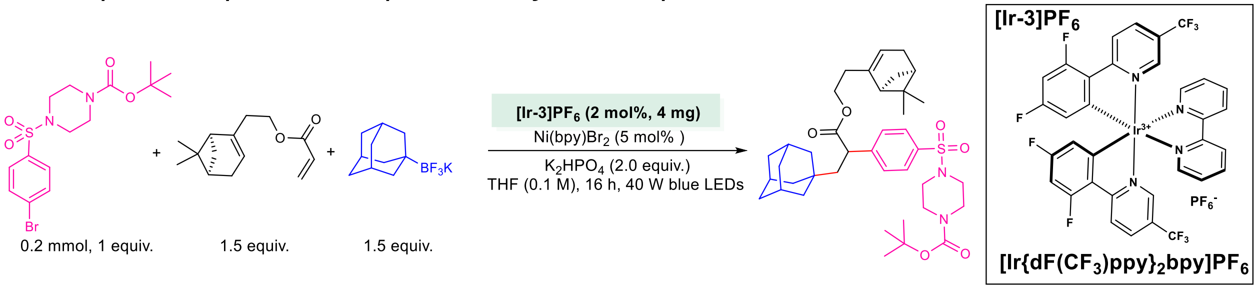

1-Adamantane trifluoroborate potassium salt (72.6 mg, 0.3 mmol, 1.5 equiv.), *tert*-butyl 4-((4-bromophenyl)sulfonyl)piperazine-1-carboxylate (81 mg, 0.2 mmol, 1 equiv.), K_2HPO_4 (69 mg, 0.4 mmol, 2 equiv.), Ni(bpy)Br_2 (3.8 mg, 5 mol %), and **[Ir-3]PF$_6$** (4.0 mg, 2 mol%) were added into a 10 mL Schlenk tube. The tube was sealed, evacuated, and backfilled with argon three times. After adding 2-((1*S*,5*R*)-6,6-dimethylbicyclo[3.1.1]hept-2-en-2-yl)ethyl acrylate (66 mg, 0.3 mmol, 1.5 equiv.) and 2 mL degassed THF, the reaction was irradiated with 18 W, 456 nm blue LEDs for 12 hours at room temperature. The mixture was passed through a pad of Celite® and eluted with THF to remove the K_2HPO_4 salt. The yield was analyzed through crude ^{1}H NMR measurement in $CDCl_3$ using dibromomethane as an internal standard.

After the reaction, the resulting solution was totally diluted to 10 mL and filtered through a COF Tp-TAPB membrane under argon (4 bar) to selectively separate the photocatalyst **[Ir-3]PF$_6$** from other components. After nanofiltration, the COF membrane, together with the retentate, was washed with THF (5×10 mL), and the resulting solution was analyzed by UV-Vis absorption to calculate the recovery rate of **[Ir-3]PF$_6$**. The recovered catalyst was dried and analyzed by NMR measurements. The permeate solution from the first-step nanofiltration was further filtered through a COF Tp-HZ membrane under argon (4 bar) to purify the product from the reactant residues. After nanofiltration, the COF membrane, together with the retentate, was washed with THF (5×10 mL) under sonication for 30 min. The yield of the product was analyzed through the crude ^{1}H NMR in $CDCl_3$ using dibromomethane as an internal standard. The purity of the product was analyzed by HPLC (**Supplementary Fig. 90**).The product yield in each cycle was determined by crude ^{1}H NMR spectra using CH_2Br_2 as an internal standard (**Supplementary Figs. 89**). The recovery rate of the photocatalyst is shown in **Supplementary Fig. 83**. The recovered photocatalyst was analyzed by ^{1}H, ^{19}F, and ^{31}P NMR (**Supplementary Figs. 85-87**).

NMR data analysis of the product:

^{1}H NMR (400 MHz, $CDCl_3$) δ 7.68 – 7.61 (m, 2H), 7.46 (dd, *J* = 8.5, 1.5 Hz, 2H), 5.16 (ddd, *J* = 15.7, 3.0, 1.5 Hz, 1H), 4.17 - 3.95 (m, 2H), 3.74 (dd, *J* = 9.0, 3.8 Hz, 1H), 3.48 (t, *J* = 5.1 Hz, 4H), 2.94 (t, *J* = 4.9 Hz, 4H), 2.31 - 2.27 (m, 1H), 2.24-2.19 (m, 2H), 2.17 - 2.11 (m, 2H), 2.07 - 2.01 (m, 1H), 2.01 - 1.95 (m, 1H), 1.92 (dd, *J* = 6.2, 3.3 Hz, 3H), 1.67 (d, *J* = 12.6 Hz, 3H), 1.58 (d, *J* = 12.5 Hz, 3H), 1.48 (dd, *J* = 12.1, 2.9 Hz, 3H), 1.40 (d, *J* = 6.5 Hz, 14H), 1.23 (d, *J* = 2.6 Hz, 3H), 1.03 (dd, *J* = 13.2, 8.6 Hz, 1H), 0.75 (dd, *J* = 9.0, 2.4 Hz, 3H).

^{13}C NMR (100 MHz, $CDCl_3$) δ 173.87, 154.26, 146.83, 143.92, 143.89, 134.12, 128.85, 128.13, 118.96, 118.91, 80.50, 63.34, 48.14, 48.08, 46.47, 45.94, 45.66, 45.61, 42.34, 40.74, 40.72, 38.06, 36.96, 35.86, 33.12, 31.67, 31.43, 28.61, 28.39, 27.01, 26.35, 21.21.
HRMS (ESI, m/z) calcd for $C_{39}H_{56}N_2NaO_6S$ $[M+Na]^+$: 703.3750, found: 703.3751.

NMR data analysis of the byproduct:
^{1}H NMR (400 MHz, $CDCl_3$) δ 5.31 – 5.25 (m, 2H), 4.06 (tt, J = 7.5, 3.7 Hz, 4H), 2.36 (dt, J = 8.6, 5.6 Hz, 2H), 2.30 – 2.19 (m, 11H), 2.05 (qd, J = 6.4, 5.6, 2.2 Hz, 3H), 1.94 (s, 6H), 1.72 – 1.57 (m, 13H), 1.44 (d, J = 2.9 Hz, 10H), 1.43 – 1.37 (m, 5H), 1.26 (s, 6H), 1.14 (d, J = 8.5 Hz, 2H), 0.82 (s, 6H).
^{13}C NMR (100 MHz, $CDCl_3$) δ 174.86, 144.33, 118.84, 62.72, 45.78, 42.15, 40.84, 39.12, 38.13, 37.20, 36.07, 32.03, 31.77, 31.48, 28.72, 28.34, 26.41, 21.25.
HRMS (ESI, m/z) calcd for $C_{48}H_{70}NaO_4$ $[M+Na]^+$: 733.5166, found: 733.5165.

The spectral data of recovered photocatalyst **$[Ir\text{-}3]PF_6$**:
^{1}H NMR (400 MHz, Acetone-d_6) δ 8.90 (d, *J* = 8.2 Hz, 2H), 8.62 (dd, *J* = 8.9, 2.8 Hz, 2H), 8.43 – 8.37 (m, 4H), 8.31 (ddd, *J* = 5.5, 1.6, 0.8 Hz, 2H), 7.98 (dt, *J* = 1.9, 0.9 Hz, 2H), 7.80 (ddd, *J* = 7.7, 5.5, 1.2 Hz, 2H), 6.86 (ddd, *J* = 12.8, 9.3, 2.3 Hz, 2H), 5.97 (dd, *J* = 8.5, 2.4 Hz, 2H).
^{19}F NMR (377 MHz, Acetone-d_6) δ -63.67, -72.74 (d, *J* = 707.3 Hz), -104.83 (d, *J* = 11.8 Hz), -108.08 (d, *J* = 12.1 Hz).
^{31}P NMR (162 MHz, Acetone-d_6) δ -135.62 – -153.10 (hept, *J* = 707.94).

Supplementary Figures

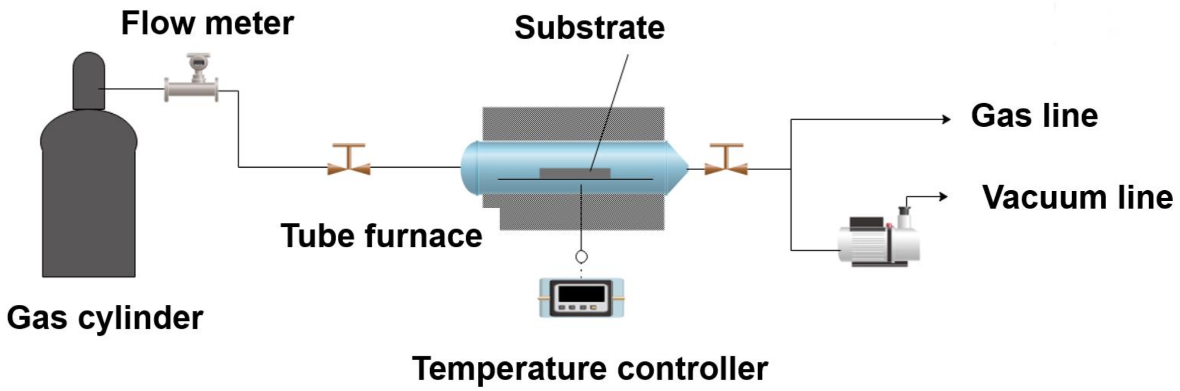

Supplementary Figure 1. A diagram demonstrating the carbonization process for PAN substrates.

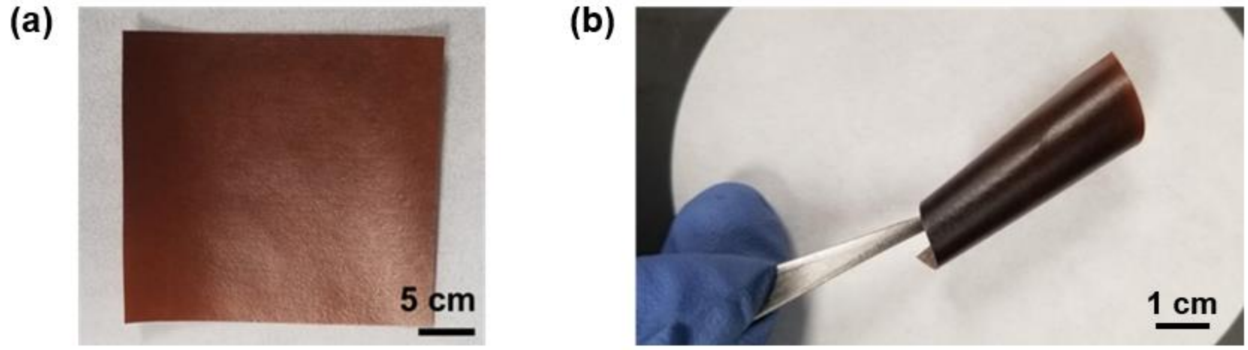

Supplementary Figure 2. Optical images of (a) carbonized PAN substrate and (b) twisted carbonized PAN substrate.

Note: The carbonized PAN substrate can be readily scaled up. Besides, it can be easily twisted, indicating its flexible and mechanically robust properties.

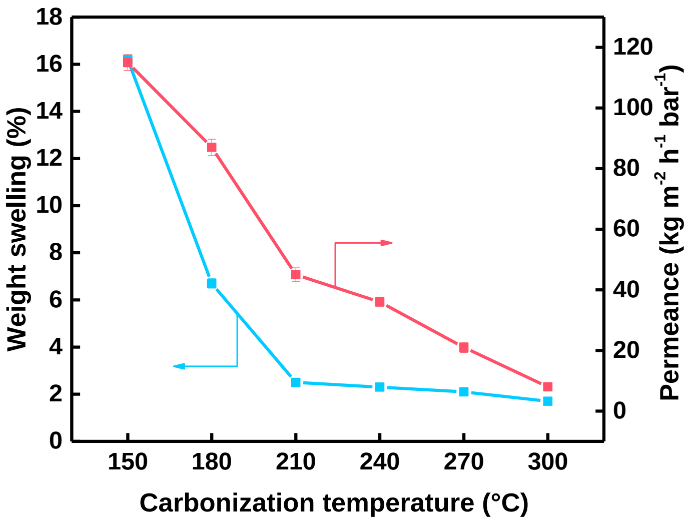

Supplementary Figure 3. The weight swelling in DMF and DMF permeance of the carbonized PAN substrates prepared under different carbonization temperatures. Error bars represent standard deviations for 3 measurements.

Note: The weight swelling first dramatically decreases and then keeps steady with the increase of carbonization temperature from 150 °C to 300 °C. The permeance of the PAN substrate also decreases with the increase in carbonization temperature because the high carbonization temperature results in dense polymeric networks with low porosity. Therefore, we chose 210 °C as the optimal carbonization temperature to obtain carbonized PAN substrates with both high solvent resistance and permeance.

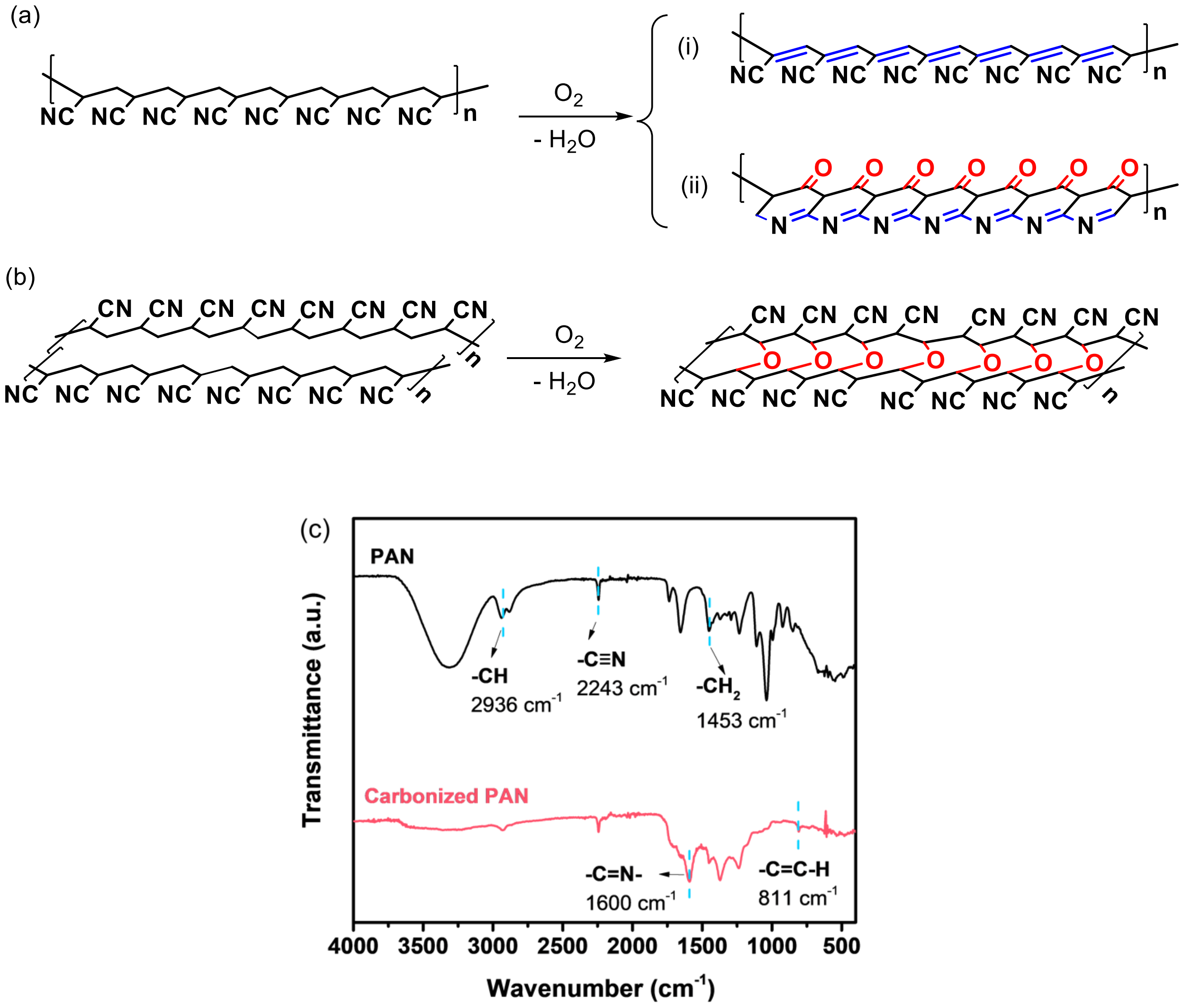

Supplementary Figure 4. Carbonization mechanism of PAN substrates: (a) Intramolecular cyclization; (b) Intermolecular crosslinking. (c) FTIR spectra of PAN and carbonized PAN.

Note: During carbonization, β-C in PAN chains was oxidized to carbonyl, and –C≡N was cyclized to form –C=N[9]. Besides the intramolecular crosslinking reaction, the intermolecular crosslinking reaction might occur between the *β*-C of the two PAN chains that combine with O to form a C–O–C structure, and finally forms a three-dimensional crosslinked network structure with high thermal resistance[10].

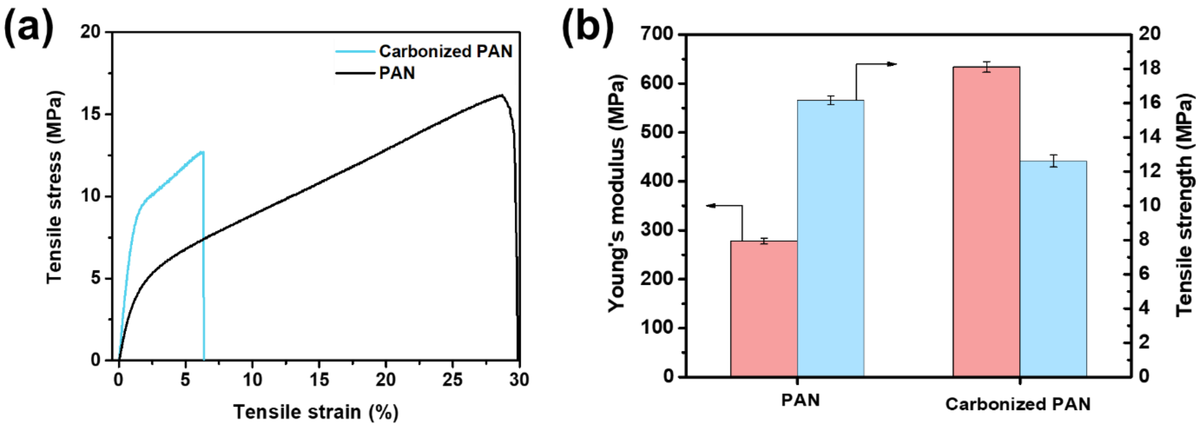

Supplementary Figure 5. (a) Stress-strain curves and (b) Young's modulus and tensile strength of the PAN and carbonized PAN substrates. Error bars represent standard deviations for 3 measurements.

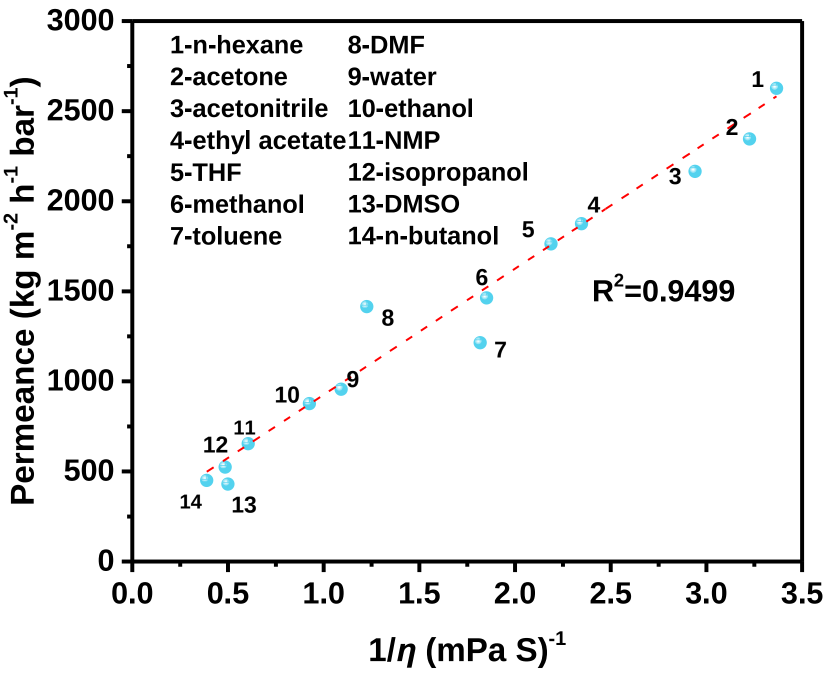

Supplementary Figure 6. Pure solvent permeance as a function of their inverse viscosity for carbonized PAN substrate.

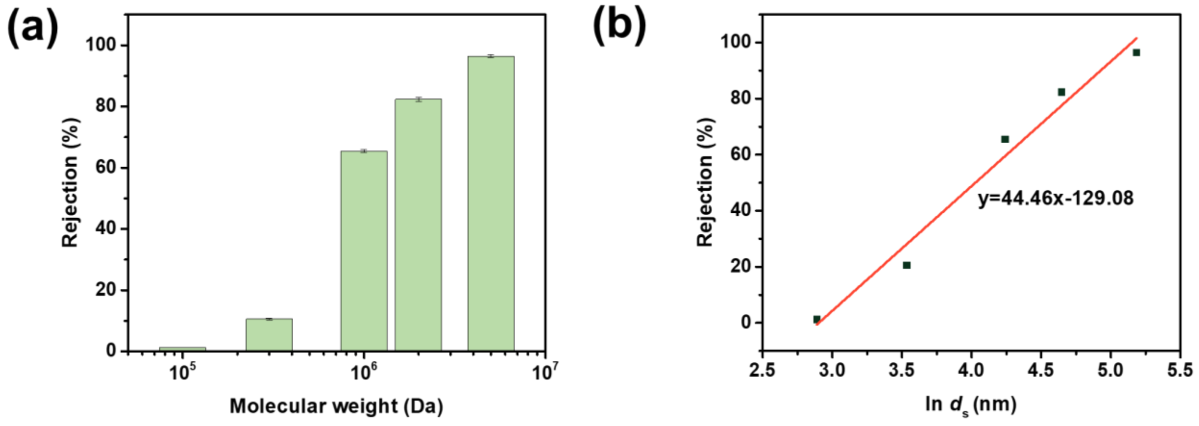

Supplementary Figure 7. (a) Rejection of the carbonized PAN substrates for PEO with different molecular weights. (b) Rejection as a function of ln d_S. Error bars represent standard deviations for 3 measurements.

Note: MWCO of the carbonized PAN substrate was measured by rejecting PEO with different molecular weights. Since rejection is the log-normal probability function of the solute size (d_S), a straight line can be obtained when plotting a log-normal probability curve of the rejection against solute size. The MWCO of the carbonized PAN substrates was calculated to be 1400 kDa.

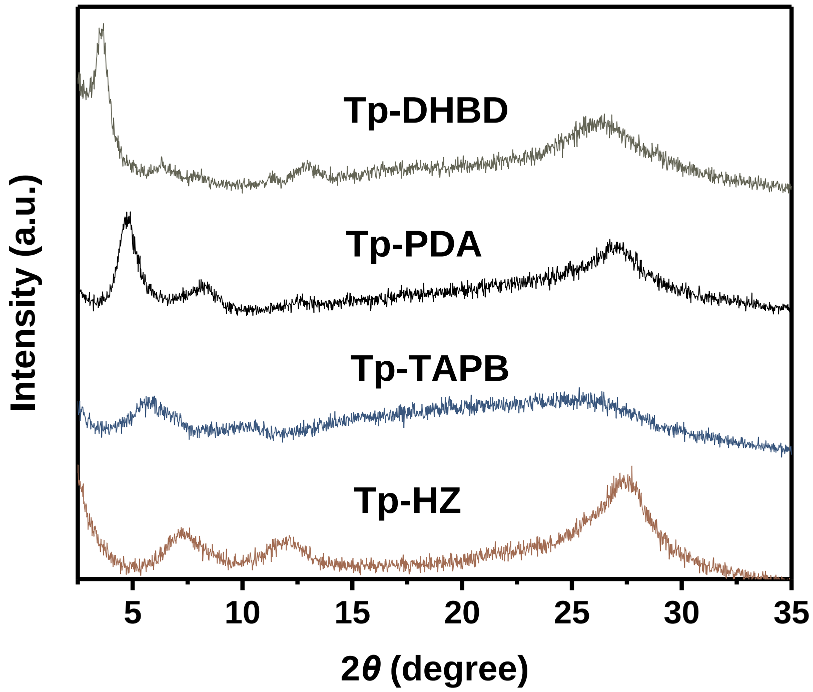

Supplementary Figure 8. XRD patterns of COF Tp-HZ, Tp-TAPB, Tp-PDA, and Tp-DHBD membranes.

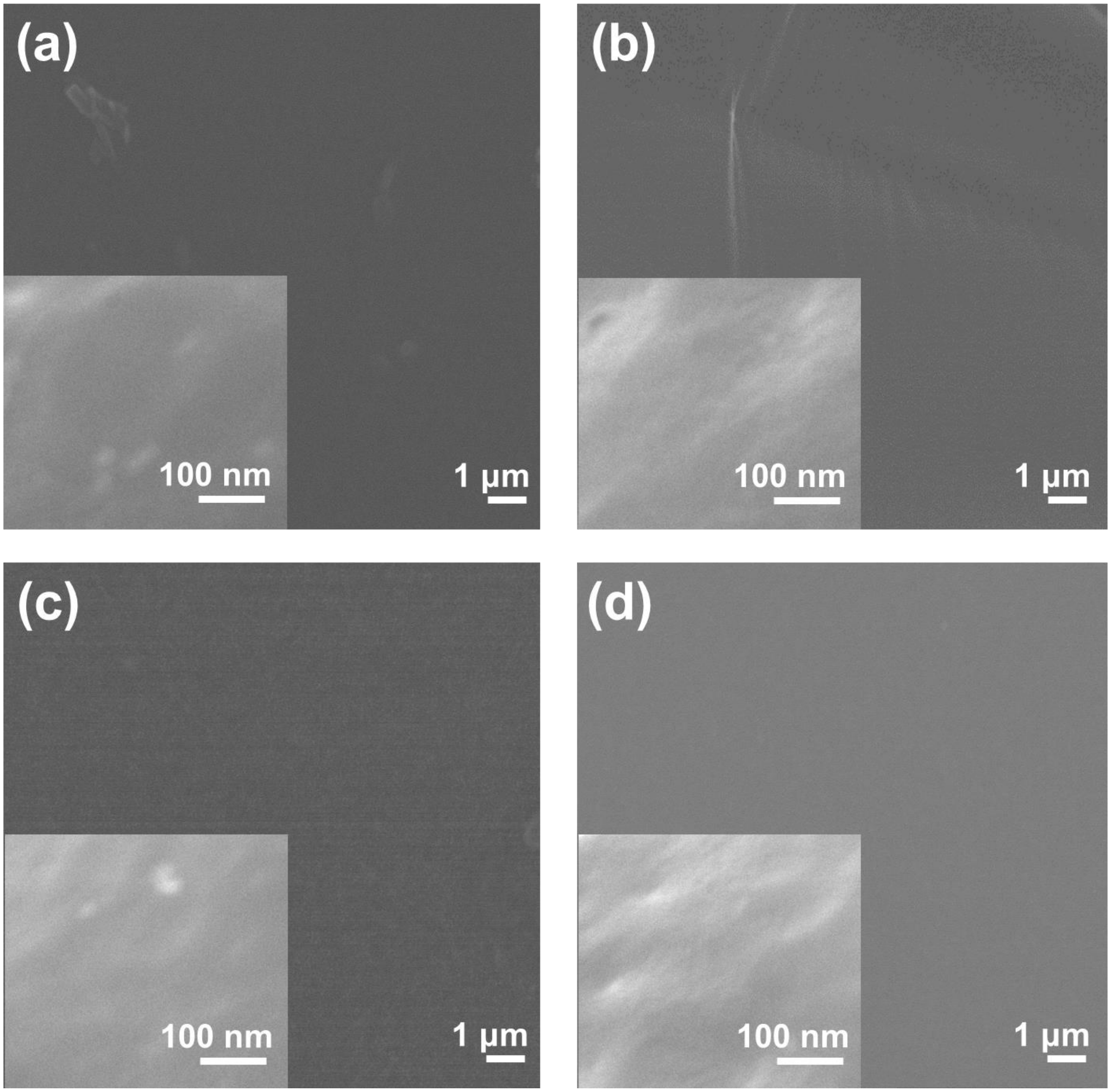

Supplementary Figure 9. Surface FESEM images of (a) Tp-HZ, (b) Tp-TAPB, (c) Tp-PDA, and (d)Tp-DHBD membranes inserted with the corresponding high-resolution FESEM images.

Note: No cracks or defects could be observed from the FESEM images of the COF membranes, indicating their continuous coverage and high quality.

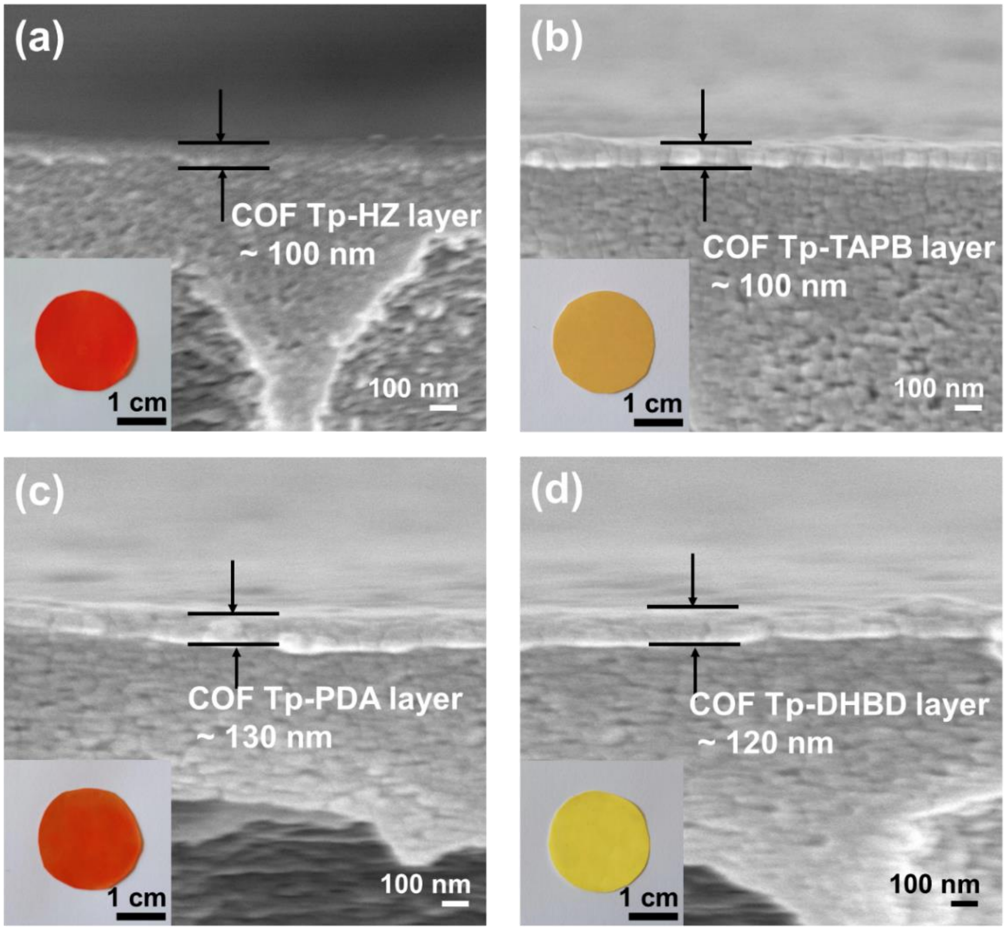

Supplementary Figure 10. Cross-sectional FESEM images of (a)Tp-HZ, (b)Tp-TAPB, (c) Tp-PDA, and (d) Tp-DHBD membranes inserted with the optical photos of the corresponding COF membranes.

Note: The thickness of the selective COF layer in the membranes can be controlled within 100-130 nm via interfacial polymerization.

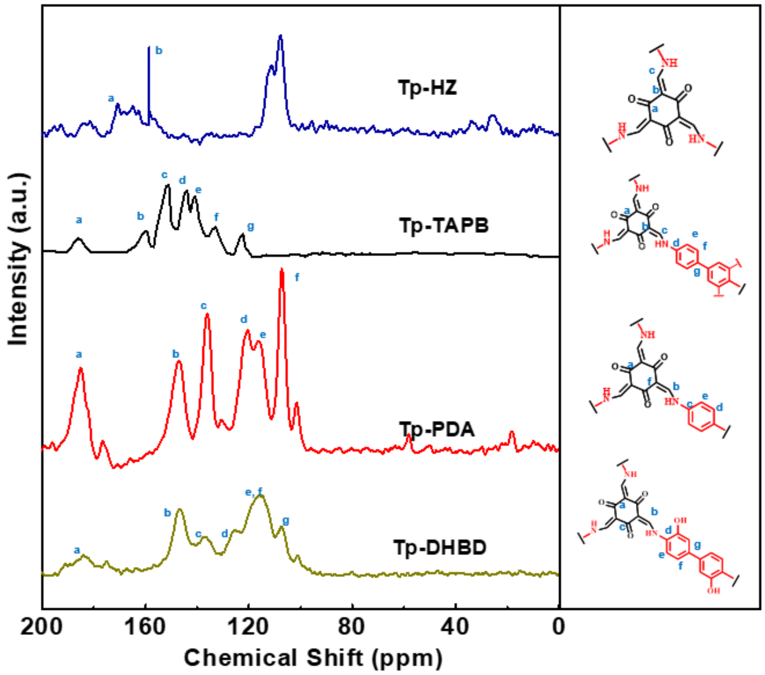

Supplementary Figure 11. Solid-state ^{13}C NMR spectra of the COFs.

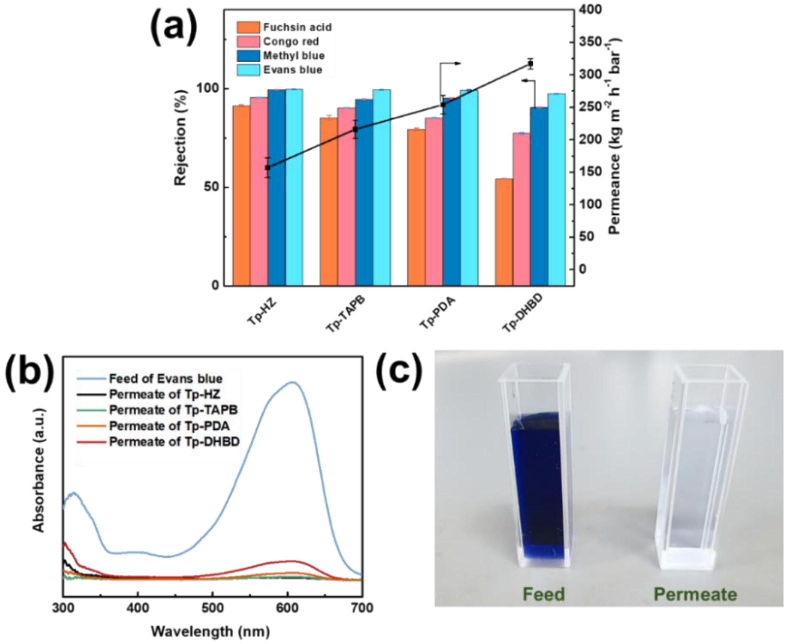

Supplementary Figure 12. (a) Dye rejection performance of the COF membranes. (b) UV-Vis absorption spectra of Evans blue in feed and permeate. (c) Photo of feed (left, 100 ppm of Evans blue) and permeate (right). Error bars represent standard deviations for 3 measurements.

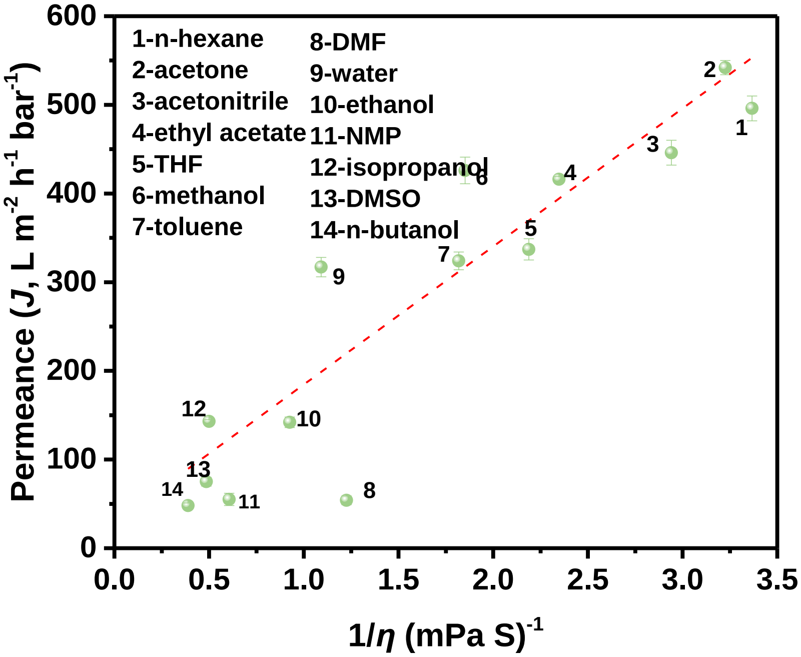

Supplementary Figure 13. Pure solvent permeance as a function of their inverse viscosity for COF Tp-TAPB membrane. Error bars represent standard deviations for 3 measurements.

Note: Used solvents are numbered and named on the left. Red dashed line indicates the hypothetical linear relationship.

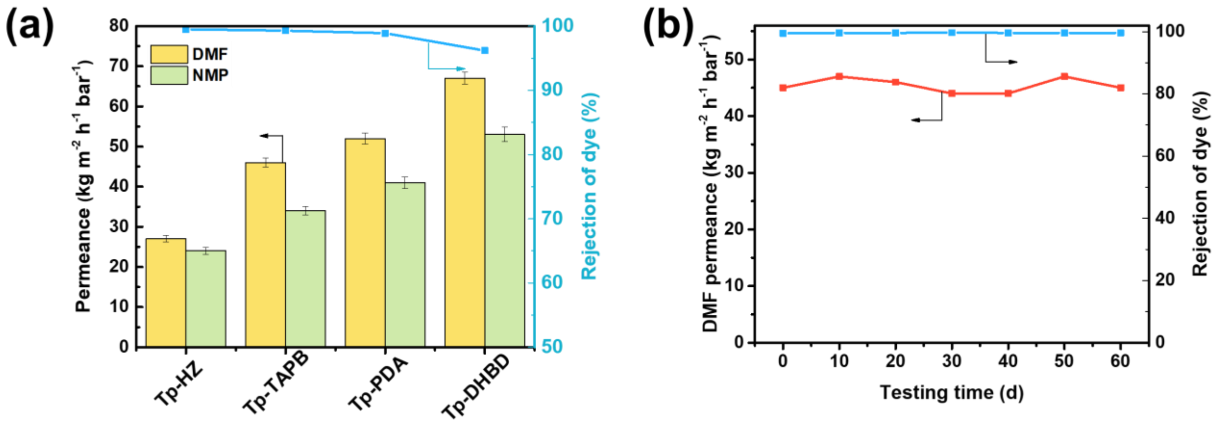

Supplementary Figure 14. (a) Dye rejection performance of the COF membranes in aggressive organic solvent nanofiltration. (b) Long-term test of DMF permeance and dye rejection of Tp-TAPB membrane. Error bars represent standard deviations for 3 measurements.

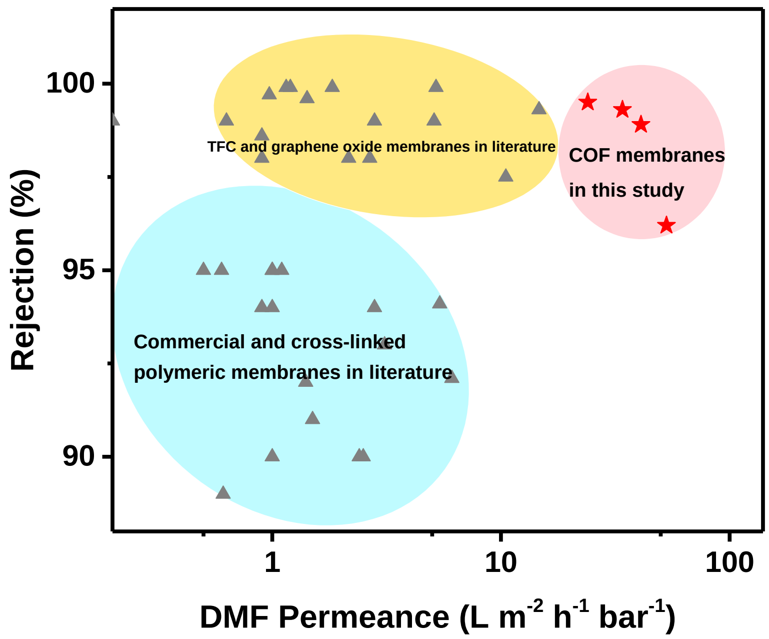

Supplementary Figure 15. Nanofiltration performance comparison of commercial polymeric membranes, state-of-the-art membranes, and our COF membranes for dye rejection in DMF[11-22].

Supplementary Table 1. A list of optimal COF membranes for recovering each kind of photocatalyst.

Photocatalyst	Size	Optimal COF membrane
$[Ir\text{-}1]PF_6$	1.50nm × 1.38nm × 1.34nm	Tp-TAPB
$[Ir\text{-}2]PF_6$	1.60nm × 1.36nm × 1.29nm	Tp-TAPB
$[Ir\text{-}3]PF_6$	1.51nm × 1.20nm × 1.15nm	Tp-TAPB
$[Ru](PF_6)_2$	1.92nm × 1.12nm × 1.10nm	Tp-HZ
NaDT	1.57nm × 1.48nm × 1.10nm	Tp-DHBD
(*R*)-TRIP	1.90nm × 1.39nm × 1.11nm	Tp-TAPB

Note: The size of photocatalysts was calculated by Multiwfn programme[23] using their corresponding crystallographic data. The crystallographic data (x.cif file) were obtained from the Cambridge Crystallographic Data Centre (CCDC): **$[Ir\text{-}1]PF_6$** (CCDC number: 863359, a very similar structure with a difference from $-CF_3$ group to $-CH_3$ group), **$[Ir\text{-}2]PF_6$** (CCDC number: 1490702), **$[Ir\text{-}3]PF_6$** (CCDC number: 2076270), **$[Ru](PF_6)_2$** (CCDC number: 101676), **(*R*)-TRIP** (CCDC number: 818066), **NaDT** (CCDC number: 2189883). The 3D structures of the photocatalysts were constructed using VMD software.

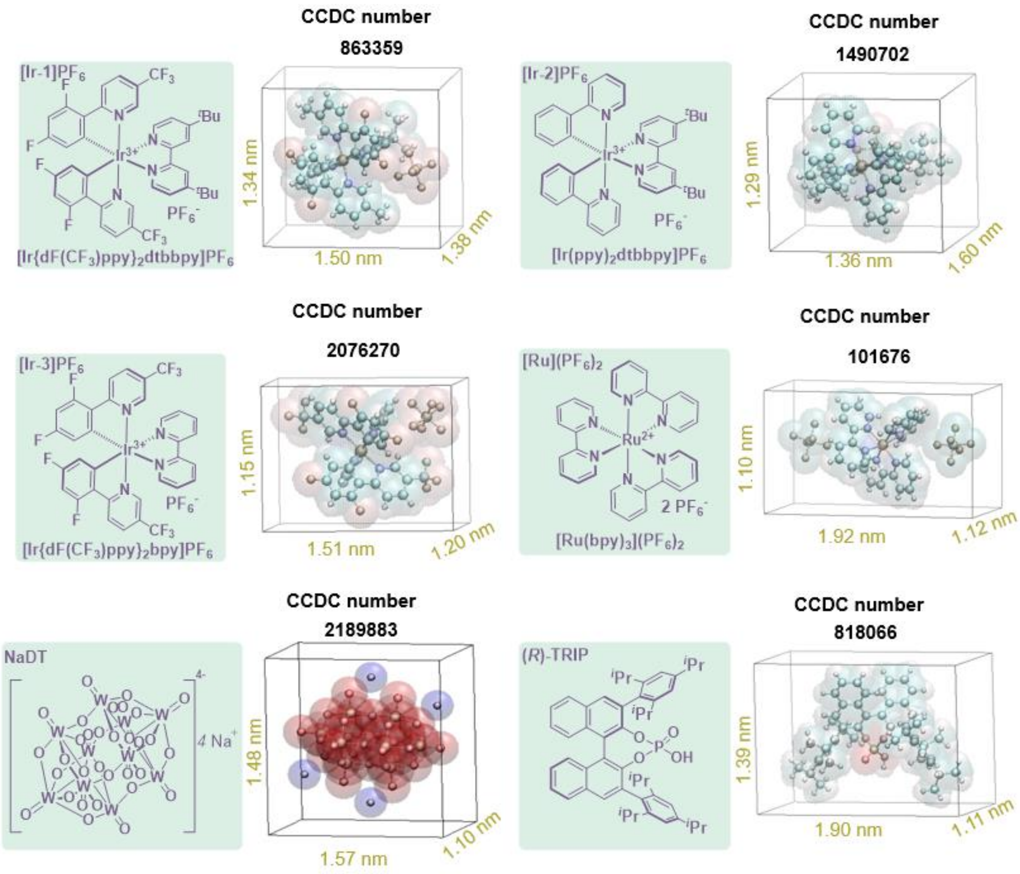

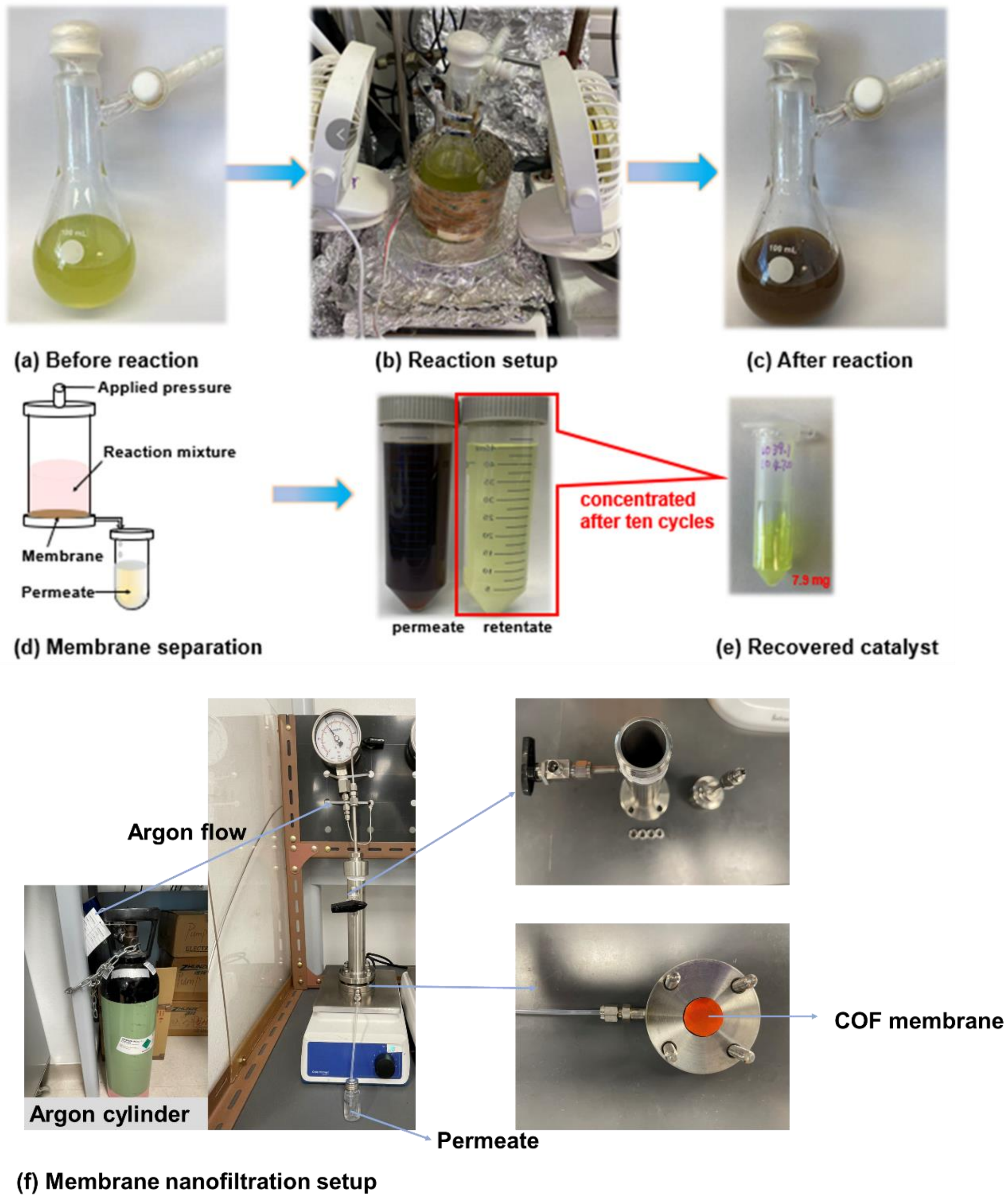

Supplementary Figure 16. Procedure for aryl amination reaction and recovery of the photocatalyst. (a-c) Photos of the photocatalysis reaction. (d) Schematic of membrane separation and photos of permeate and retentate. (e) Photo of the recovered catalyst. (f) Membrane nanofiltration setup operated under argon for photocatalyst recovery.

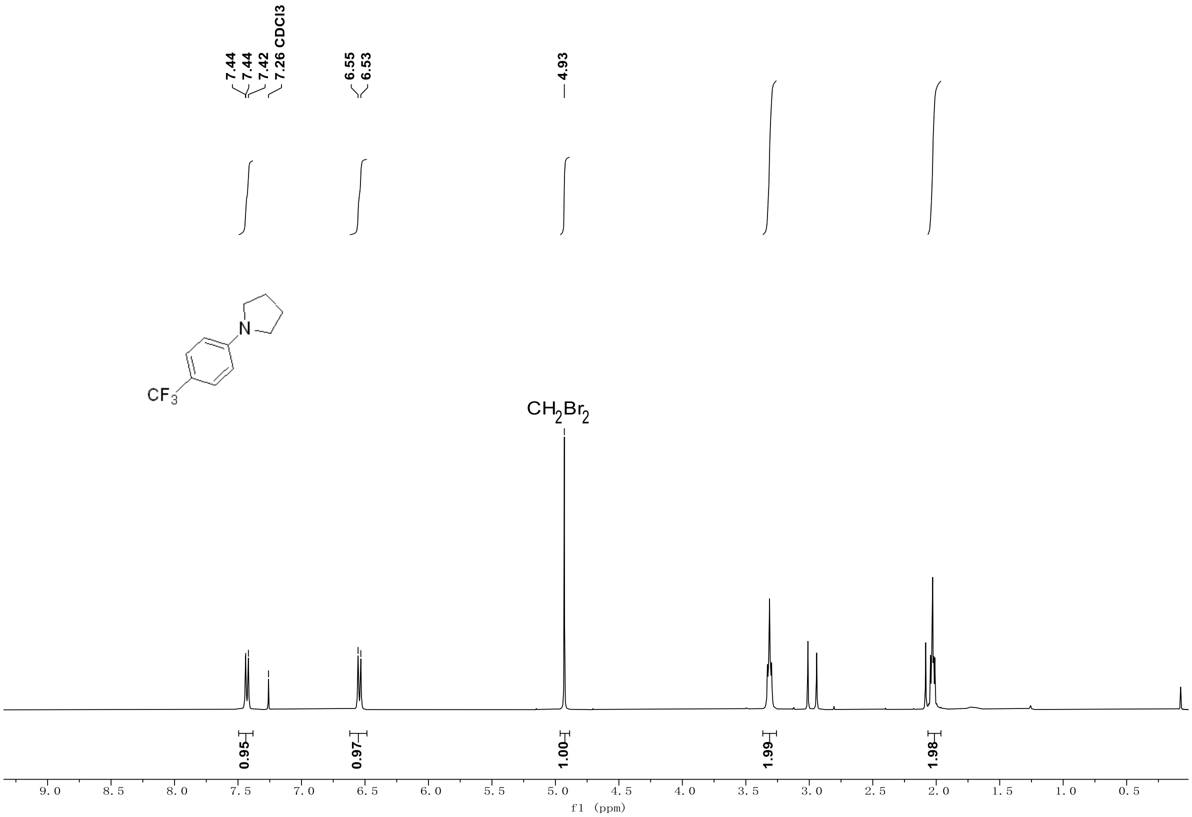

Supplementary Figure 17. Typical crude ^{1}H NMR of the aryl amination reaction using CH_2Br_2 as an internal standard.

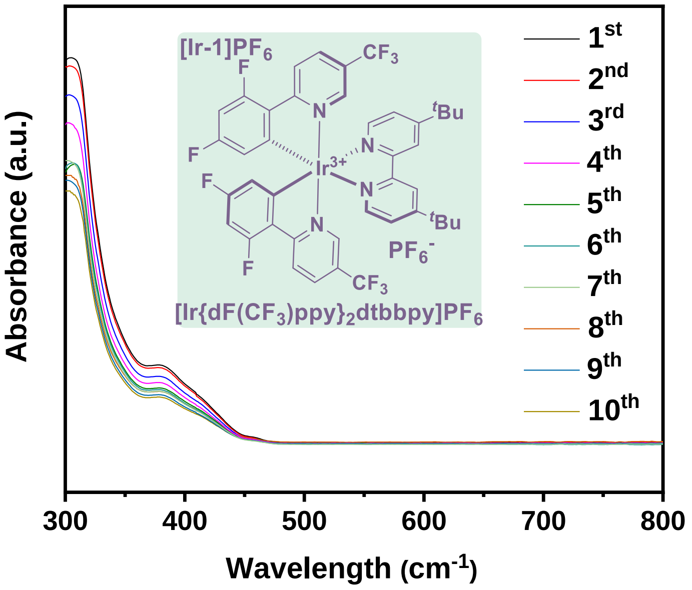

Supplementary Figure 18. UV-Vis spectra of the recovered catalyst for aryl amination reactions in ten cycles.

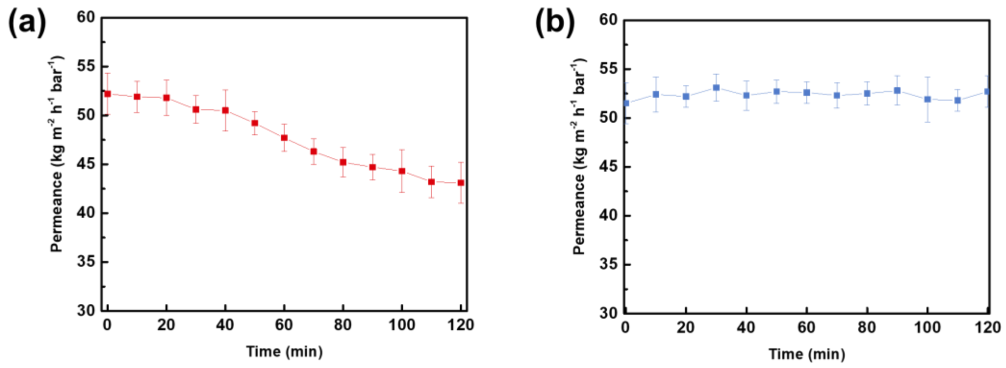

Supplementary Figure 19. (a) Permeance of catalyst recovery for the aryl amination reaction as a function of time. (b) Permeance of COF membrane with a feed of constant catalyst concentration. Error bars represent standard deviations for 3 measurements.

Note: Permeance drops with increasing filtration time due to the concentration polarization (**Supplementary Fig. 19a**). When keeping catalyst concentration constant by adding back pure solvent every 10 min, the permeance of COF membrane was kept steady (**Supplementary Fig. 19b**). Considering that the initial permeance reflects the permeance of COF membranes for separating the reaction mixtures with specific concentrations, all of the initial permeance was used in **Figure 3**.

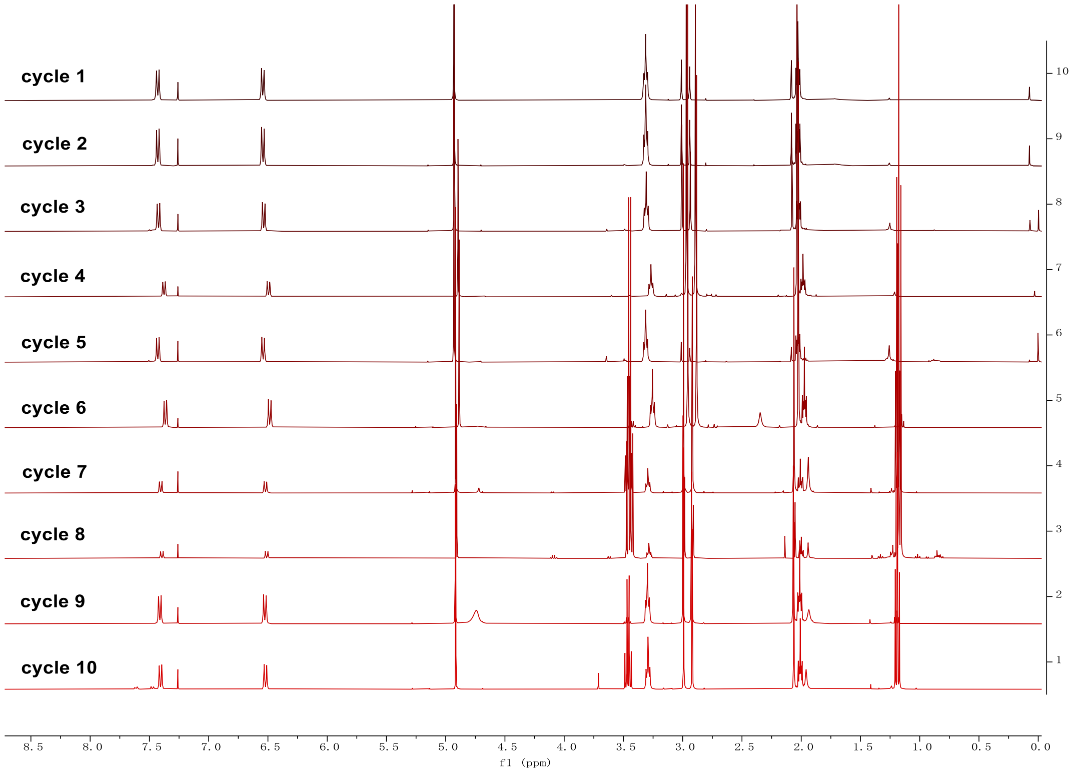

Supplementary Figure 20. Crude ^{1}H NMR spectra of 10 cycles of the aryl amination reaction using CH_2Br_2 as an internal standard.

Supplementary Table 2. Yields and catalyst recovery rates of the aryl amination reaction in ten cycles.

Cycle	1	2	3	4	5	6	7	8	9	10
Yield (%)	98	98	94	99	92	93	95	98	95	92
[Ir-1]PF_6 (mg)	14.2	13.8	13.3	12.3	11.3	10.4	9.8	9.4	9.0	8.7
Catalyst recovery (%)	90.6	97.3	95.0	94.3	92.8	92.0	94.2	95.9	95.7	96.7

Note: As shown in **Supplementary Fig. 20** and **Supplementary Table 2**, the reactions in 10 cycles maintained steady yields and high catalytic activity, indicating that the effective recovery and reuse of photocatalysts from the aryl amination reaction have been achieved by COF membranes.

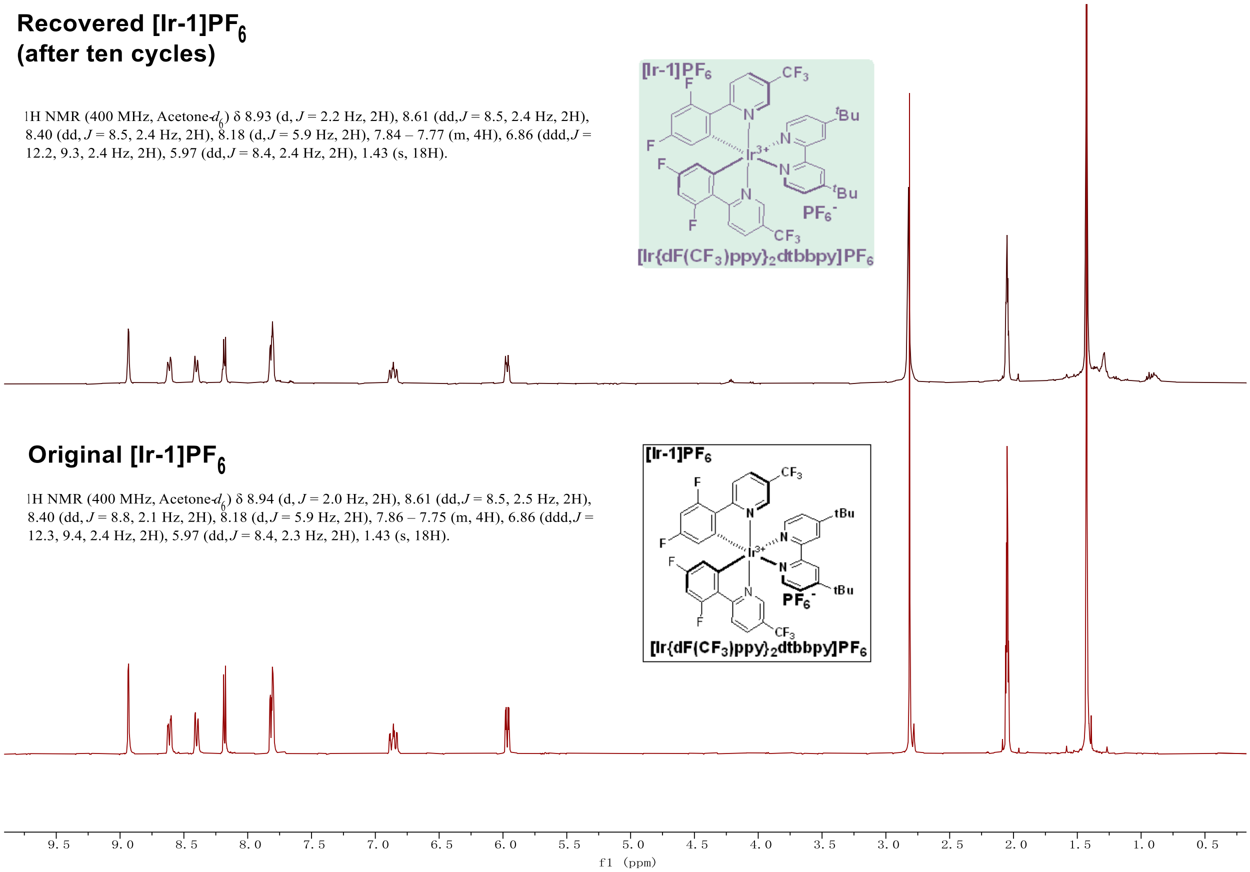

Supplementary Figure 21. ^{1}H NMR spectra of original and final recovered photocatalyst [Ir-1]PF_6 for the aryl amination reaction.

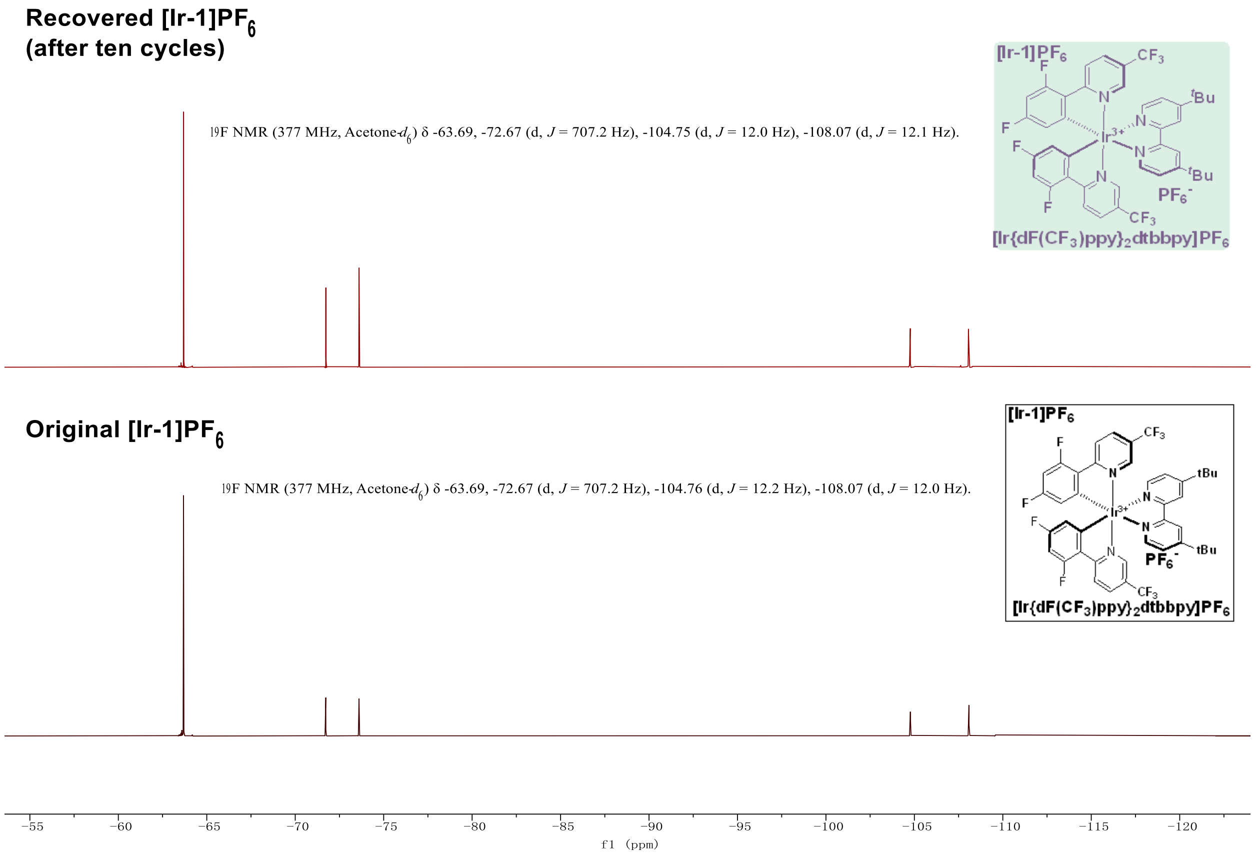

Supplementary Figure 22. ^{19}F NMR spectra of original and final recovered photocatalyst [Ir-1]PF_6 for the aryl amination reaction.

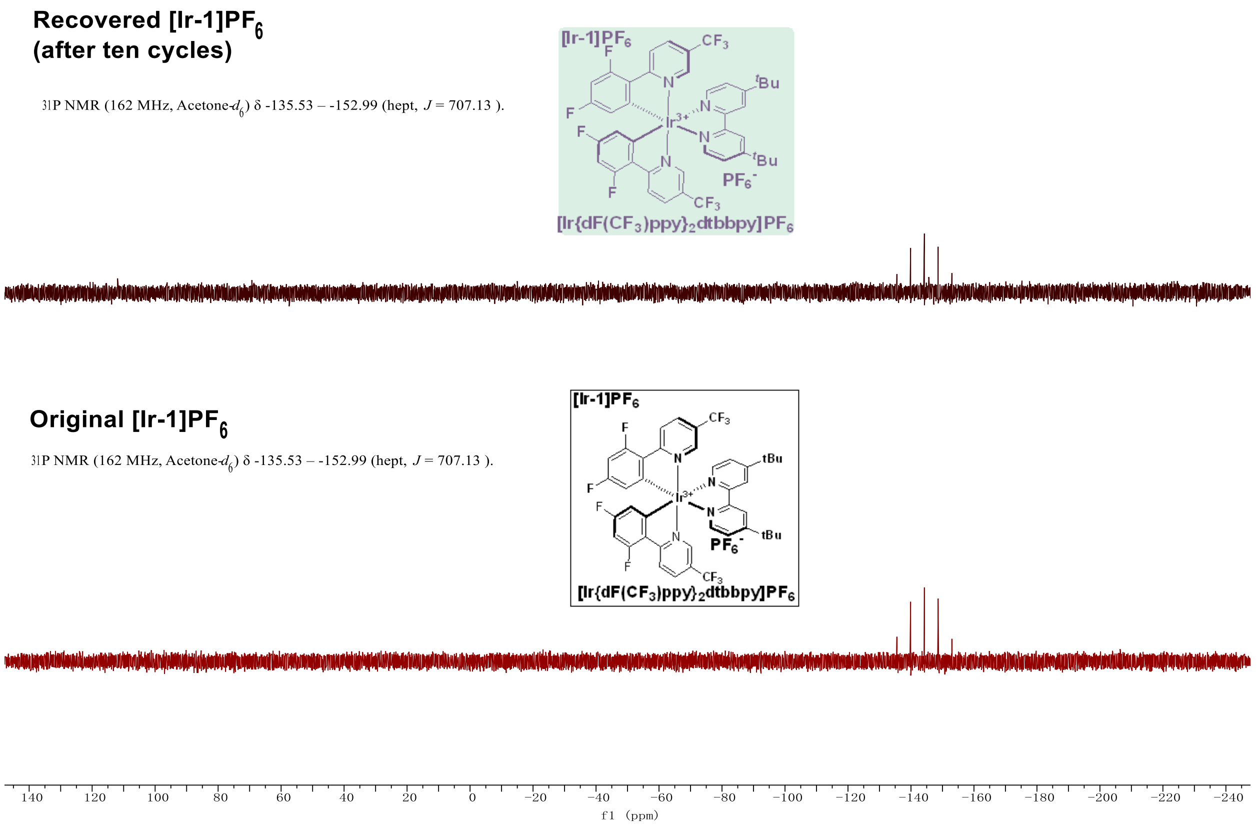

Supplementary Figure 23. ^{31}P NMR spectra of original and final recovered photocatalyst [Ir-1]PF_6 for the aryl amination reaction.

Note: After the final cycle, the recovered catalyst **[Ir-1]PF_6** was dried under vacuum and analyzed by NMR in acetone-*d*6. The commercial photocatalyst **[Ir-1]PF_6** was used as the original one to better identify the recovered photocatalyst. As shown in **Supplementary Figs. 21-23**, all the spectra, including ^{1}H, ^{19}F, and ^{31}P NMR of recovered catalyst **[Ir-1]PF_6**, are consistent with the original ones, indicating the high purity of the recovered noble metal catalyst with no degradation.

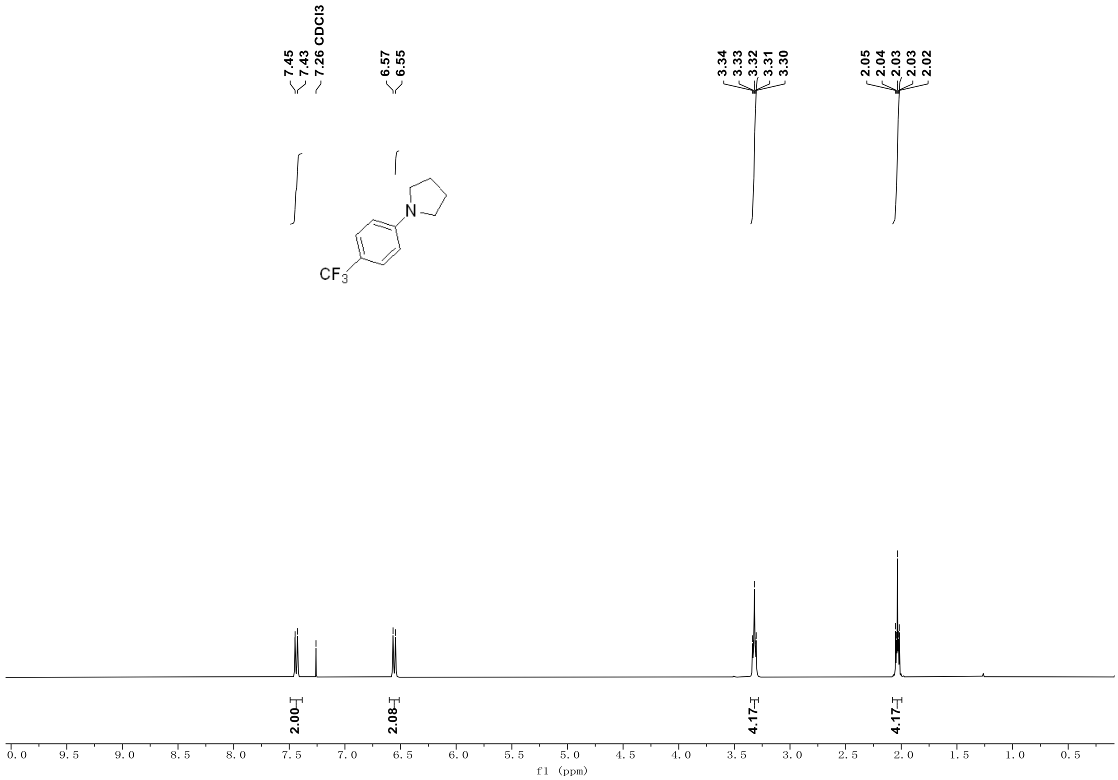

Supplementary Figure 24. ^{1}H NMR spectrum of 1-(4-(trifluoromethyl)phenyl)pyrrolidine.

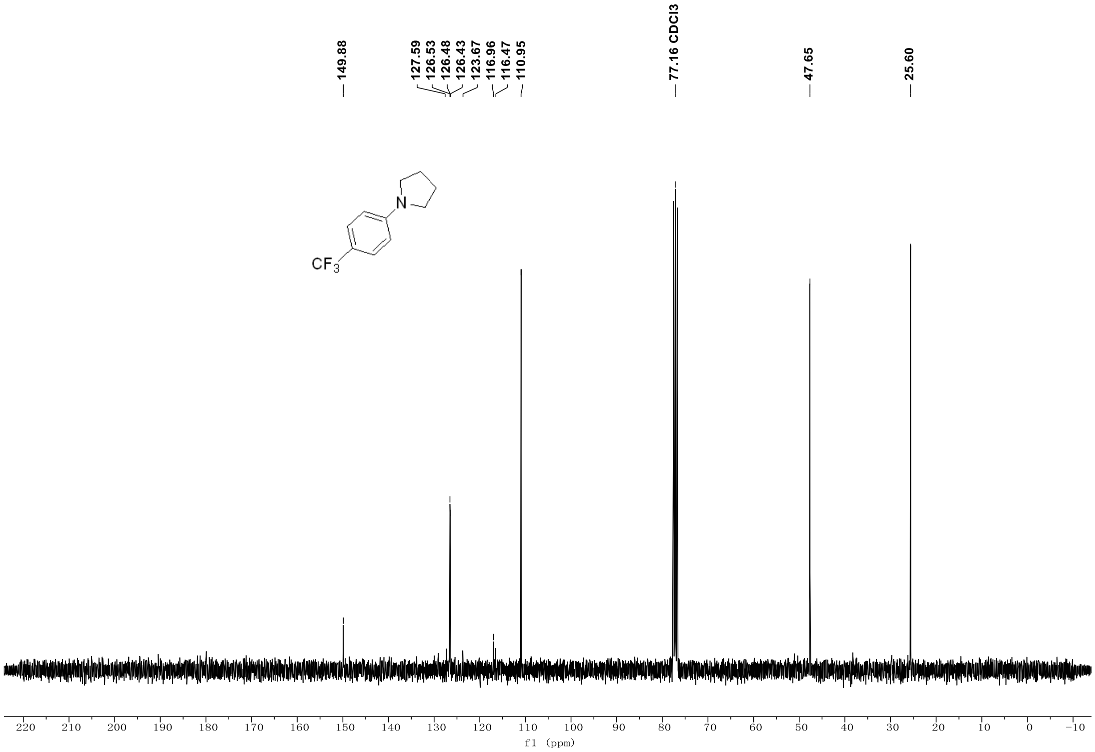

Supplementary Figure 25. ^{13}C NMR spectrum of 1-(4-(trifluoromethyl)phenyl)pyrrolidine.

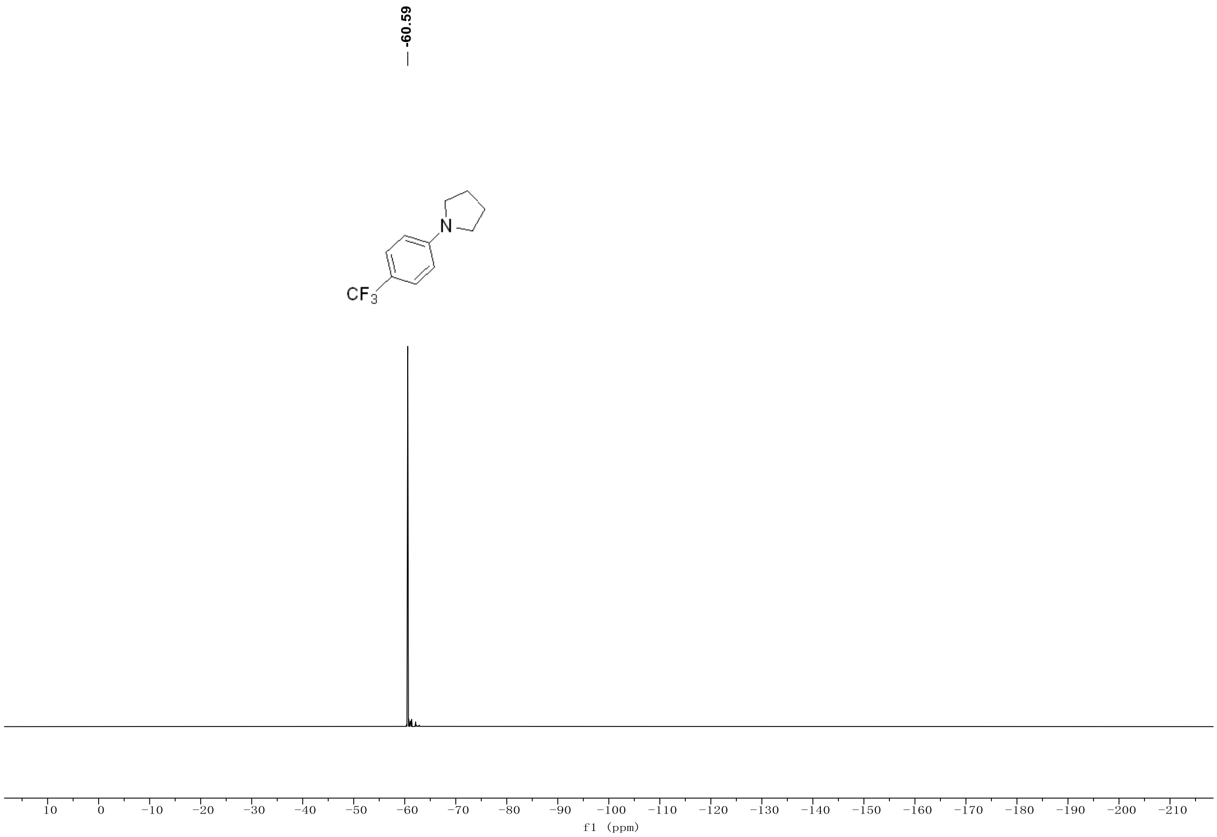

Supplementary Figure 26. ^{19}F NMR spectrum of 1-(4-(trifluoromethyl)phenyl)pyrrolidine.

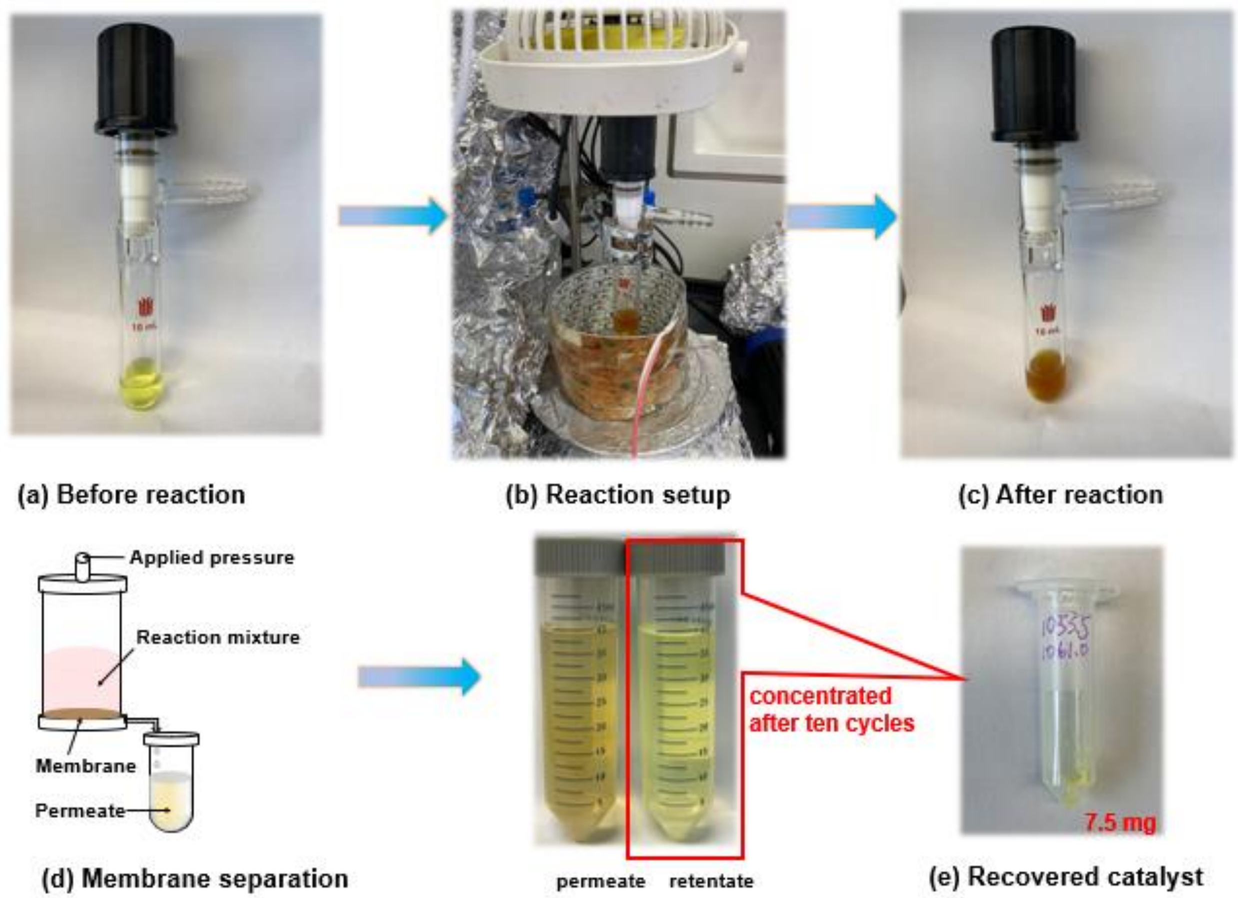

Supplementary Figure 27. Procedure for the intermolecular cycloaddition via EnT and recovery of the photocatalyst. (a-c) Photos of the photocatalysis reaction process. (d) Schematic of membrane separation and photos of permeate and retentate. (e) Photo of the recovered catalyst.

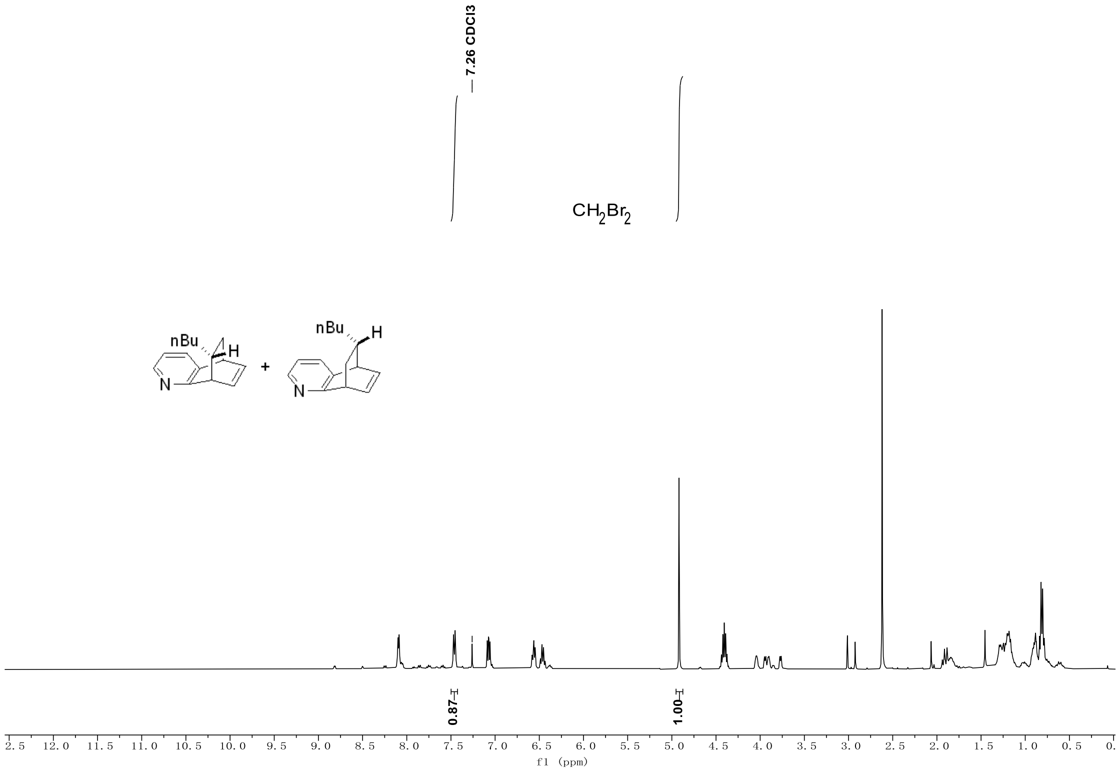

Supplementary Figure 28. Typical crude ^{1}H NMR spectrum of the intermolecular cycloaddition via EnT using CH_2Br_2 as an internal standard.

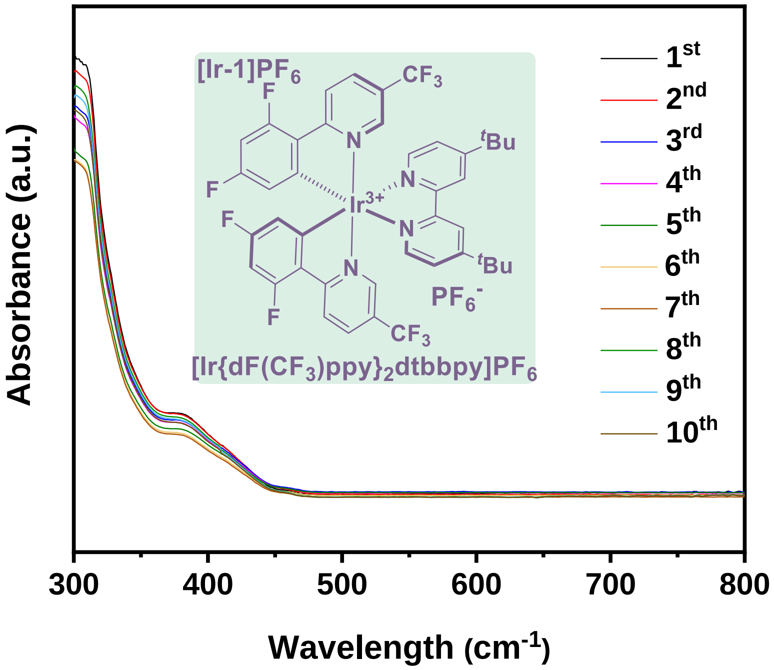

Supplementary Figure 29. UV-Vis spectra of the recovered catalyst for intermolecular cycloaddition via EnT in 10 cycles.

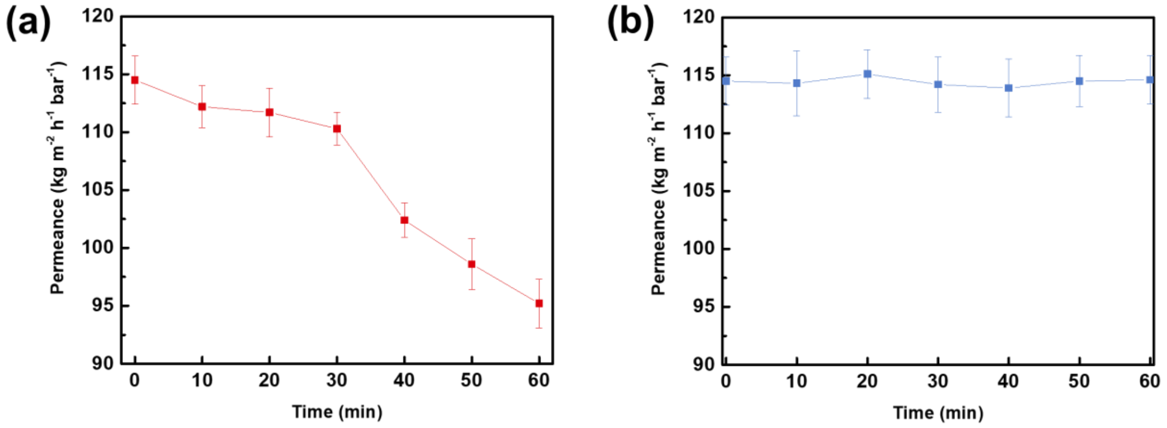

Supplementary Figure 30. (a) Permeance of catalyst recovery for intermolecular cycloaddition via EnT as a function of time. (b) Permeance of COF membrane with a feed of constant catalyst concentration. Error bars represent standard deviations for 3 measurements.

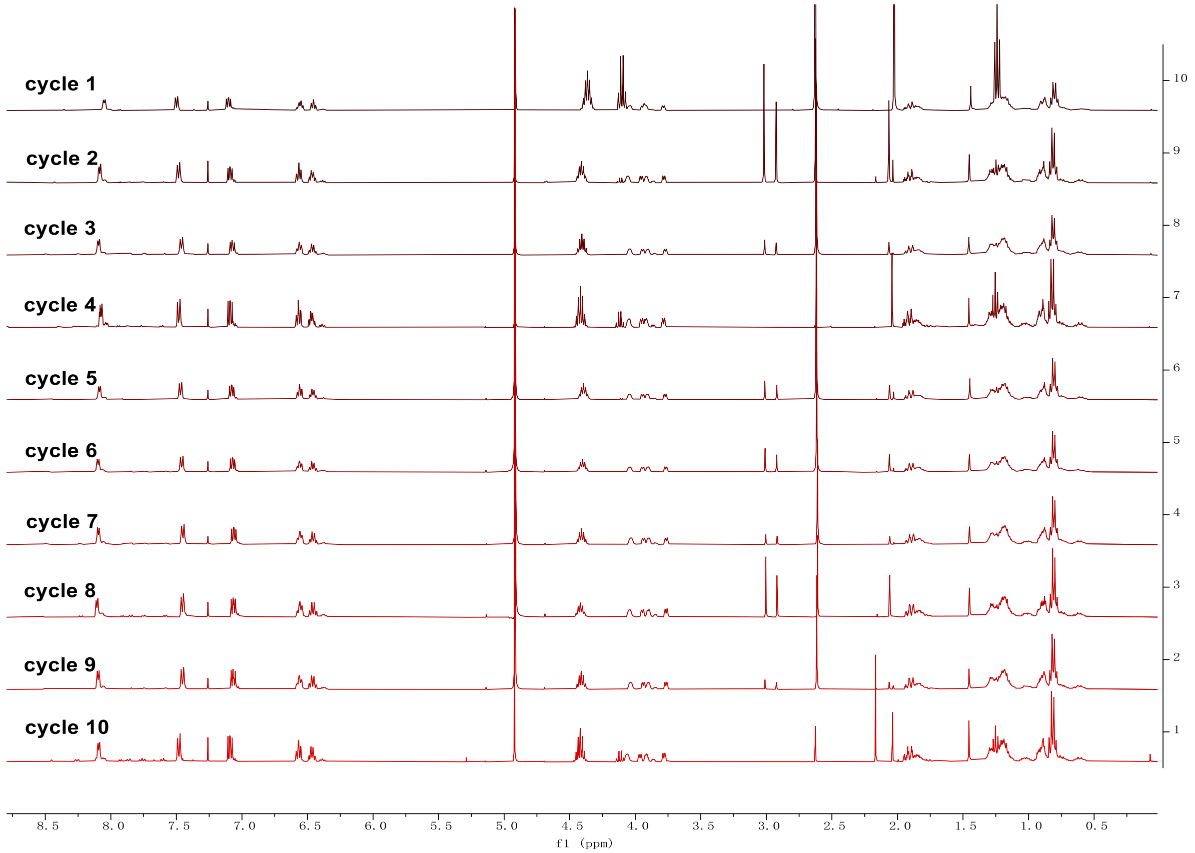

Supplementary Figure 31. Crude ^{1}H NMR spectra of ten cycles for the intermolecular cycloaddition via EnT using CH_2Br_2 as an internal standard.

Supplementary Table 3. Yields and catalyst recovery rates of intermolecular cycloaddition via EnT in ten cycles.

Cycle	1	2	3	4	5	6	7	8	9	10
Yield (%)	88	85	87	91	90	87	85	80	85	87
[Ir-1]PF_6 (mg)	12.8	12.5	11.4	10.8	9.9	9.6	9.4	9.1	8.8	8.4
Catalyst recovery (%)	97.4	97.2	91.0	95.2	91.3	96.4	98.2	97.0	96.8	95.8

Note: As shown in **Supplementary Fig. 31** and **Supplementary Table 3**, the reactions in 10 cycles maintained steady yields and high catalytic activity, which indicates that the effective recovery and reuse of photocatalysts from the intermolecular cycloaddition reaction have been achieved by COF membranes.

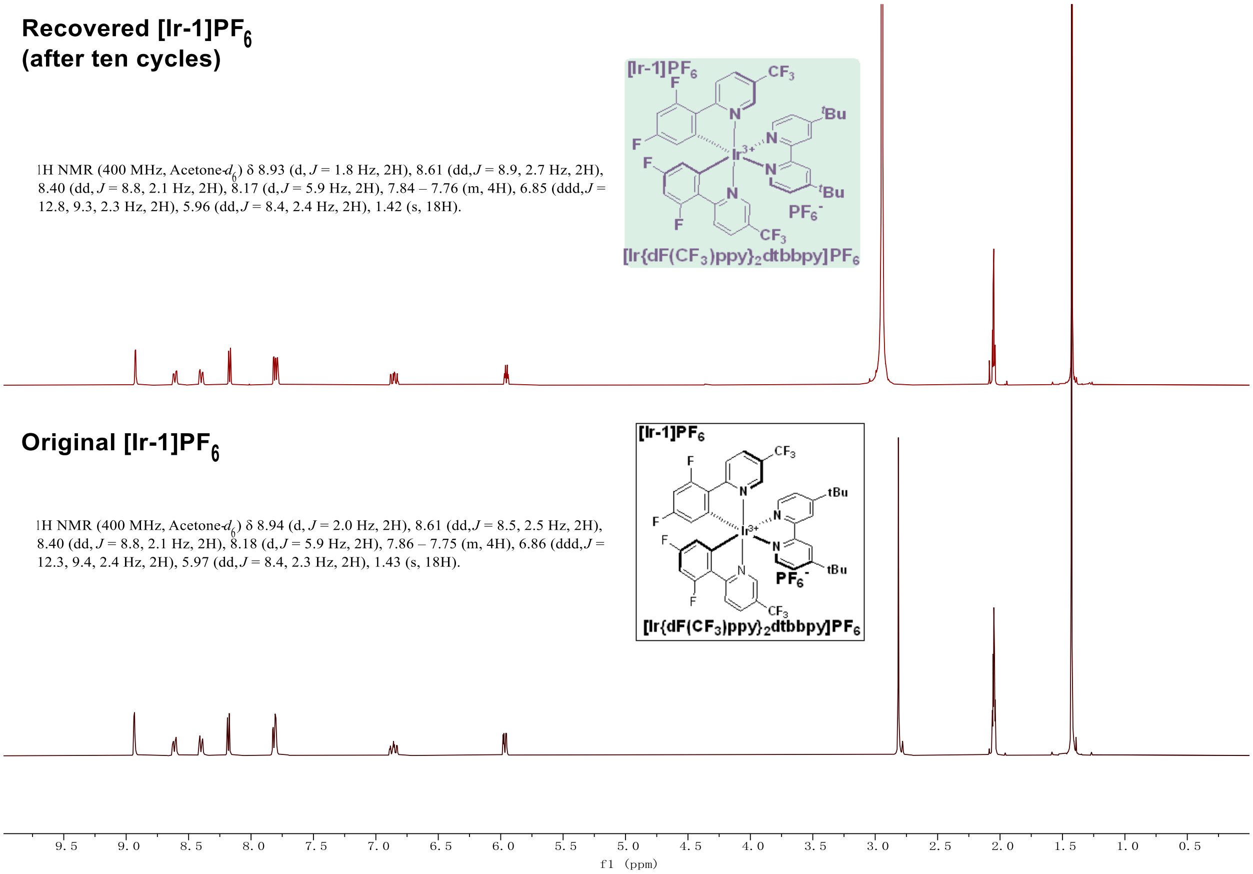

Supplementary Figure 32. ^{1}H NMR spectra of original and recovered photocatalyst [Ir-1]PF_6 for the intermolecular cycloaddition via EnT.

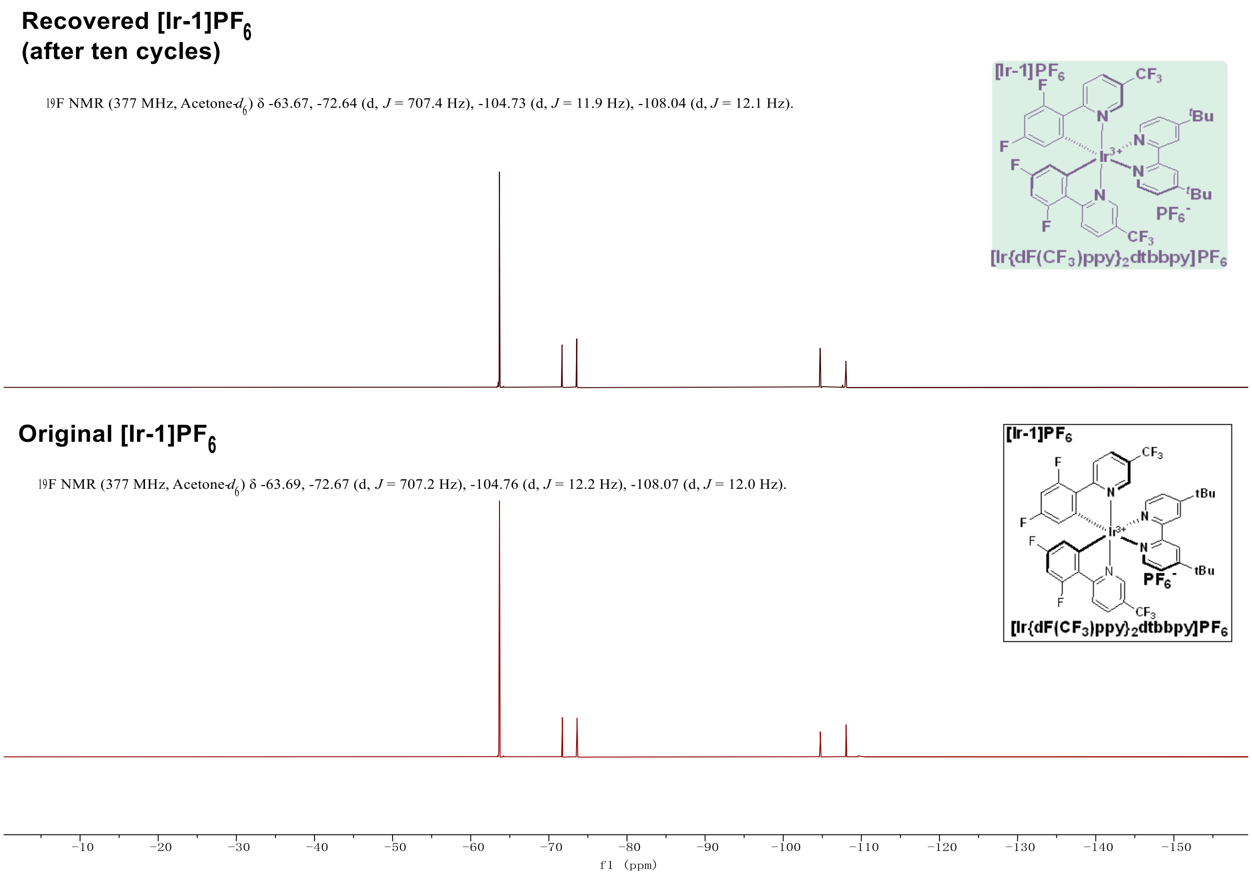

Supplementary Figure 33. ^{19}F NMR spectra of original and recovered photocatalyst [Ir-1]PF_6 for the intermolecular cycloaddition via EnT.

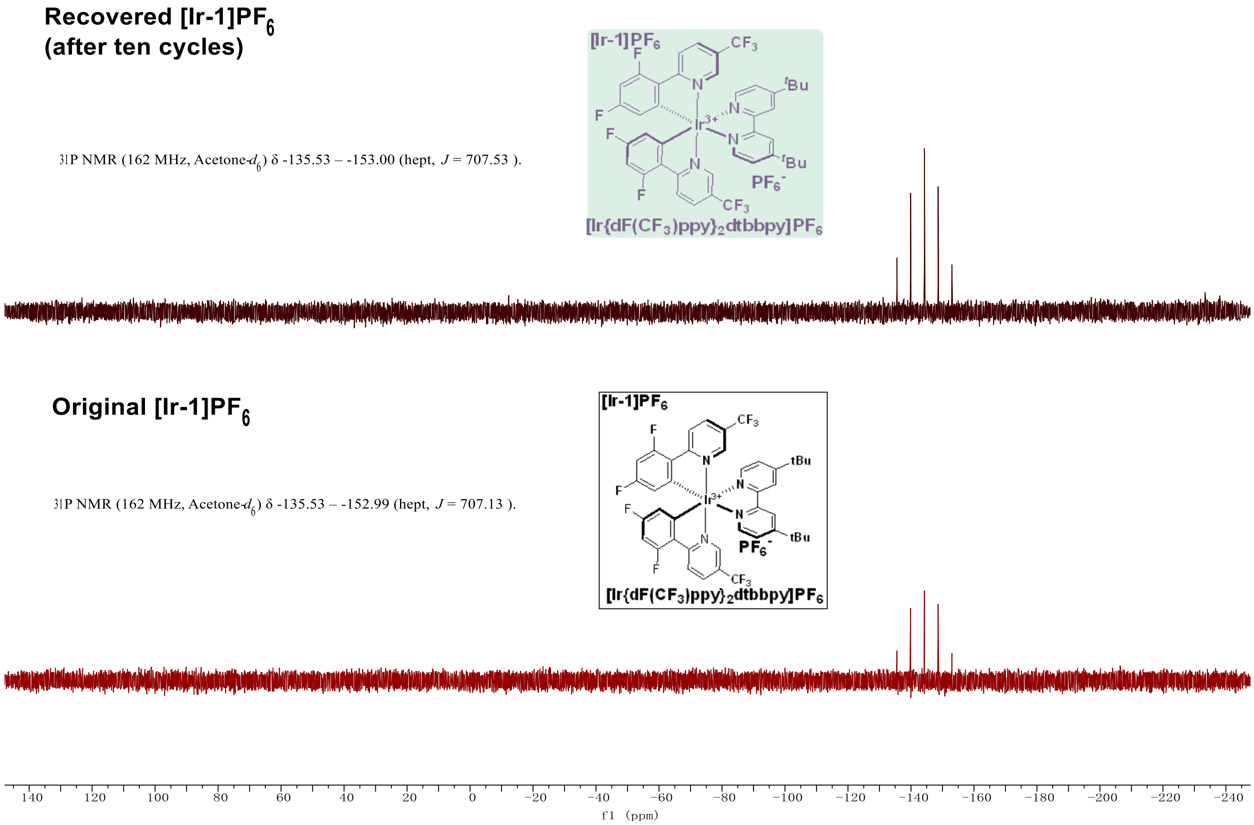

Supplementary Figure 34. ^{31}P NMR spectra of original and recovered photocatalyst [Ir-1]PF_6 for intermolecular cycloaddition via EnT.

Note: After the final cycle, the recovered catalyst **[Ir-1]PF_6** was dried under vacuum and analyzed by NMR in acetone-*d_6*. The purchased photocatalyst **[Ir-1]PF_6** was used as the original one to better identify the recovered photocatalysts. As shown in **Supplementary Figs. 32-34**, all the spectra, including ^{1}H, ^{19}F, and ^{31}P NMR of the recovered catalyst **[Ir-1]PF_6**, are consistent with the original ones, indicating high purity of this recovered noble metal catalyst with no degradation.

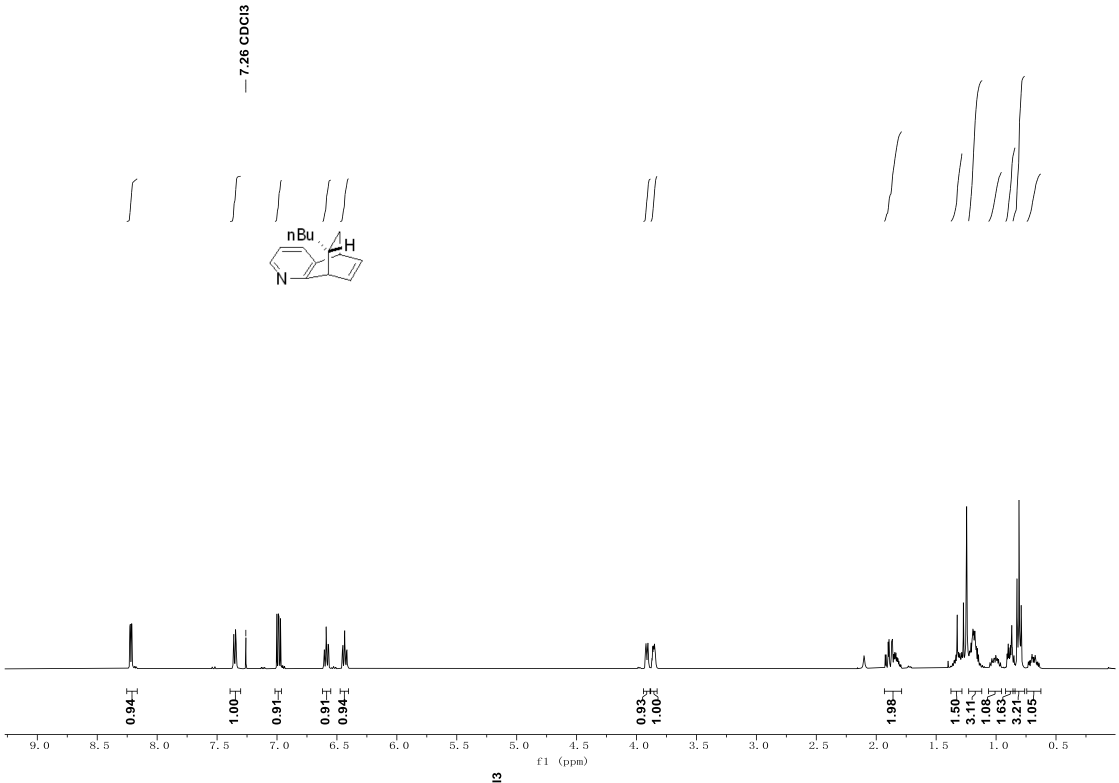

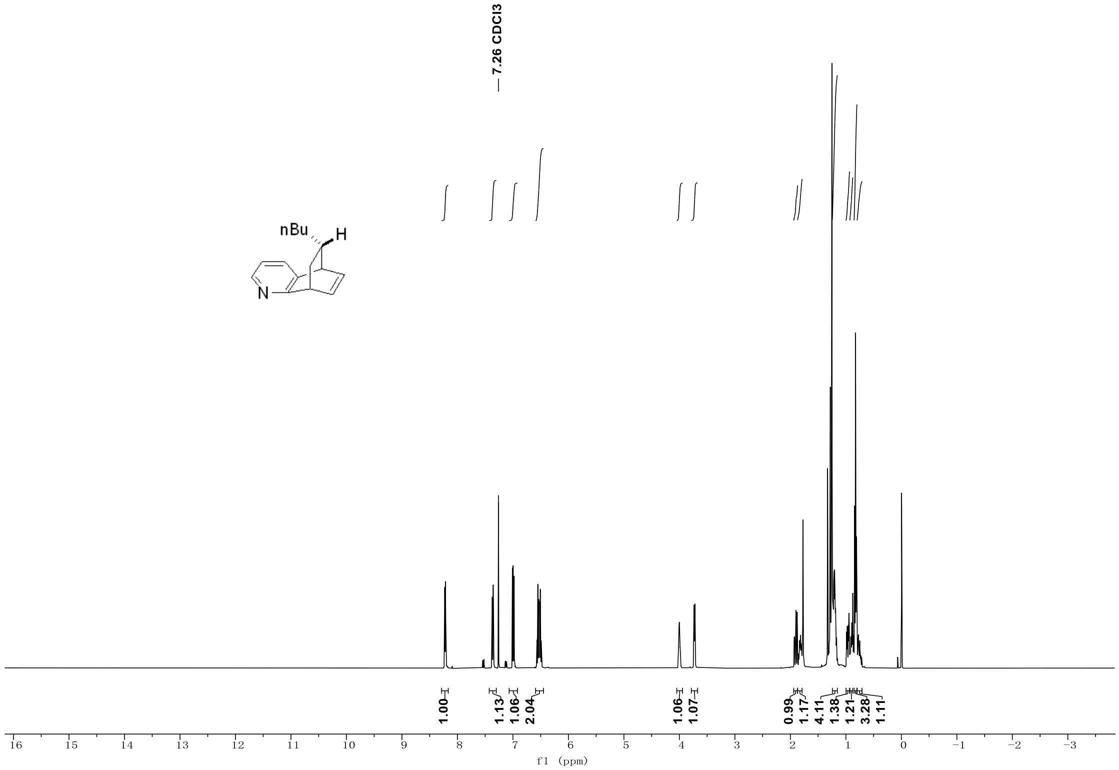

Supplementary Figure 35. ^{1}H NMR spectra of 9-butyl-5,8-dihydro-5,8-ethanoquinoline (top) and 10-butyl-5,8-dihydro-5,8-ethanoquinoline (bottom).

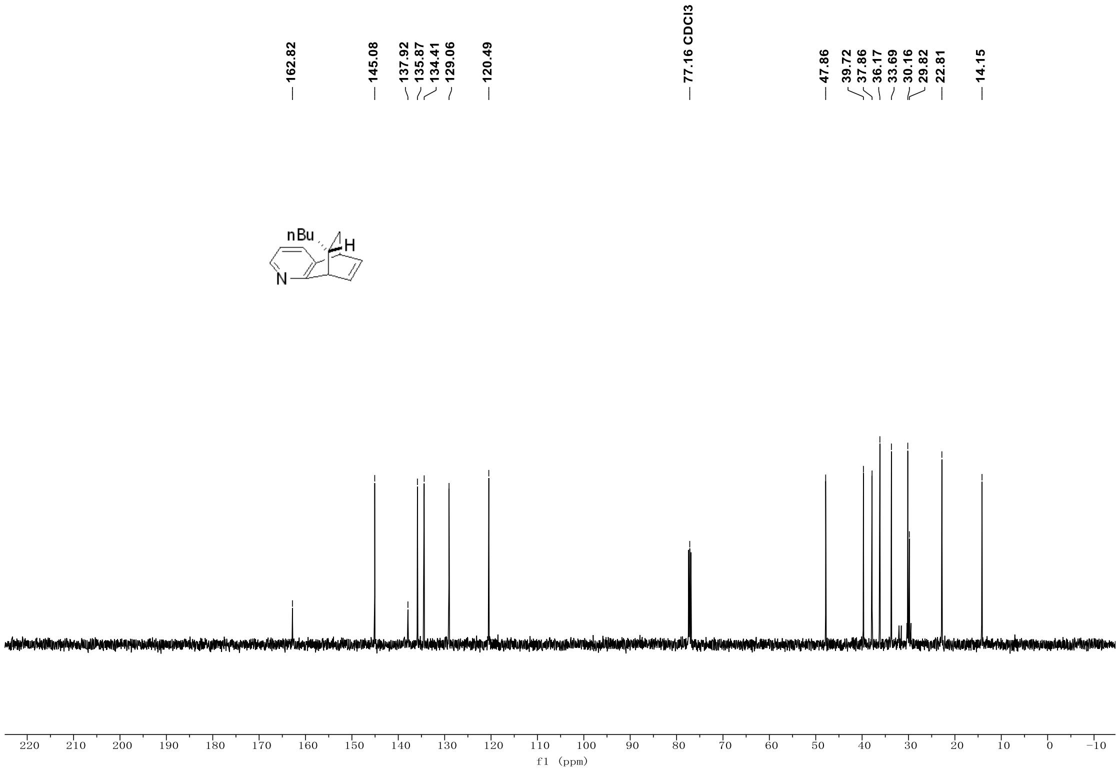

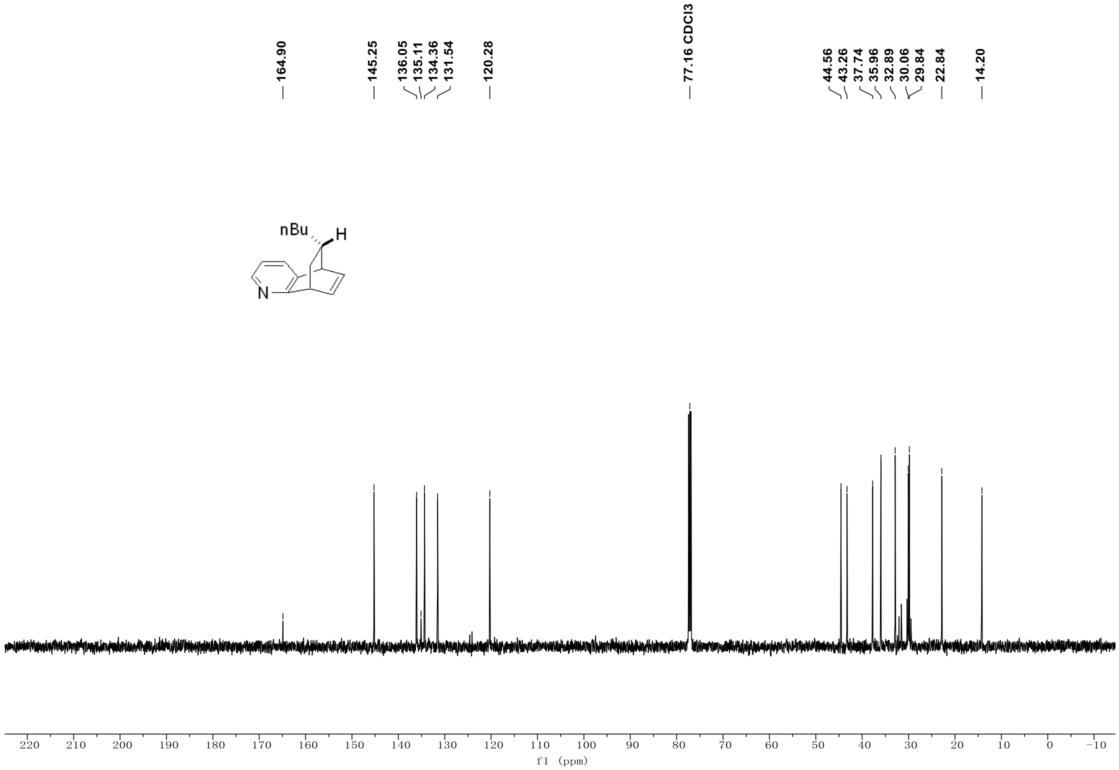

Supplementary Figure 36. ^{13}C NMR spectra of 9-butyl-5,8-dihydro-5,8-ethanoquinoline (top) and 10-butyl-5,8-dihydro-5,8-ethanoquinoline (bottom).

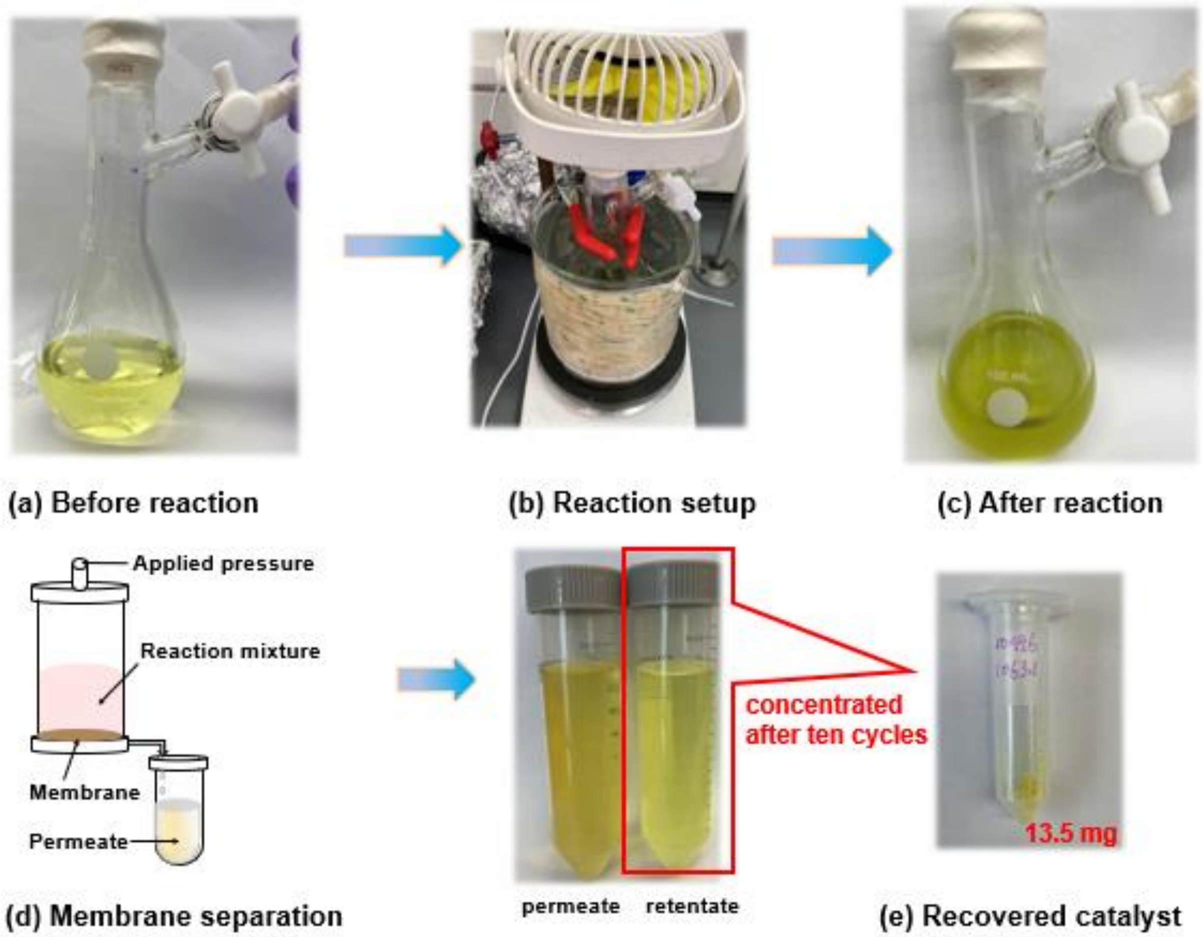

Supplementary Figure 37. Procedure for the intramolecular cycloaddition via EnT and recovery of the photocatalyst. (a-c) Photos of the photocatalysis reaction process. (d) Schematic of membrane separation and photos of permeate and retentate. (e) Photo of the recovered catalyst.

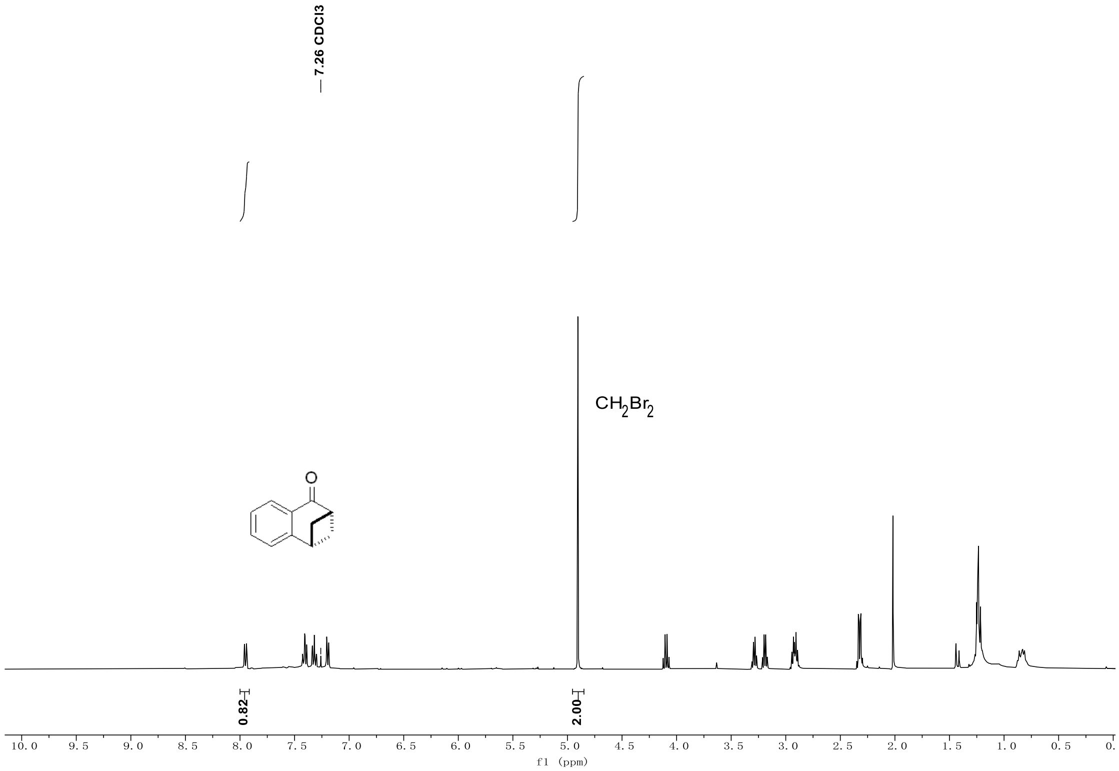

Supplementary Figure 38. Typical crude ^1H NMR spectrum of the intramolecular cycloaddition via EnT using CH_2Br_2 as an internal standard.

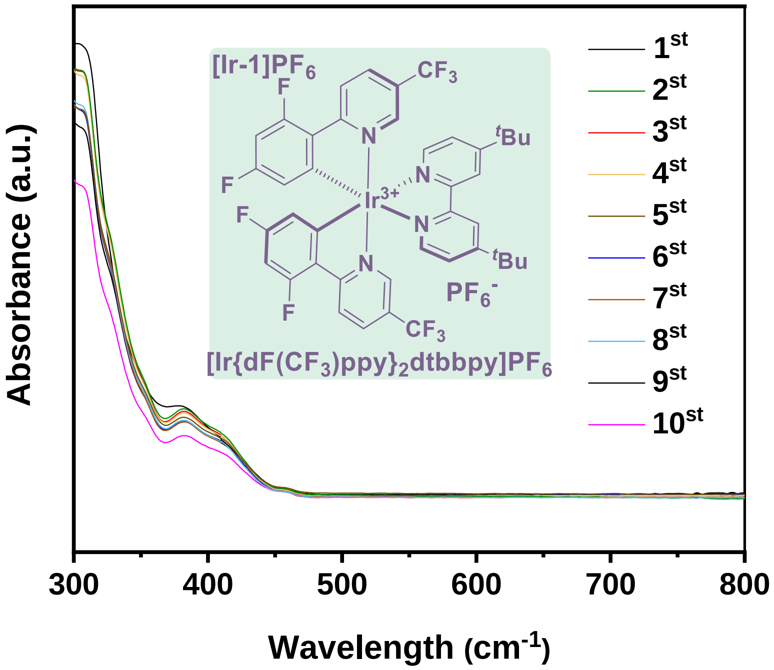

Supplementary Figure 39. UV-Vis spectra of the recovered catalyst for the intramolecular cycloaddition via EnT in 10 cycles.

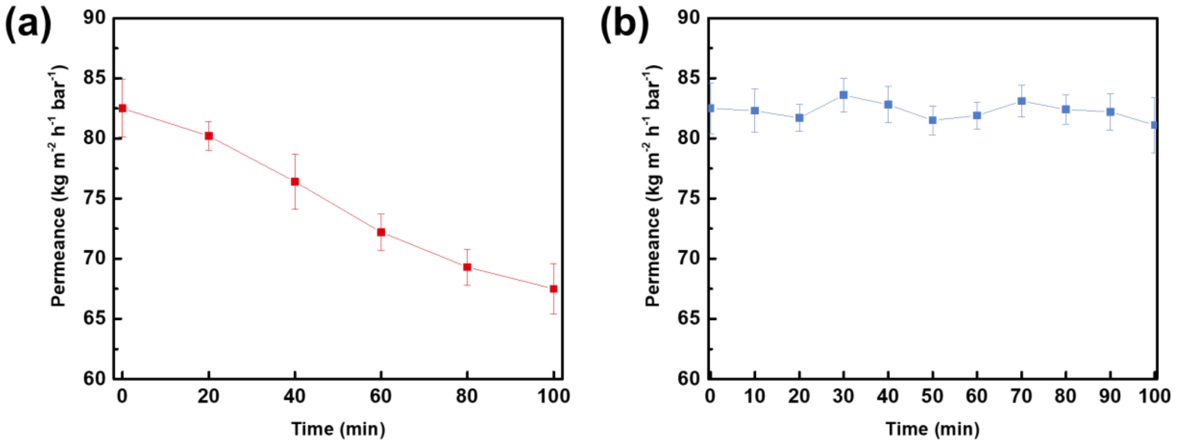

Supplementary Figure 40. (a) Permeance of catalyst recovery for the intramolecular cycloaddition via EnT as a function of time. (b) Permeance of COF membrane with a feed of a constant catalyst concentration. Error bars represent standard deviations for 3 measurements.

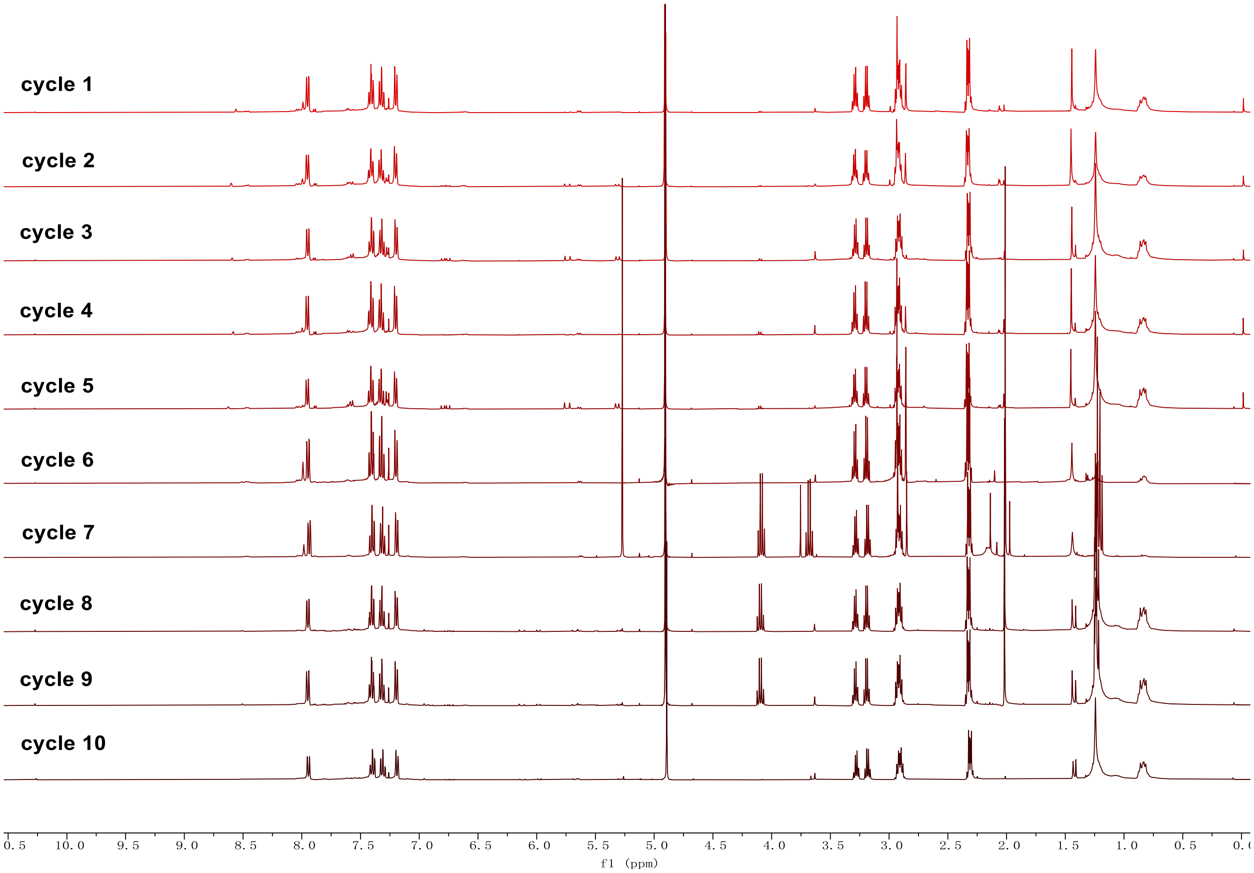

Supplementary Figure 41. Crude ^{1}H NMR spectra of ten cycles for the intramolecular cycloaddition via EnT using CH_2Br_2 as an internal standard.

Supplementary Table 4. Yields and catalyst recovery rates for the intramolecular cycloaddition via EnT in ten cycles.

Cycle	1	2	3	4	5	6	7	8	9	10
Yield (%)	80	84	84	83	82	84	86	82	80	82
[Ir-1]PF_6 (mg)	21.3	20.7	20.3	19.9	19.6	19.2	18.4	18.0	17.5	16.2
Catalyst recovery (%)	95.9	97.1	97.8	97.9	98.5	97.9	95.8	97.8	97.2	92.6

Note: As shown in **Supplementary Fig. 41** and **Supplementary Table 4**, the reactions in 10 cycles maintained steady yields and high catalytic activity, which indicates that the effective recovery and reuse of photocatalysts from the intramolecular cycloaddition reaction have been achieved by COF membranes.

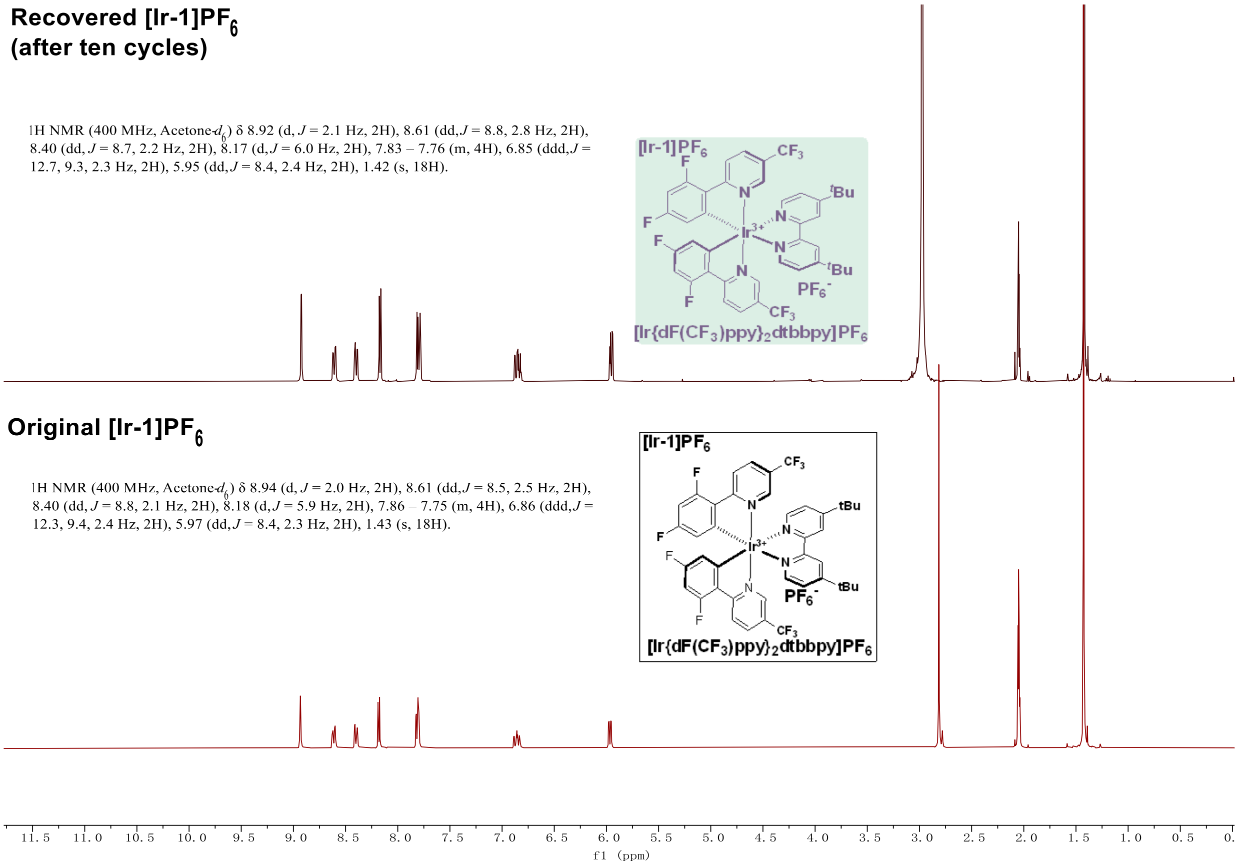

Supplementary Figure 42. ^1H NMR spectra of original and recovered photocatalyst [Ir-1]PF_6 for the intermolecular cycloaddition via EnT.

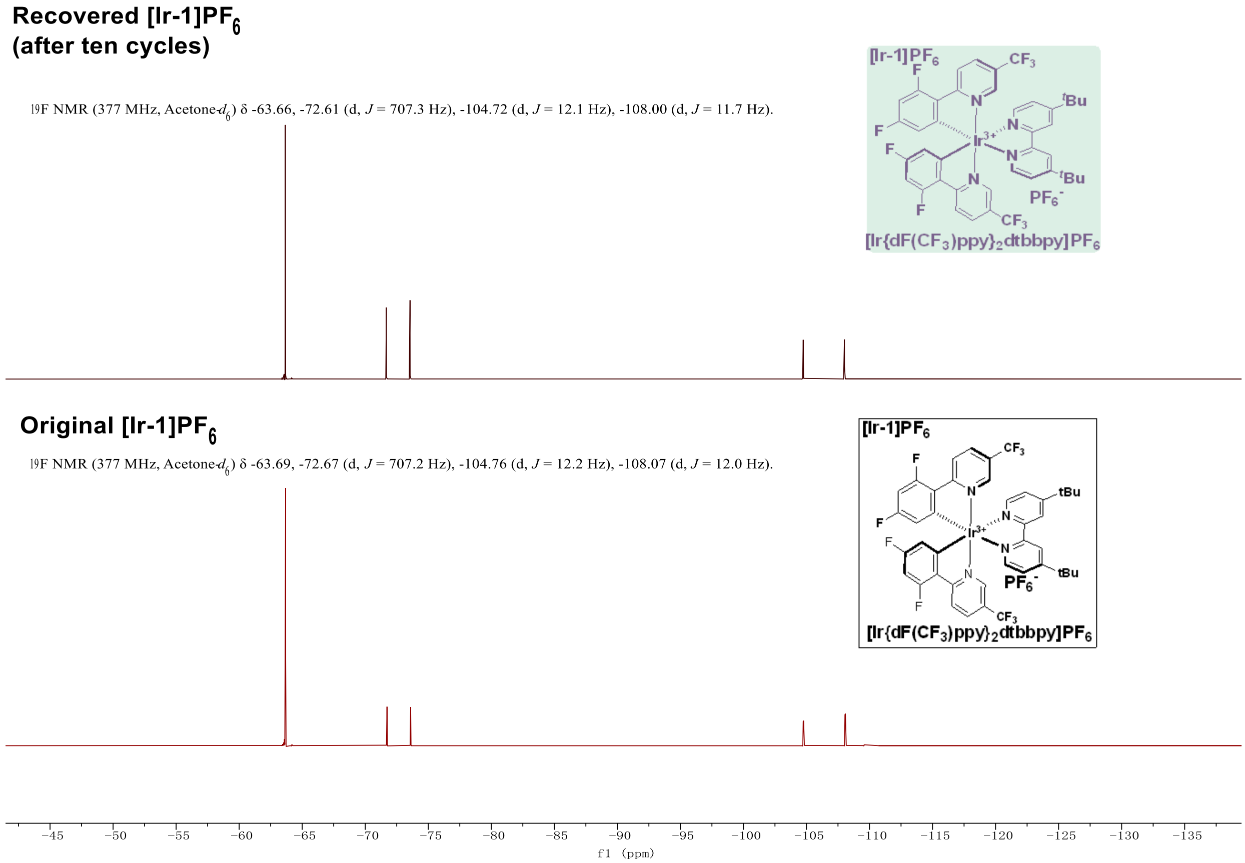

Supplementary Figure 43. ^{19}F NMR spectra of original and recovered photocatalyst [Ir-1]PF_6 for the intermolecular cycloaddition via EnT.

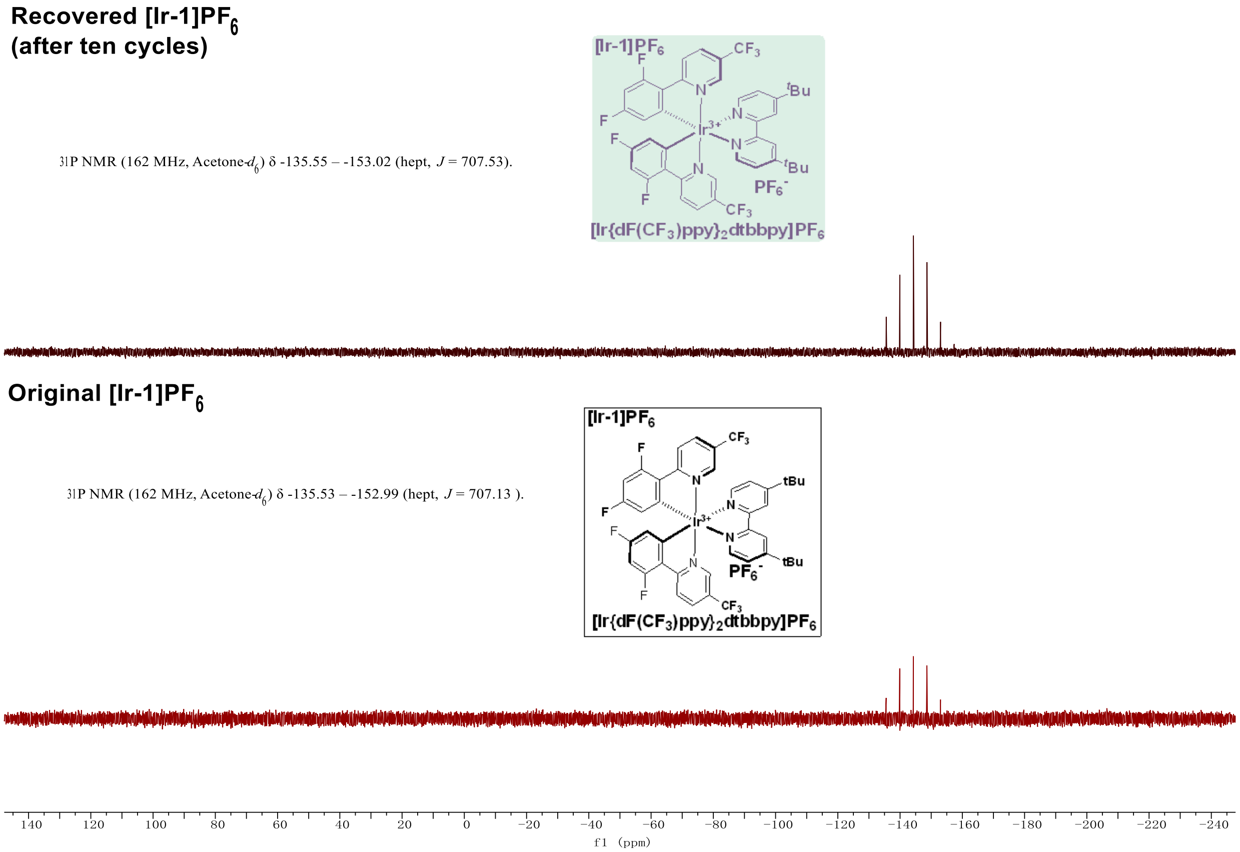

Supplementary Figure 44. ^{31}P NMR spectra of original and recovered photocatalyst [Ir-1]PF_6 for the intermolecular cycloaddition via EnT.

Note: After the final cycle, the recovered catalyst **[Ir-1]PF_6** was dried under vacuum and analyzed by NMR in acetone-*d*6. The commercial photocatalyst **[Ir-1]PF_6** was used as the original one to better identify the recovered photocatalysts. As shown in **Supplementary Figs. 42-44**, all the spectra, including ^{1}H, ^{19}F, and ^{31}P NMR of recovered catalyst **[Ir-1]PF_6**, were consistent with the original ones, indicating the high purity of the recovered noble metal catalyst with no degradation.

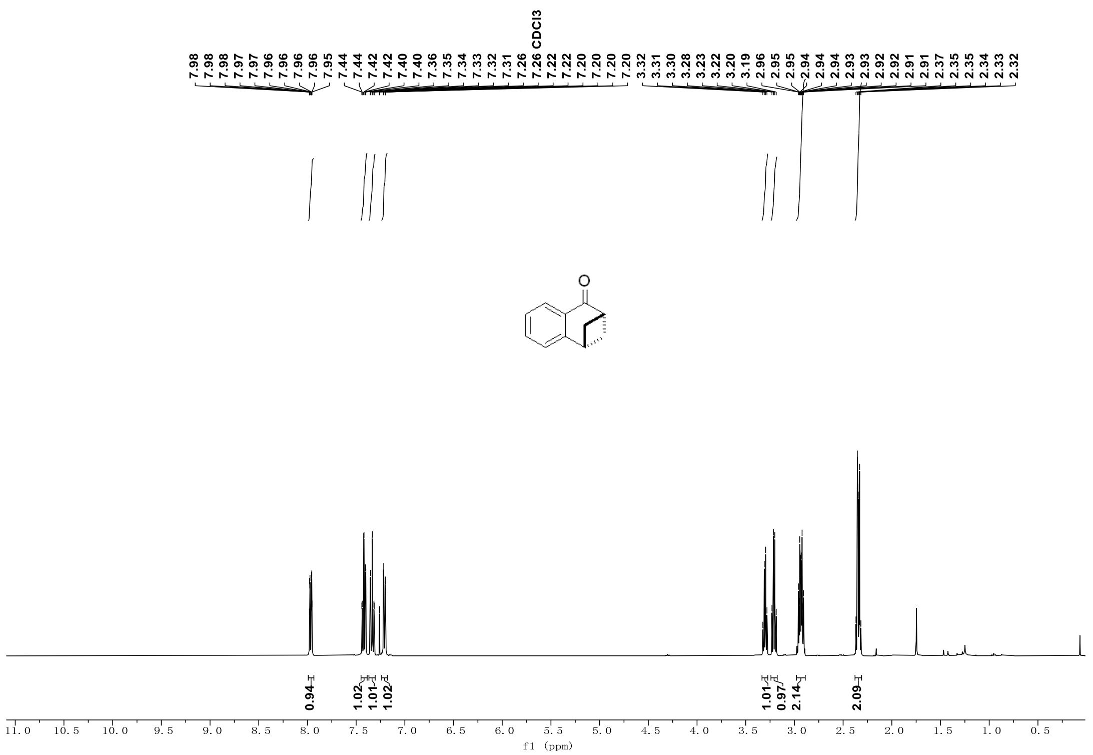

Supplementary Figure 45. ^{1}H NMR spectrum of 2,3-dihydro-1,3-methanonaphthalen-4(1H)-one.

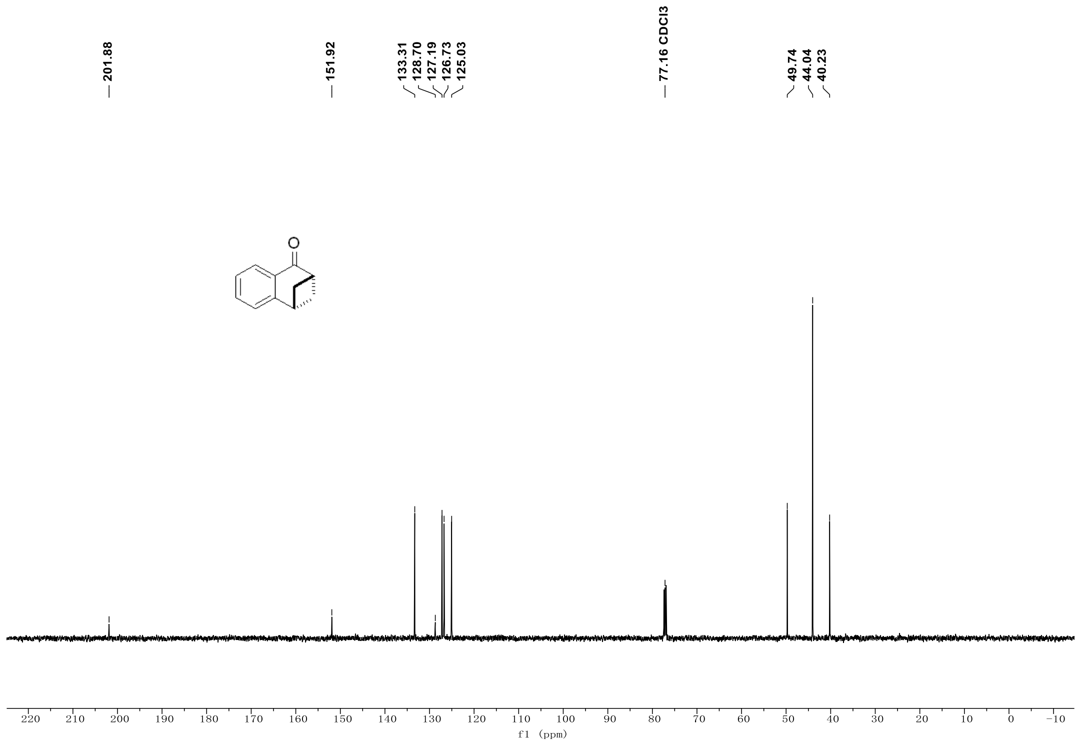

Supplementary Figure 46. ^{13}C NMR spectrum of 2,3-dihydro-1,3-methanonaphthalen-4(1H)-one.

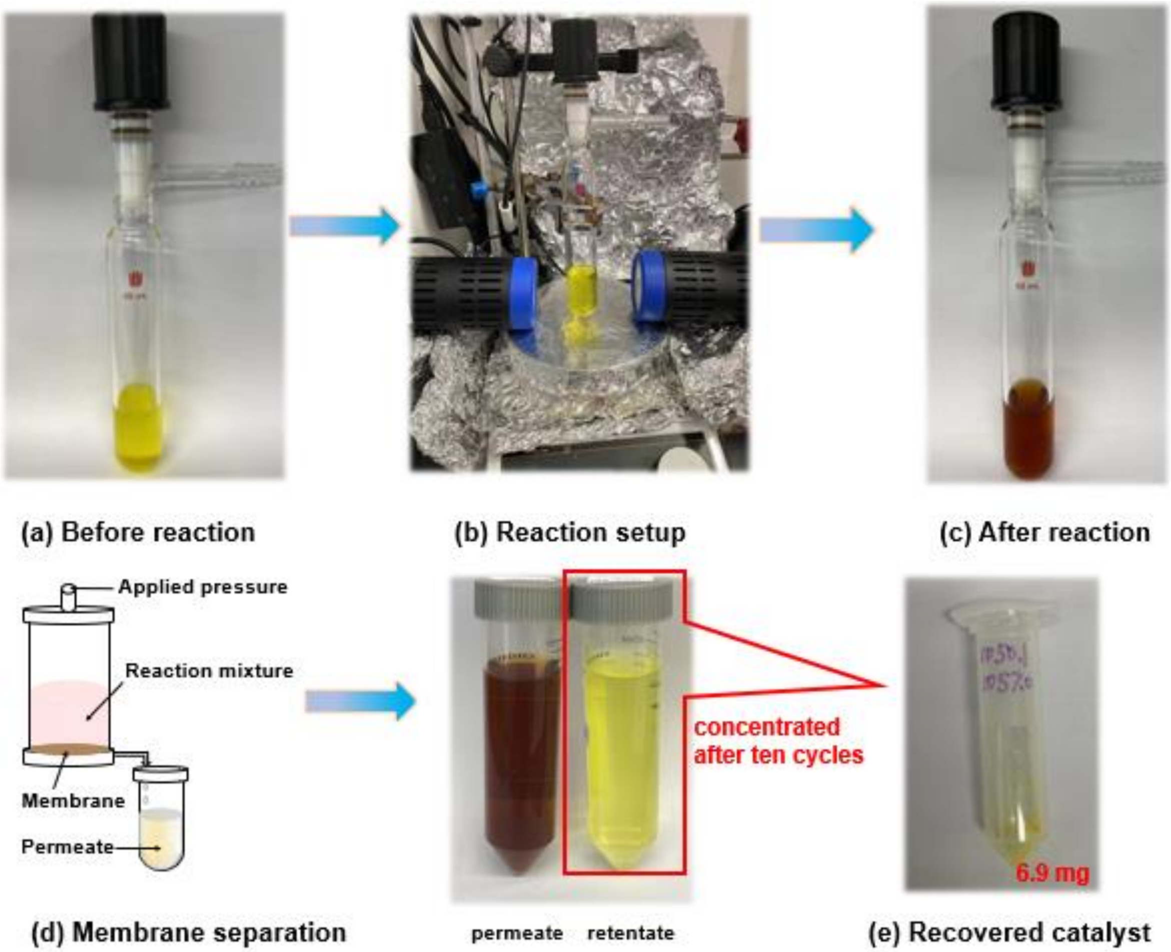

Supplementary Figure 47. Procedure for the alkylation of heteroarenes via SET and recovery of the photocatalyst. (a-c) Photos of the photocatalysis reaction process. (d) Schematic of membrane separation and photos of permeate and retentate. (e) Photo of the recovered catalyst.

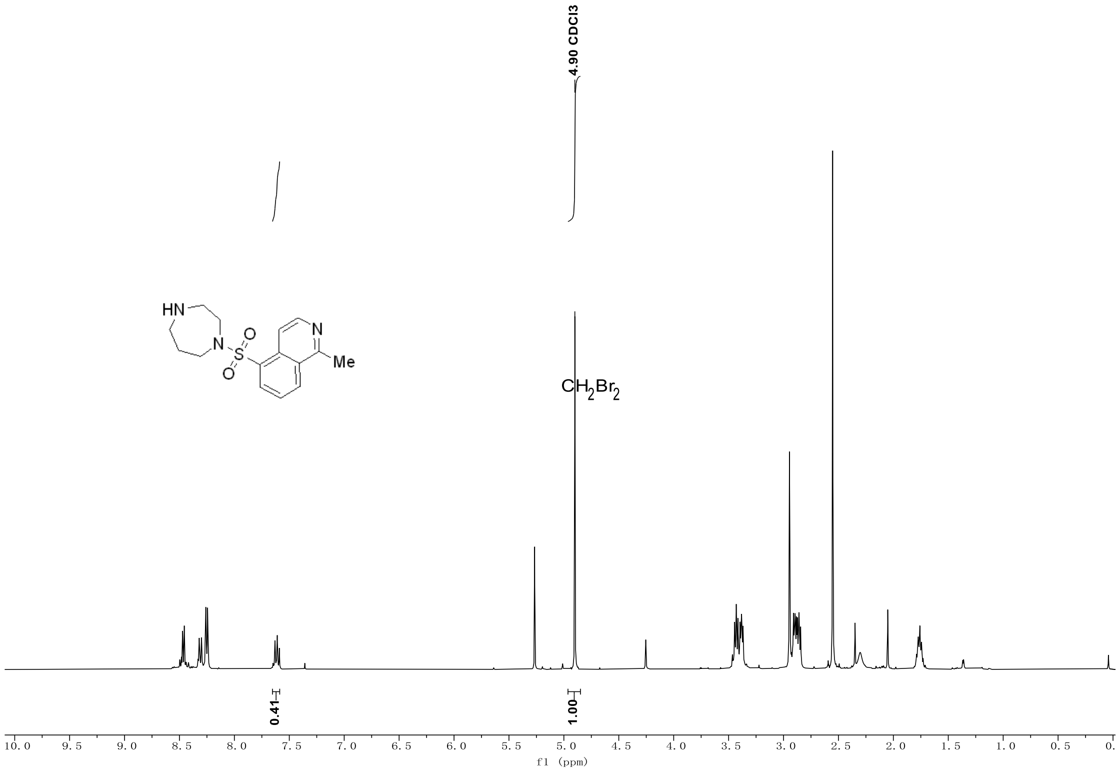

Supplementary Figure 48. Typical crude ^{1}H NMR spectrum for the alkylation of heteroarenes via SET using CH_2Br_2 as an internal standard.

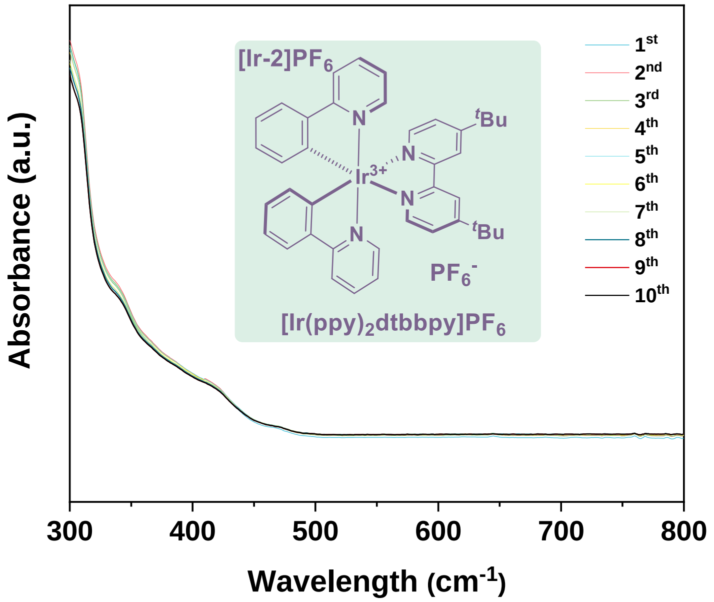

Supplementary Figure 49. UV-Vis spectra of the recovered catalyst for the alkylation of heteroarenes via SET in 10 cycles.

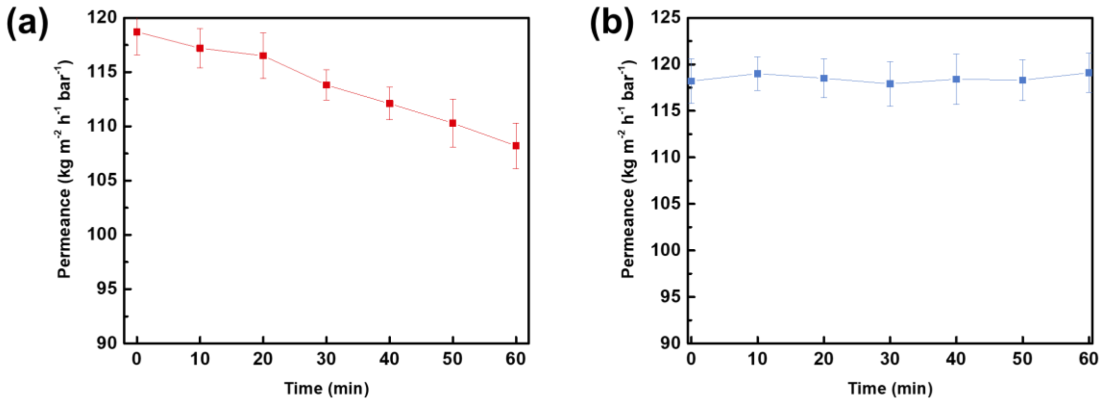

Supplementary Figure 50. (a) Permeance of catalyst recovery for the alkylation of heteroarenes via SET as a function of time. (b) Permeance of COF membrane with a feed of a constant catalyst concentration. Error bars represent standard deviations for 3 measurements.

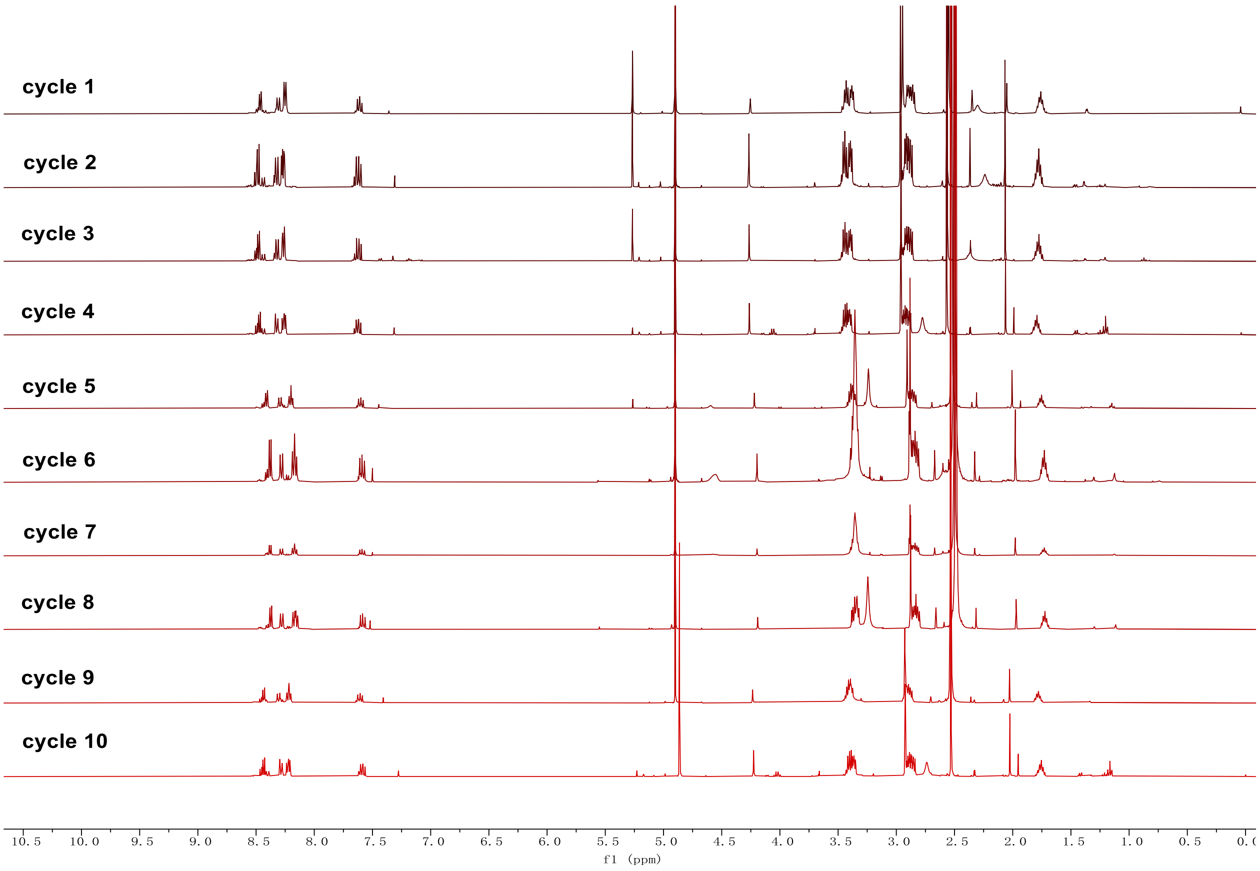

Supplementary Figure 51. Crude ^{1}H NMR spectra for the alkylation of heteroarenes via SET of 10 cycles using CH_2Br_2 as an internal standard.

Supplementary Table 5. Yields and catalyst recovery rates for the heteroarenes via SET in ten cycles.

Cycle	1	2	3	4	5	6	7	8	9	10
Yield (%)	80	82	80	81	80	83	84	82	81	80
[Ir-2]PF_6 (mg)	11.6	11.3	10.9	10.6	10.2	9.8	9.5	9.1	8.8	8.6
Catalyst recovery (%)	96.7	96.9	97.0	97.2	96.2	96.1	96.8	95.9	96.7	97.2

Note: As shown in **Supplementary Fig. 51** and **Supplementary Table 5**, the reactions in 10 cycles maintained steady yields and high catalytic activity, which indicates that the effective recovery and reuse of photocatalysts from the alkylation of heteroarenes reaction have been achieved by COF membranes.

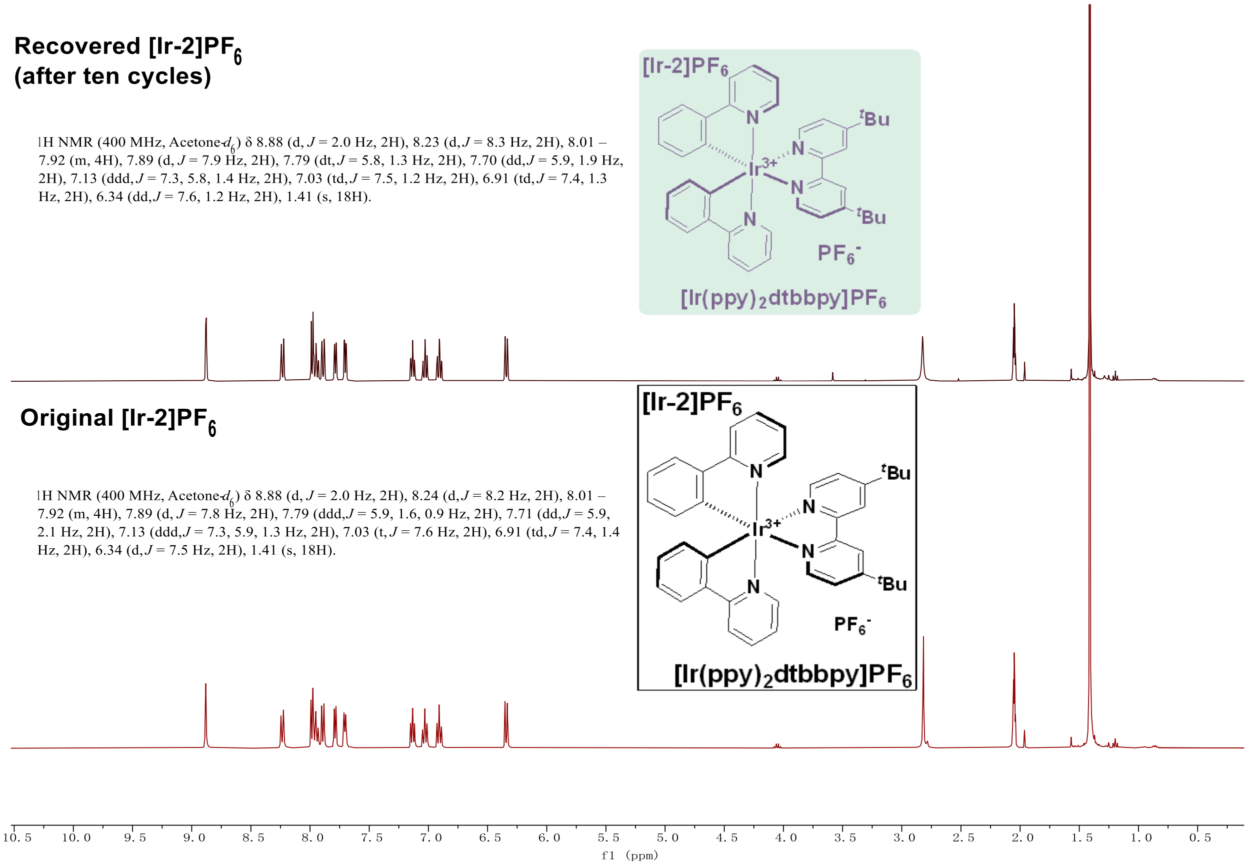

Supplementary Figure 52. ^{1}H NMR spectra of original and recovered photocatalyst [Ir-2]PF_6 for the alkylation of heteroarenes via SET.

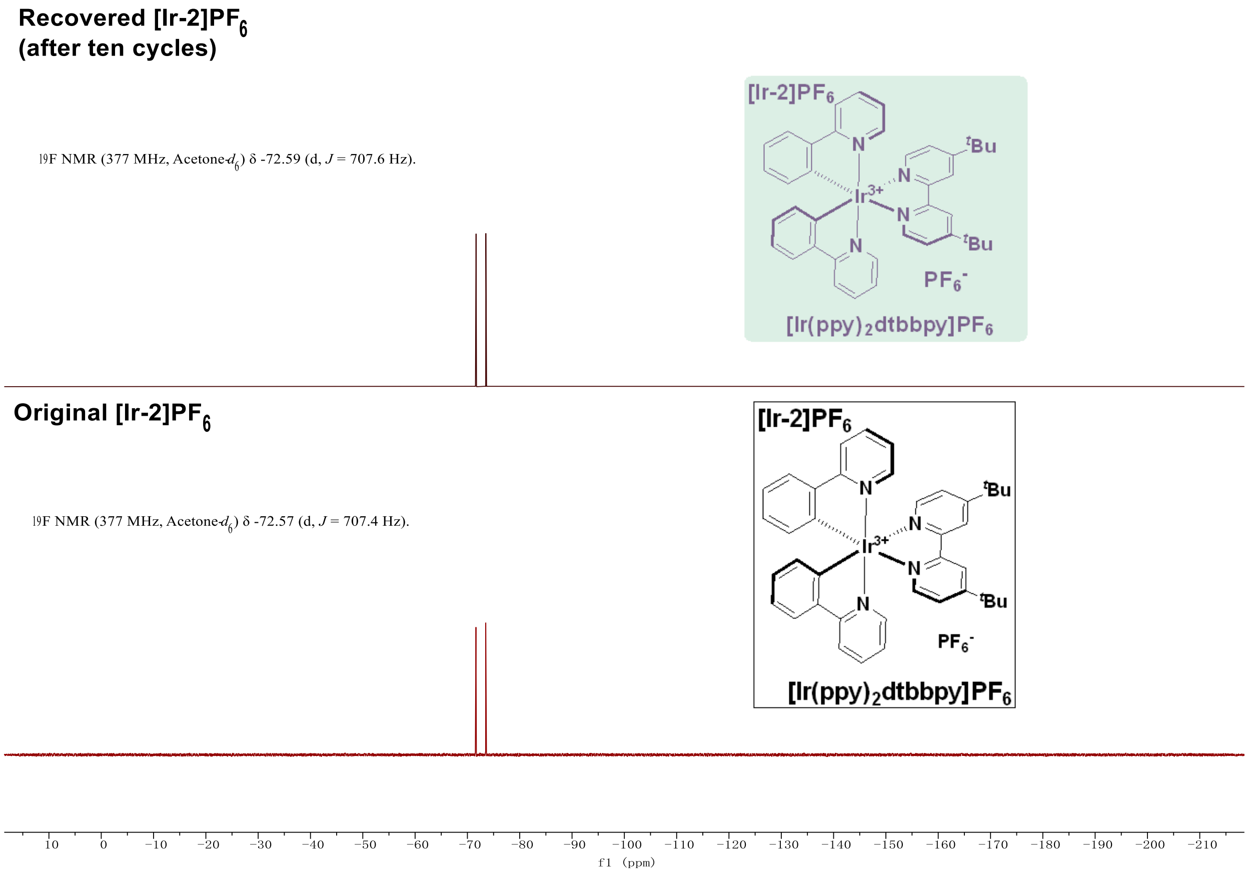

Supplementary Figure 53. ^{19}F NMR spectra of original and recovered photocatalyst [Ir-2]PF_6 for the alkylation of heteroarenes via SET.

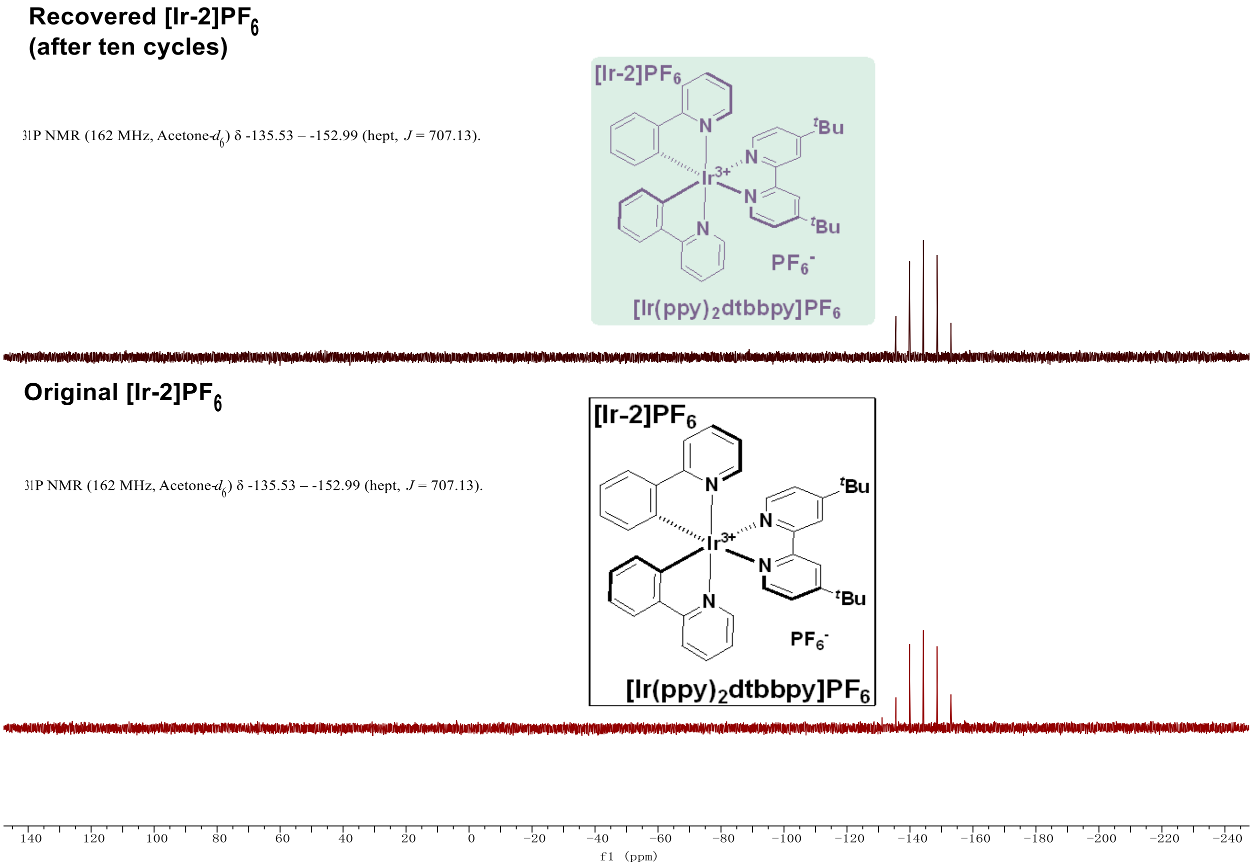

Supplementary Figure 54. ^{31}P NMR spectra of original and recovered photocatalyst [Ir-2]PF_6 for the alkylation of heteroarenes via SET.

Note: After the final cycle, the recovered catalyst **[Ir-2]PF_6** was dried under vacuum and analyzed by NMR in acetone-*d*6. The commercial photocatalyst **[Ir-2]PF_6** was used as the original one to better identify the recovered photocatalysts. As shown in **Supplementary Figs. 52-54**, all the spectra, including ^{1}H, ^{19}F, and ^{31}P NMR of recovered catalyst **[Ir-2]PF_6**, were consistent with the original ones, indicating high purity of the recovered noble metal catalyst with no degradation.

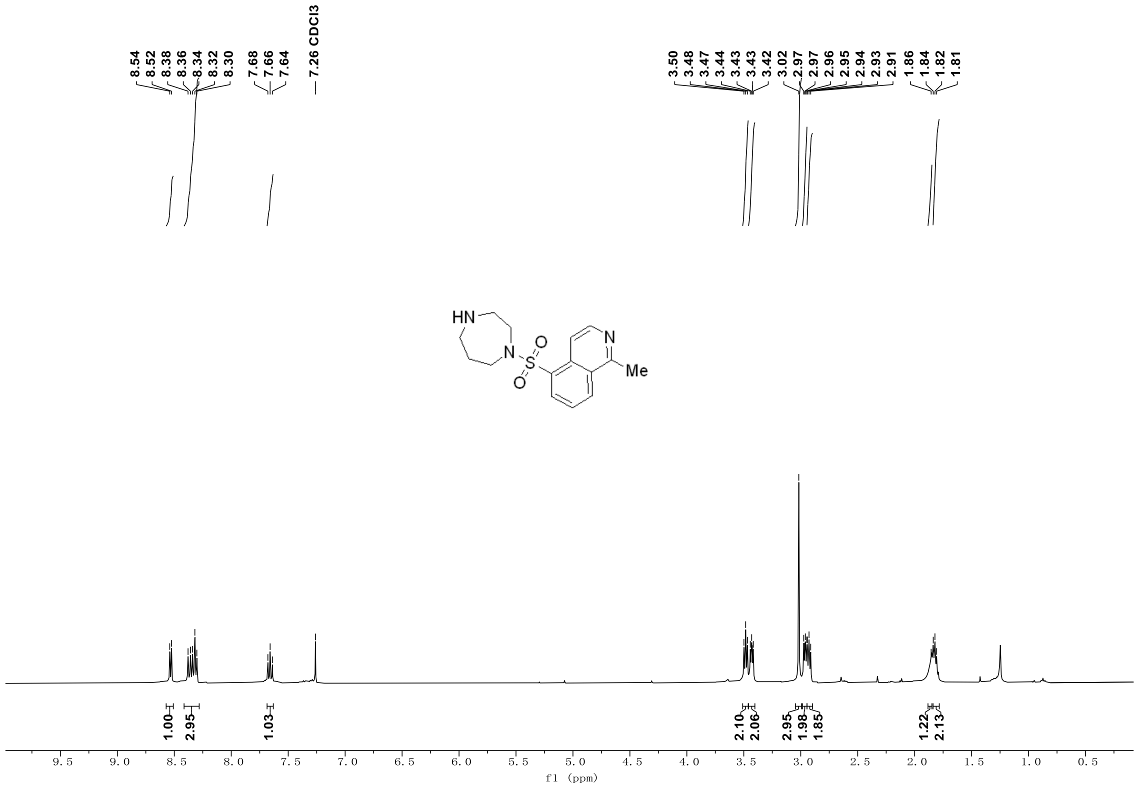

Supplementary Figure 55. ^{1}H NMR spectrum of 2,3-dihydro-1,3-methanonaphthalen-4(1H)-one.

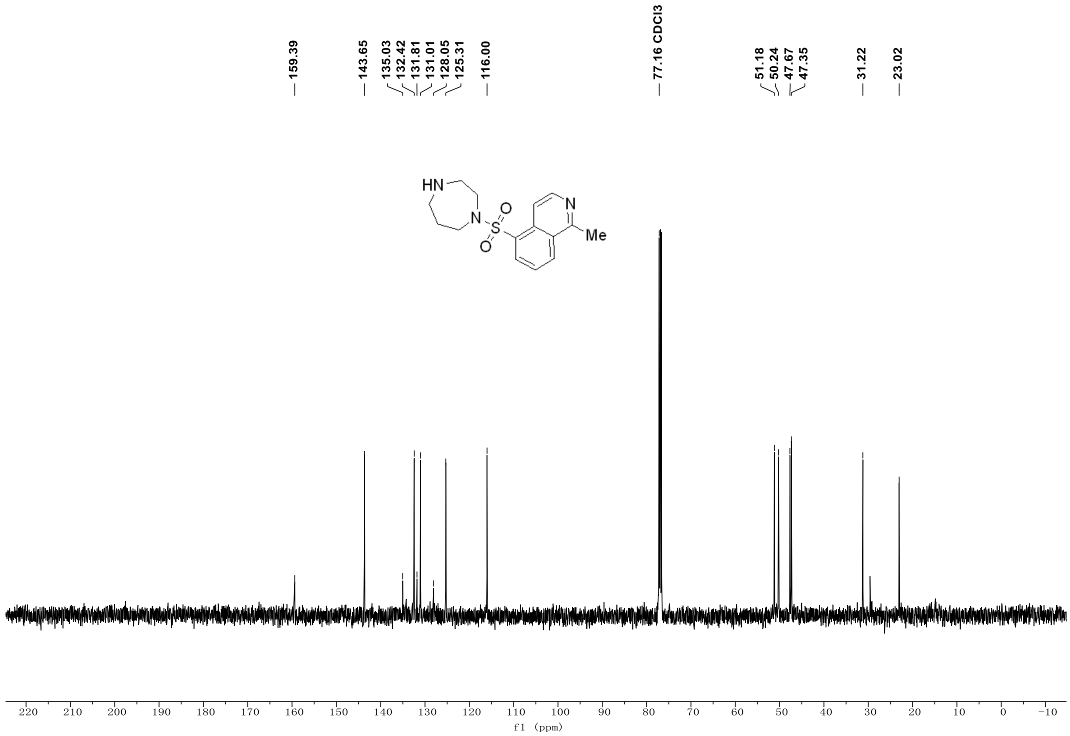

Supplementary Figure 56. ^{13}C NMR spectrum of 2,3-dihydro-1,3-methanonaphthalen-4(1H)-one.

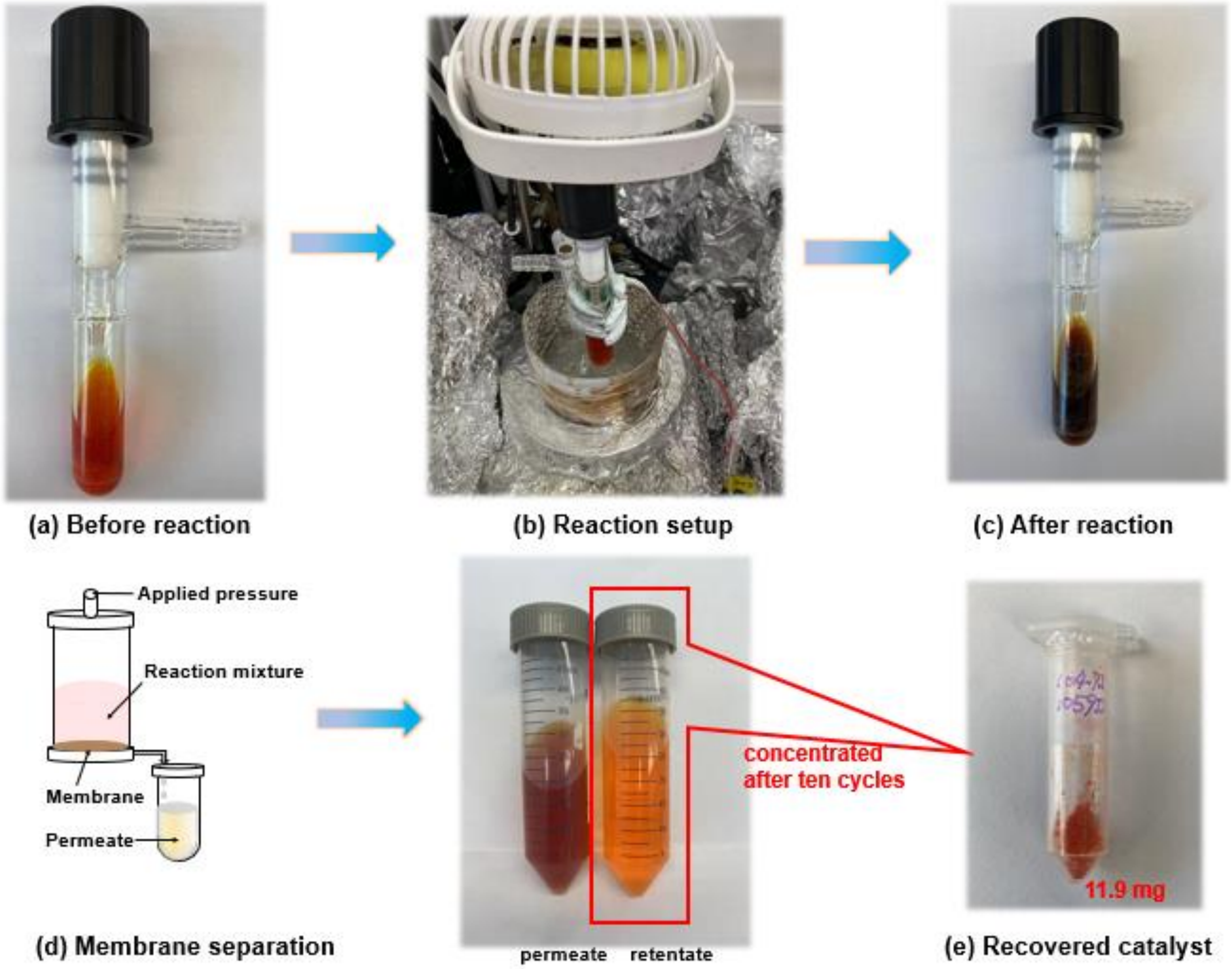

Supplementary Figure 57. Procedure for the aromatic C−H thiolation via SET and recovery of the photocatalyst. (a-c) Photos of the photocatalysis reaction process. (d) Schematic of the membrane separation and photos of permeate and retentate. (e) Photo of the recovered catalyst.

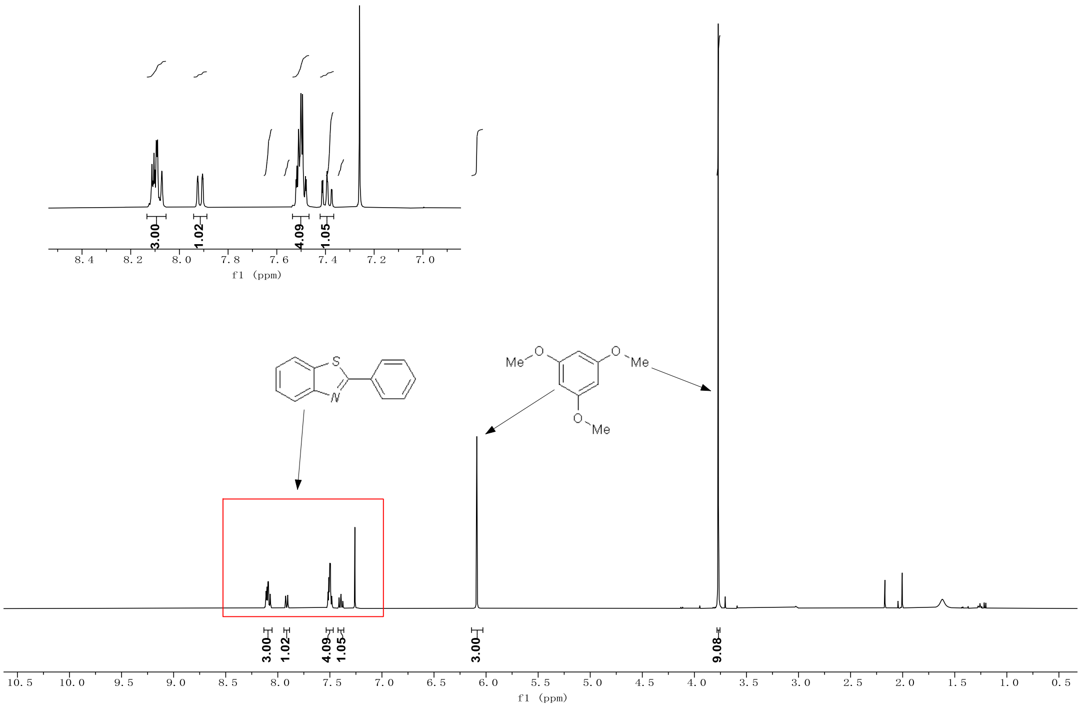

Supplementary Figure 58. Typical crude ^{1}H NMR spectrum for aromatic C−H thiolation via SET using 1,3,5-trimethoxybenzene as an internal standard.

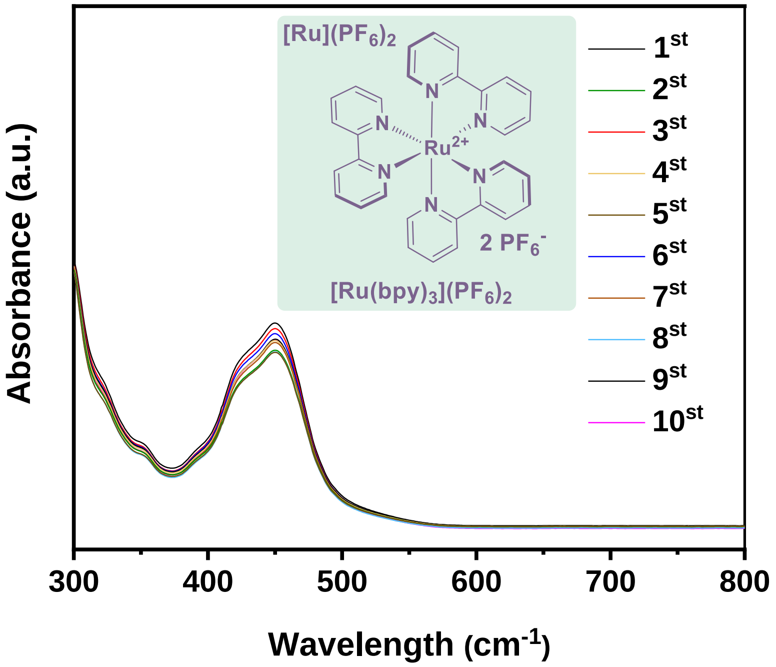

Supplementary Figure 59. UV-Vis spectra of the recovered catalyst for the aromatic C−H thiolation via SET in 10 cycles.

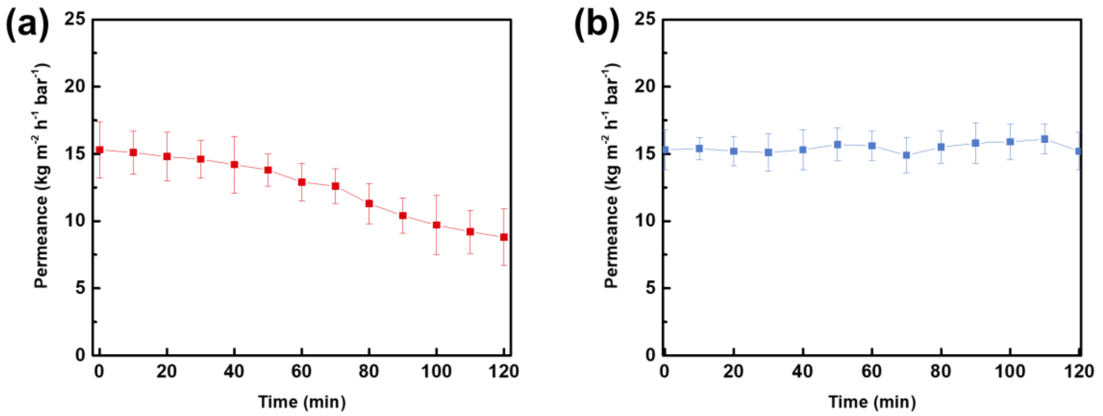

Supplementary Figure 60. (a) Permeance of catalyst recovery for the aromatic C−H thiolation via SET as a function of time. (b) Permeance of COF membrane with a feed of a constant catalyst concentration. Error bars represent standard deviations for 3 measurements.

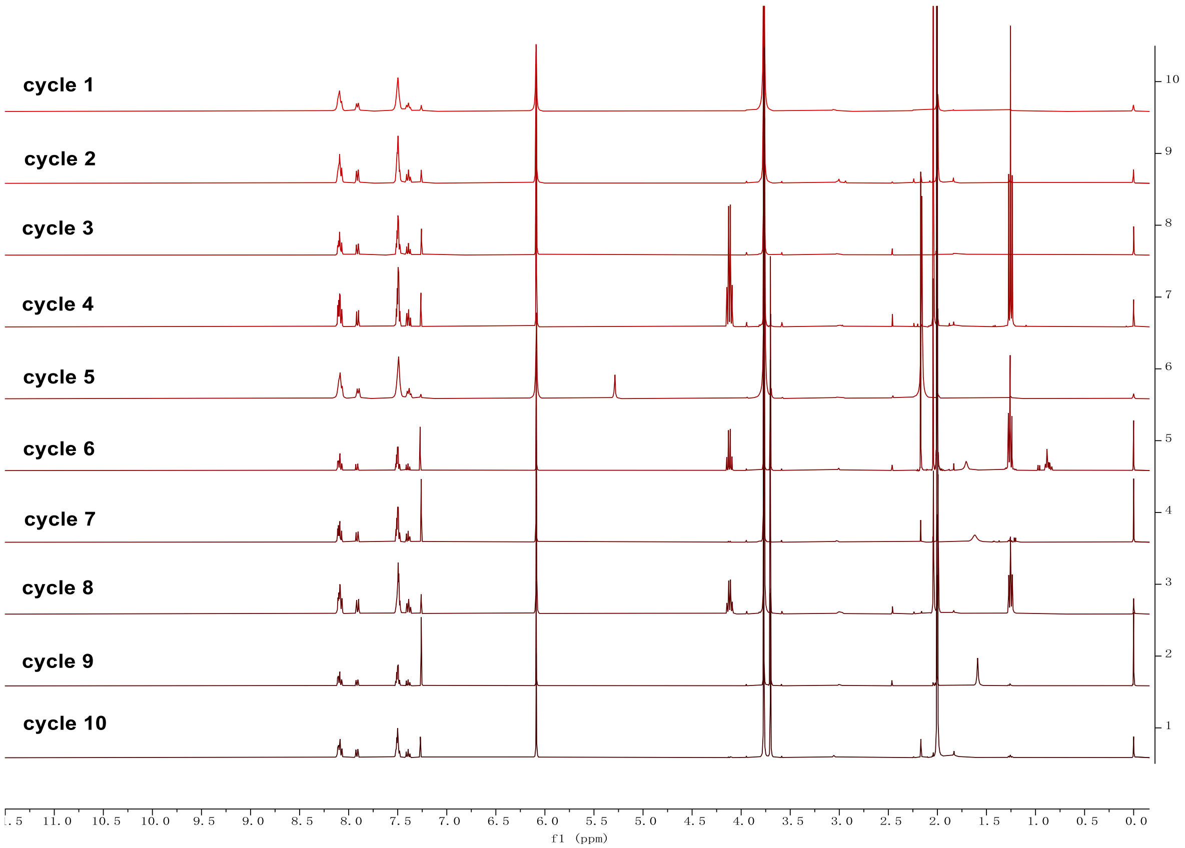

Supplementary Figure 61. Crude ^{1}H NMR spectra for the aromatic C−H thiolation via SET using 1,3,5-trimethoxybenzene as an internal standard in ten cycles.

Supplementary Table 6. Yields and catalyst recovery rates for the aromatic C−H thiolation via SET in 10 cycles.

Cycle	1	2	3	4	5	6	7	8	9	10
Yield (%)	99	98	98	98	99	98	99	99	98	98
$[Ru](PF_6)_2$ (mg)	14.3	13.8	13.5	13.2	12.8	12.6	12.2	11.8	11.5	11.2
Catalyst recovery (%)	95.3	96.5	97.1	97.3	96.8	98.4	96.8	96.7	97.5	97.4

Note: As shown in **Supplementary Fig. 61** and **Supplementary Table 6**, the reactions in 10 cycles maintained steady yields and high catalytic activity, which indicates that the effective recovery and reuse of photocatalysts from the aromatic C−H thiolation reaction have been achieved by COF membranes.

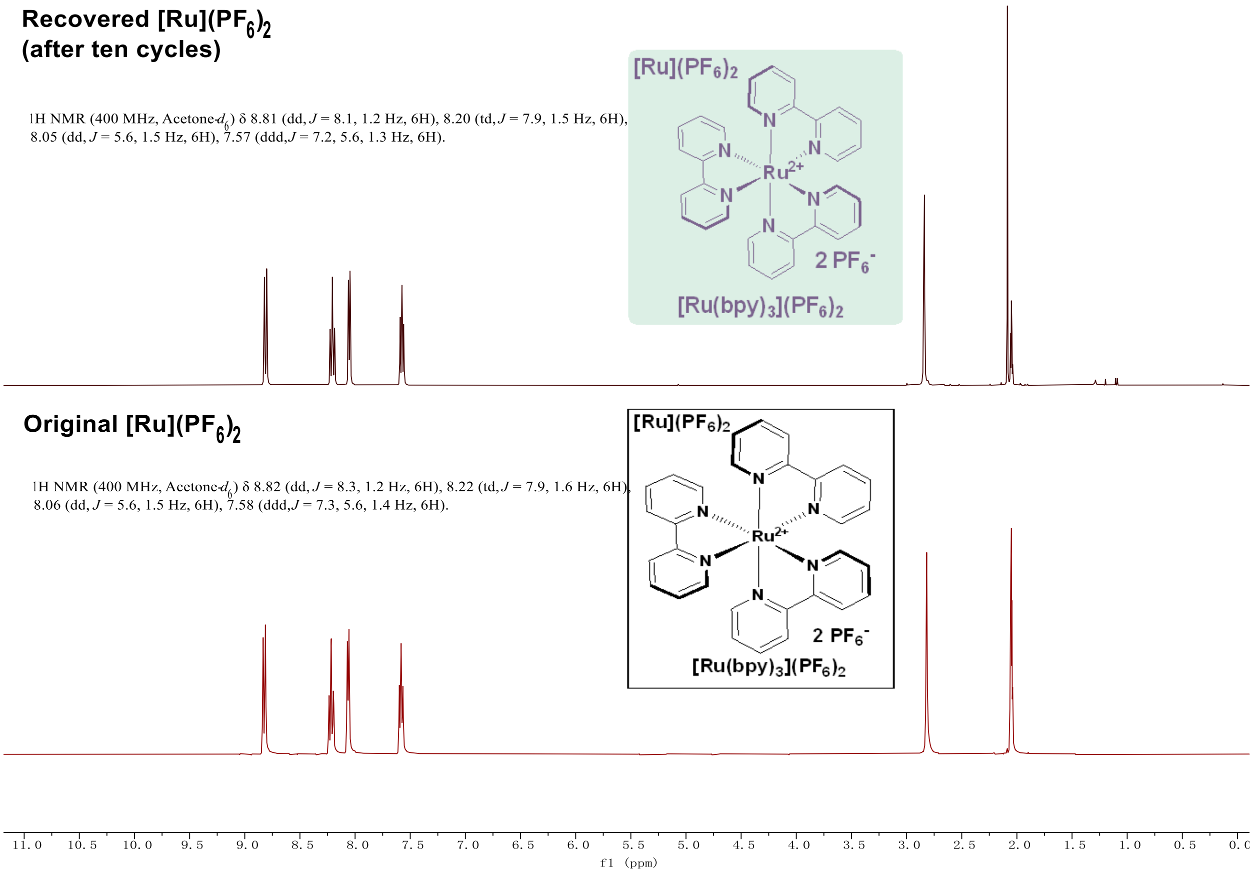

Supplementary Figure 62. ^{1}H NMR spectra of original and recovered photocatalyst $[Ru](PF_6)_2$ for the aromatic C−H thiolation via SET.

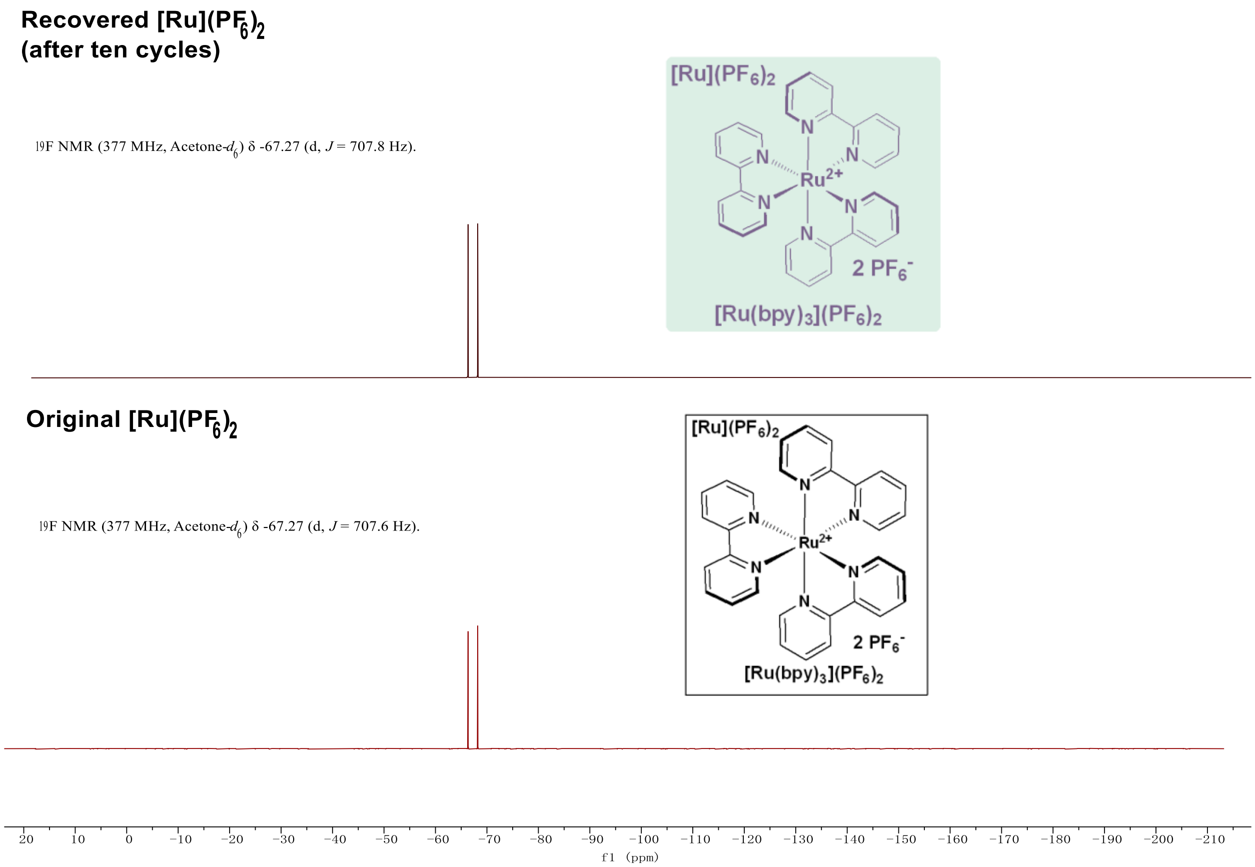

Supplementary Figure 63. ^{19}F NMR spectra of original and recovered photocatalyst $[Ru](PF_6)_2$ for the aromatic C−H thiolation via SET.

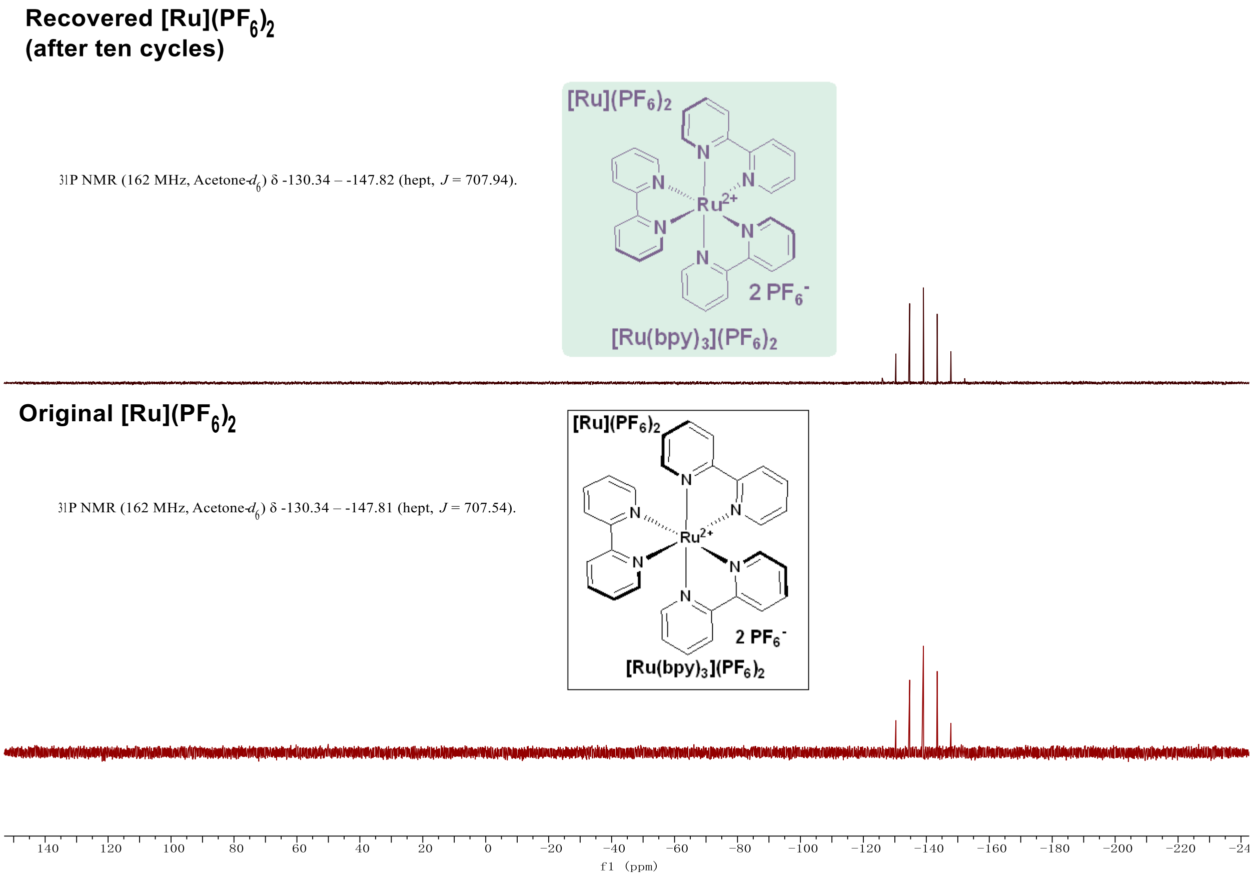

Supplementary Figure 64. ^{31}P NMR spectrum of original and recovered photocatalyst $[Ru](PF_6)_2$ for the aromatic C−H thiolation via SET.

Note: After the final cycle, the recovered catalyst $\mathbf{[Ru](PF_6)_2}$ was dried under vacuum and analyzed by NMR in acetone-d_6. The commercial photocatalyst $\mathbf{[Ru](PF_6)_2}$ was used as the original one to better identify the recovered photocatalysts. As shown in **Supplementary Figs. 62-64**, all the spectra, including ^{1}H, ^{19}F, and ^{31}P NMR of recovered catalyst $\mathbf{[Ru](PF_6)_2}$, were consistent with the original ones, indicating the high purity of the recovered noble metal catalyst with no degradation.

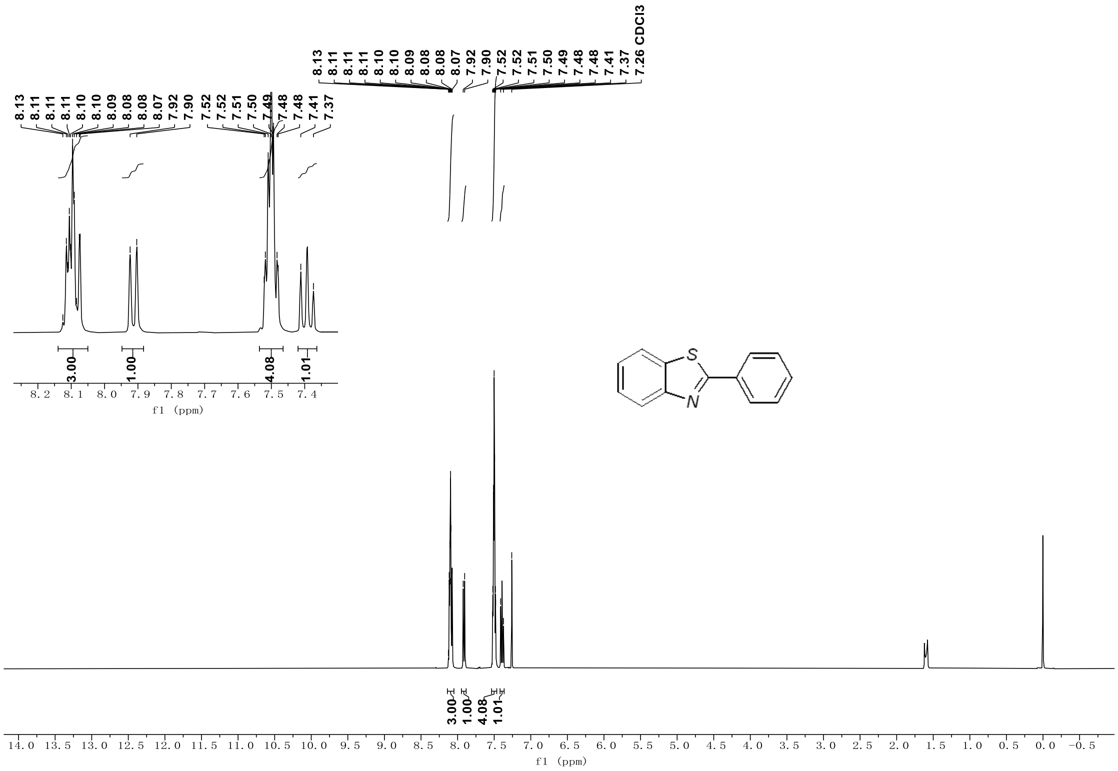

Supplementary Figure 65. ^{1}H NMR spectrum of 2-phenylbenzo[d]thiazole.

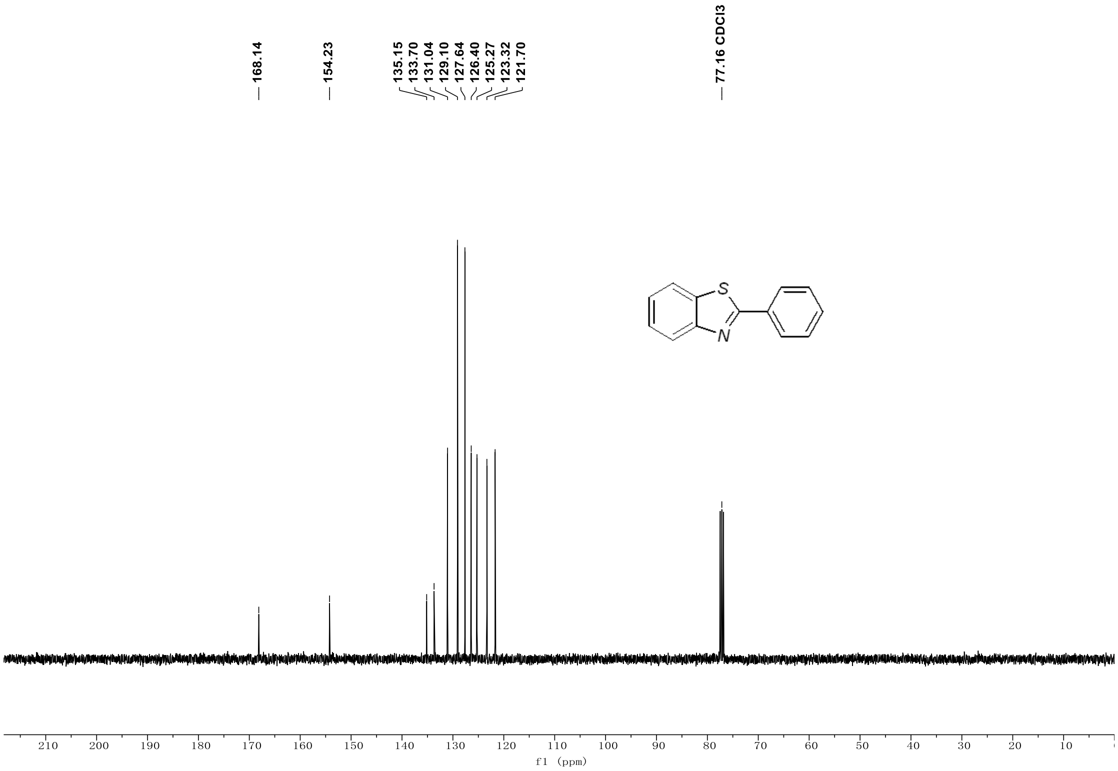

Supplementary Figure 66. ^{13}C NMR spectrum of 2-phenylbenzo[d]thiazole.

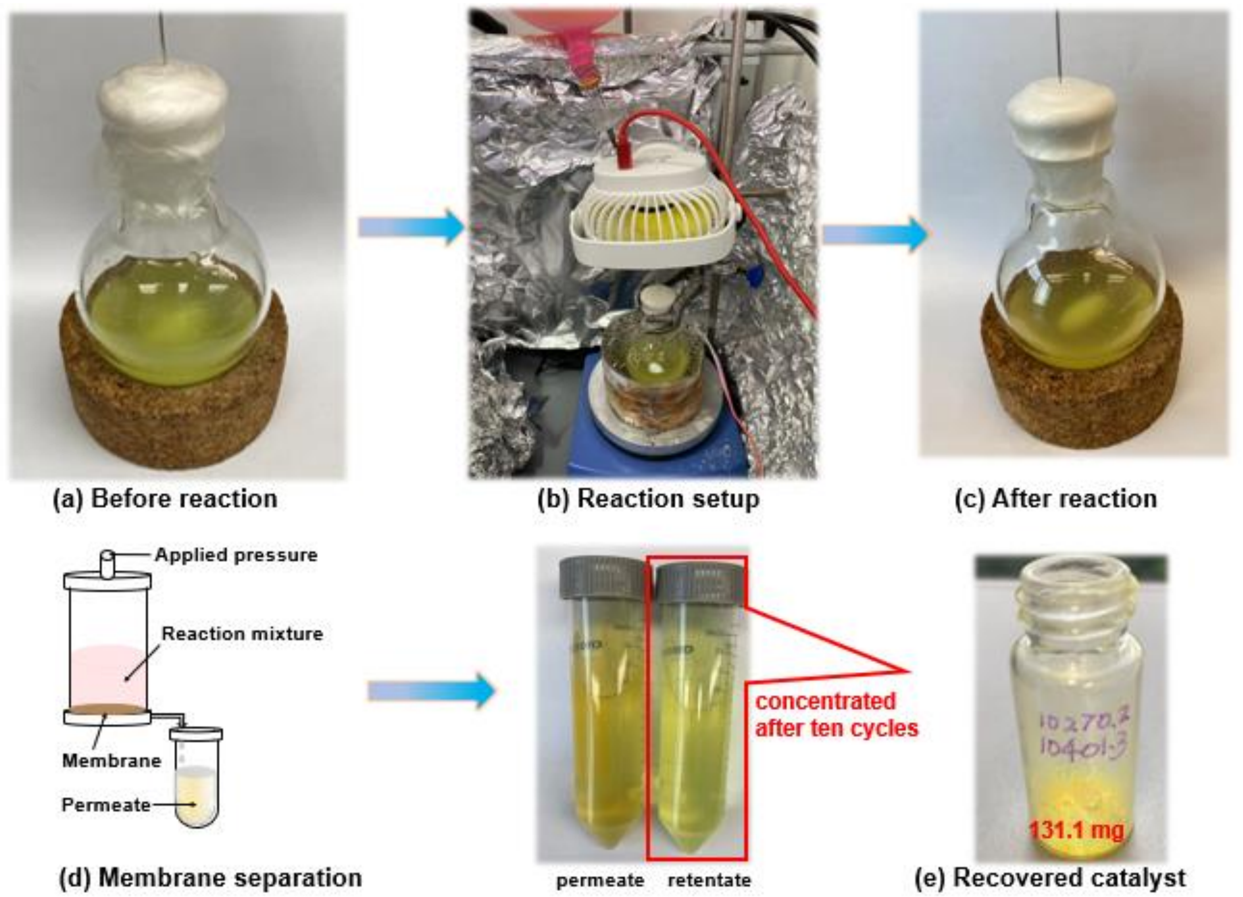

Supplementary Figure 67. Procedure for the enantioselective Minisci-type addition via SET and recovery of the photocatalysts. (a-c) Photos of the photocatalysis reaction process. (d) Schematic of membrane separation and photos of permeate and retentate. (e) Photo of the recovered catalyst.

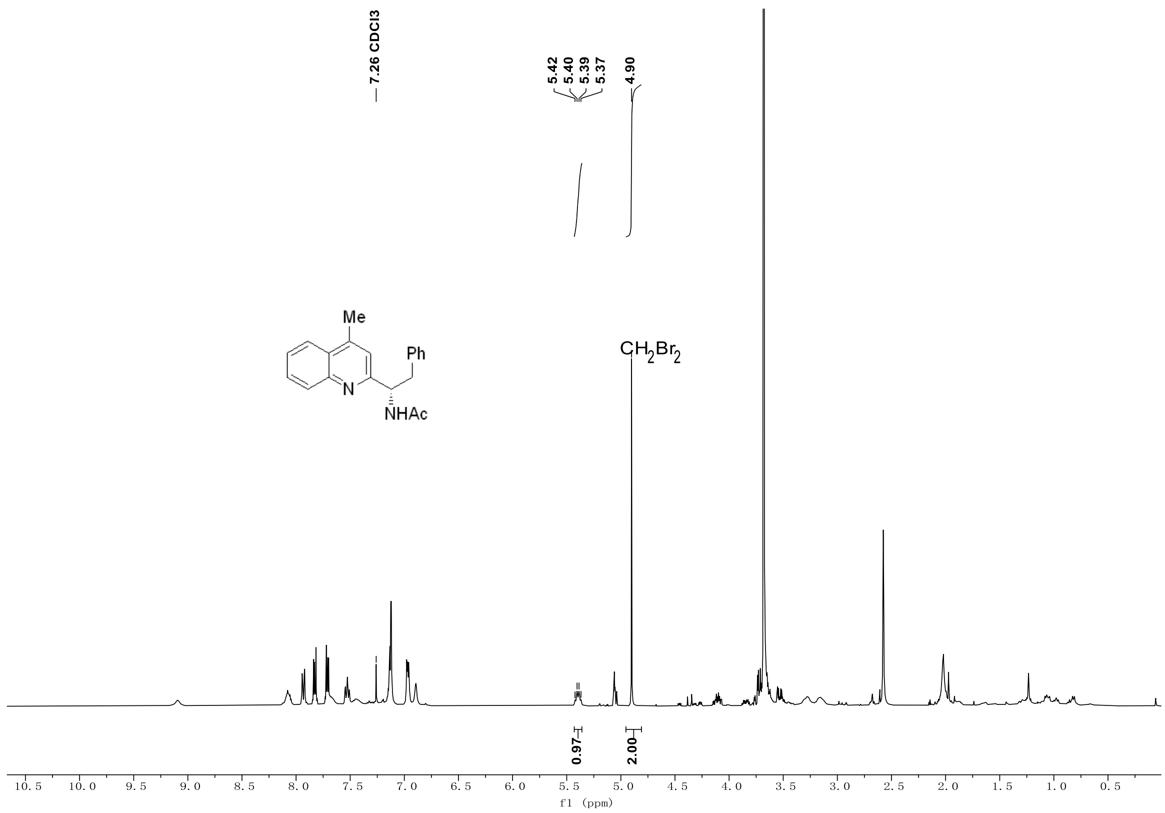

Supplementary Figure 68. Typical crude ^{1}H NMR spectrum for the enantioselective Minisci-type addition via SET using CH_2Br_2 as an internal standard.

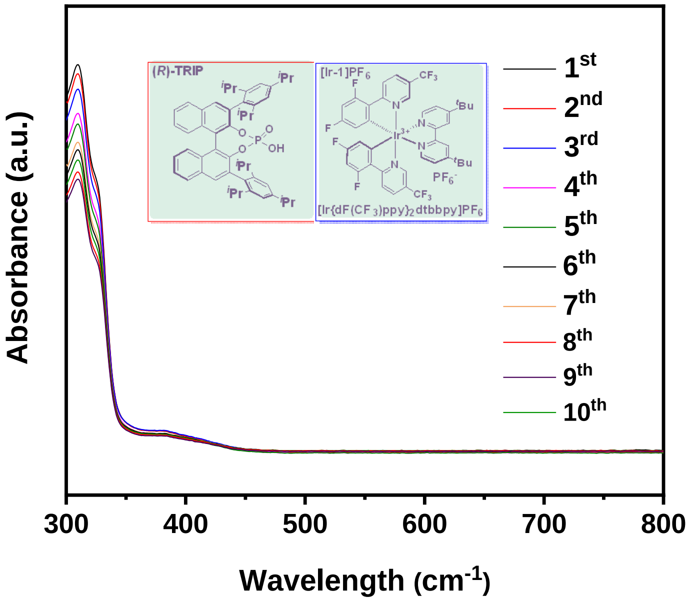

Supplementary Figure 69. UV-Vis spectra of the recovered catalyst in 10 cycles for the enantioselective Minisci-type addition via SET.

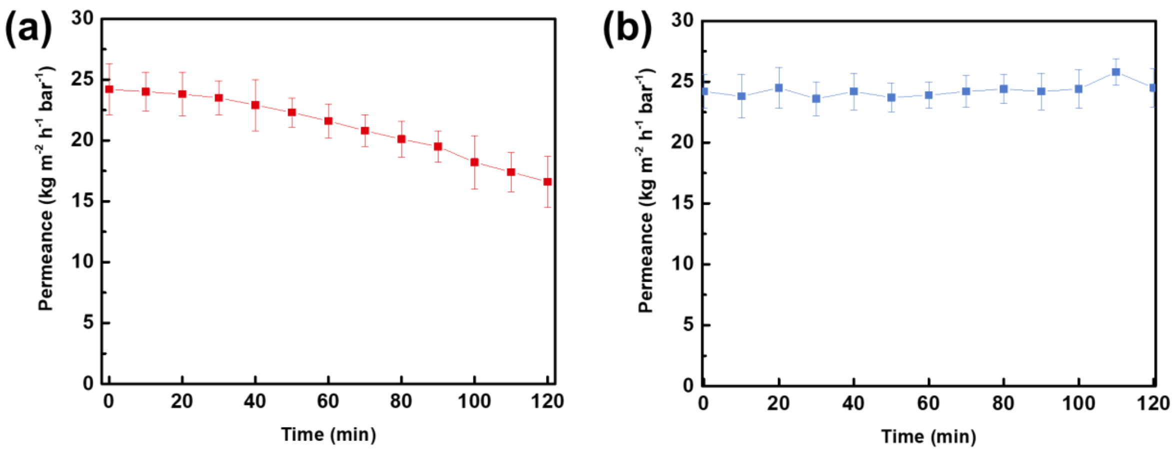

Supplementary Figure 70. (a) Permeance of catalyst recovery for the enantioselective Minisci-type addition via SET as a function of time. (b) Permeance of COF membrane with a feed of a constant catalyst concentration. Error bars represent standard deviations for 3 measurements.

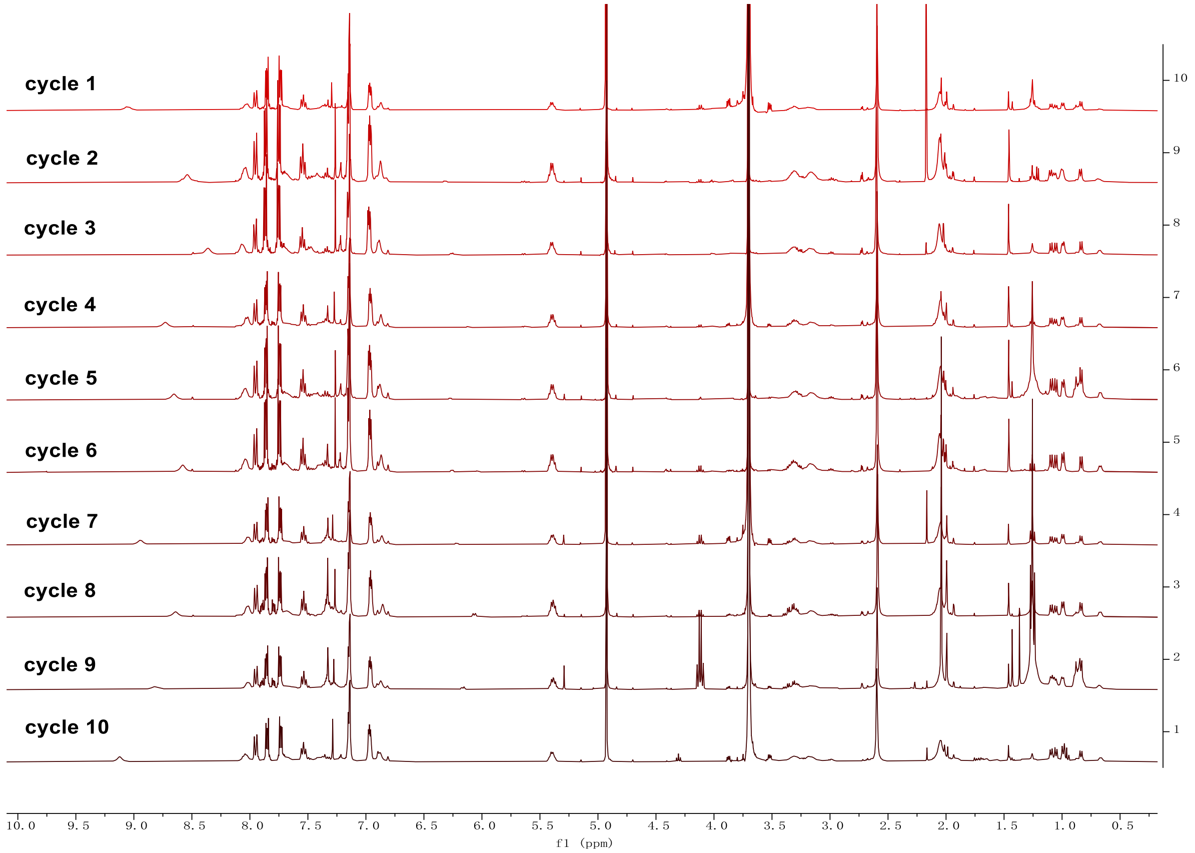

Supplementary Figure 71. Crude ^{1}H NMR spectra of ten cycles for the enantioselective Minisci-type addition via SET using CH_2Br_2 as an internal standard.

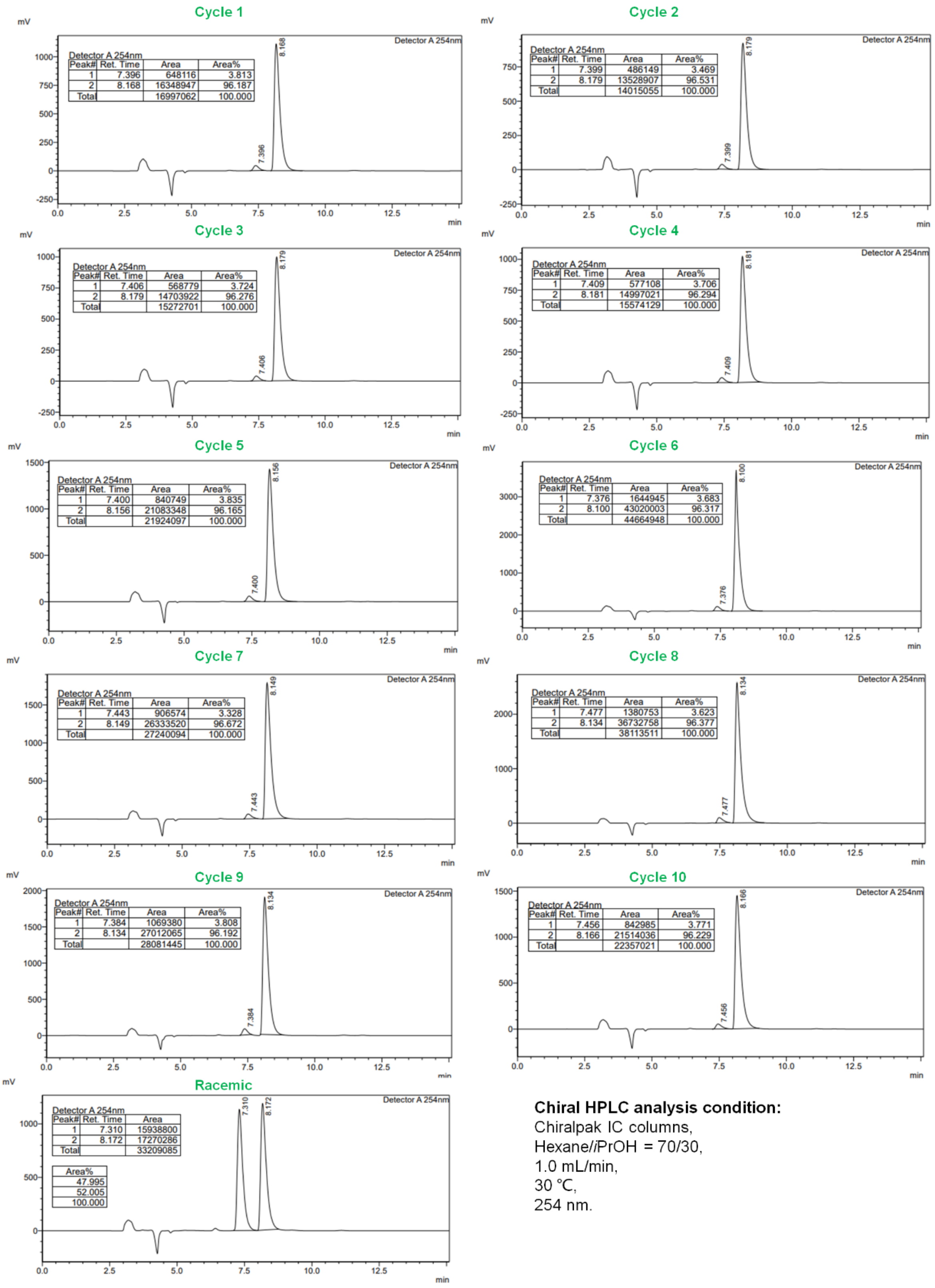

Chiral HPLC analysis condition:
Chiralpak IC columns,
Hexane/*i*PrOH = 70/30,
1.0 mL/min,
30 ℃,
254 nm.

Supplementary Figure 72. Chiral HPLC results of product for the enantioselective Minisci-type addition in each cycle.

Supplementary Table 7. Yields and catalyst recovery rates for the enantioselective Minisci-type addition via SET in ten cycles.

Cycle	1	2	3	4	5	6	7	8	9	10
Yield (%)	96	98	98	96	96	96	96	95	94	94
ee (%)	92.4	93	92.6	92.6	92.4	92.7	93.4	92.8	92.4	92.4
[Ir-1]PF_6 (mg)	40	39	38	37	36	35	34	33	32	30
[Ir-1]PF_6 recovery (%)	95.2	96.1	96.8	96.6	97.2	97.1	97.1	96.1	96.7	95.9
(R)-TRIP (mg)	137	135	133	131	128	125	122	117	114	110
(R)-TRIP recovery (%)	97.1	98.5	98.5	98.4	97.7	97.6	96.8	95.9	97.4	96.1

Note: As shown in **Supplementary Figs. 71-72** and **Supplementary Table 7**, the reactions in 10 cycles maintained steady yields and high catalytic activity, which indicates that the effective recovery and reuse of photocatalysts from the enantioselective Minisci-type addition reaction have been achieved by COF membranes nanofiltrations.

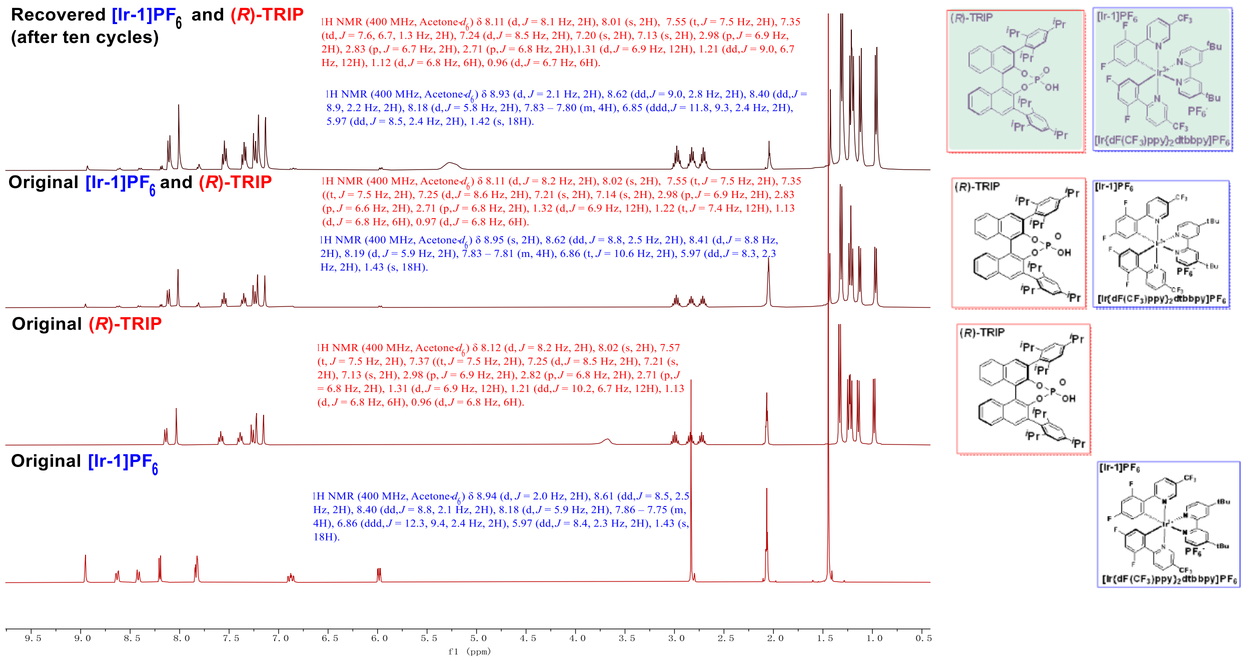

Supplementary Figure 73. ^1H NMR spectra of original and recovered photocatalyst [Ir-1]PF_6 and (*R*)-TRIP for the enantioselective Minisci-type addition via SET.

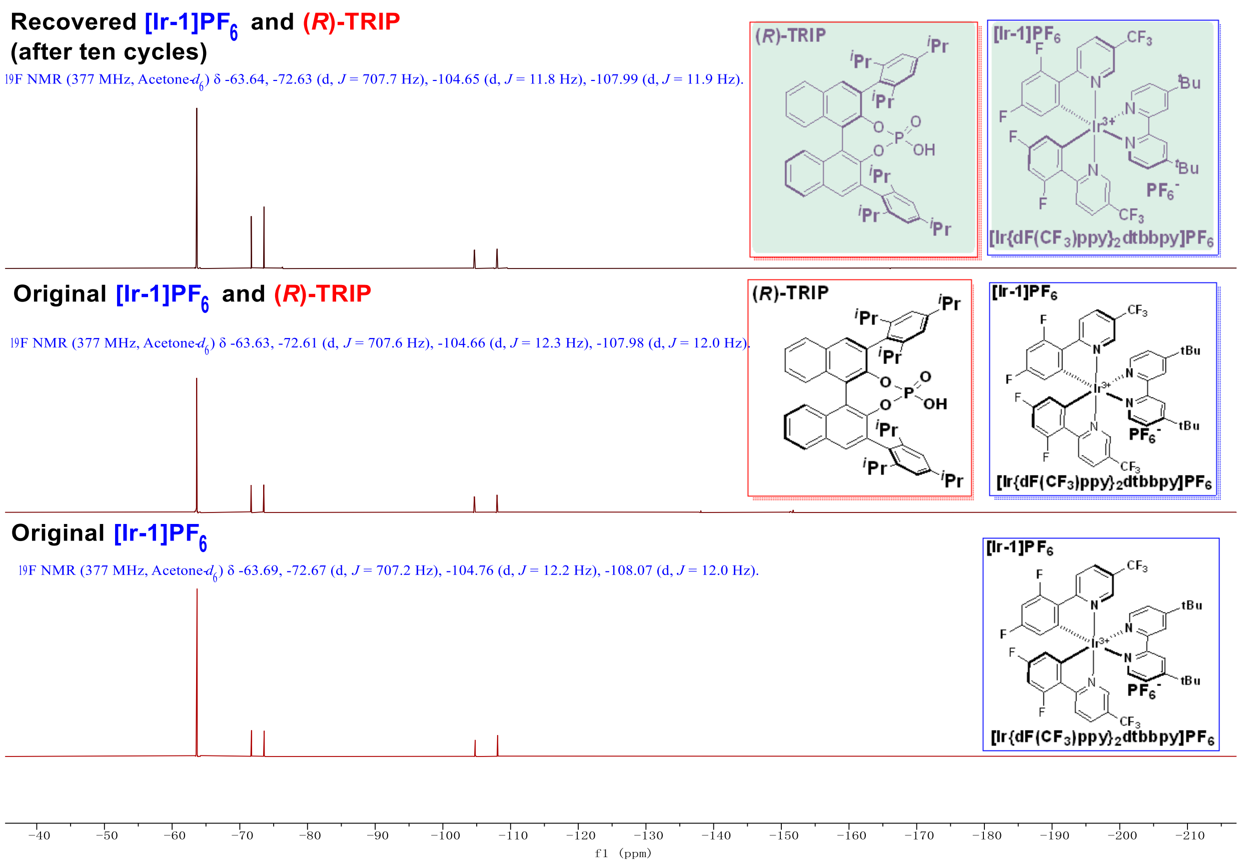

Supplementary Figure 74. ^{19}F NMR spectra of original and recovered photocatalyst [Ir-1]PF_6 and (*R*)-TRIP for the enantioselective Minisci-type addition via SET.

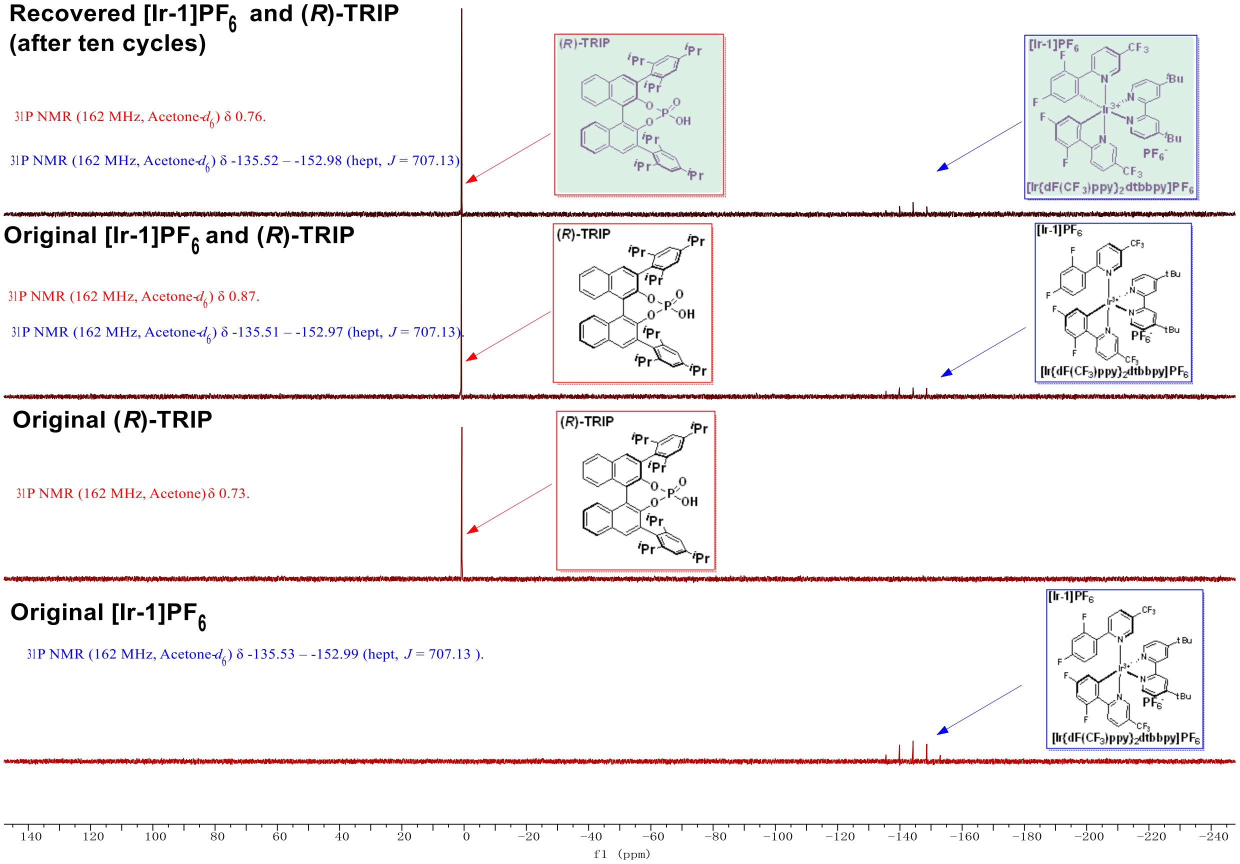

Supplementary Figure 75. ^{31}P NMR spectra of the original and recovered photocatalyst [Ir-1]PF_6 and (*R*)-TRIP for the enantioselective Minisci-type addition via SET.

Note: After the final cycle, the recovered mixture of catalysts **[Ir-1]PF_6** and **(*R*)-TRIP** was dried under vacuum and analyzed by NMR in acetone-d_6. The commercial catalysts **[Ir-1]PF_6** and **(*R*)-TRIP** were used as the original ones to better identify the recovered catalysts. As shown in **Supplementary Figs. 73-75**, all the spectra, including ^{1}H, ^{19}F, and ^{31}P NMR of recovered catalysts **[Ir-1]PF_6** and **(*R*)-TRIP**, were consistent with the original ones, indicating no degradation of both recovered catalysts.

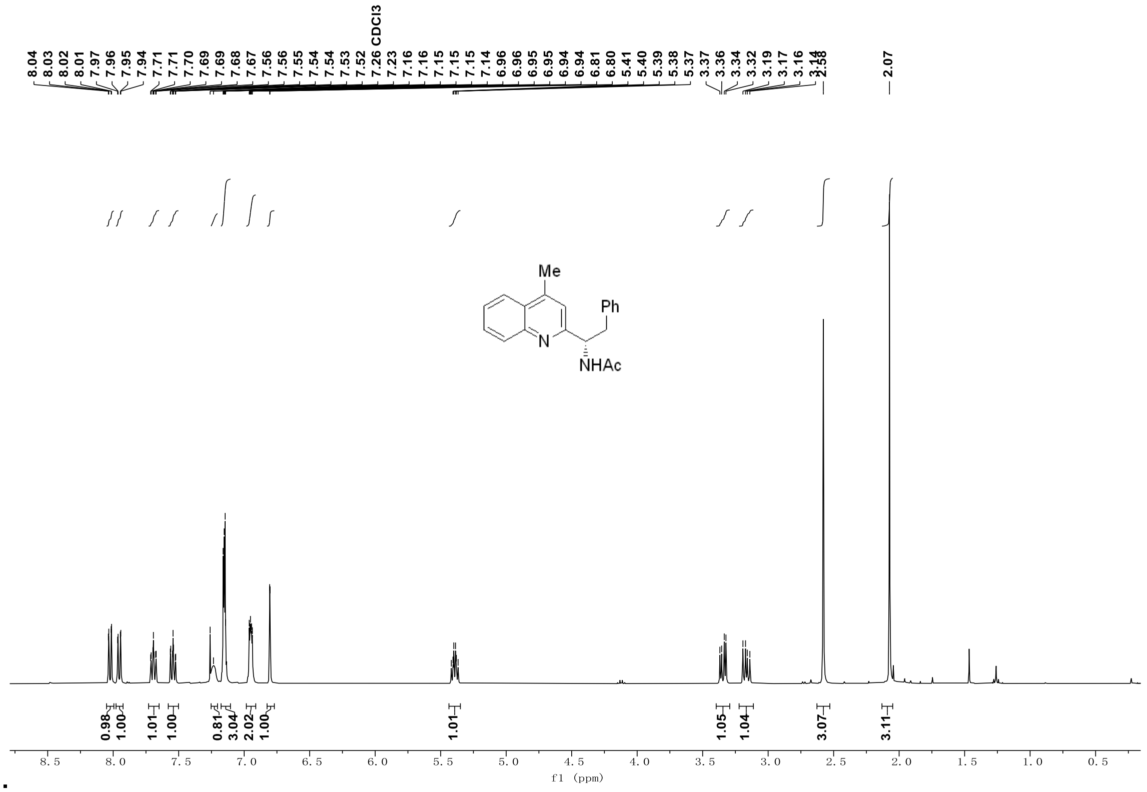

Supplementary Figure 76. ^{1}H NMR spectrum of (*S*)-N-(1-(4-methylquinolin-2-yl)-2-phenylethyl)acetamide.

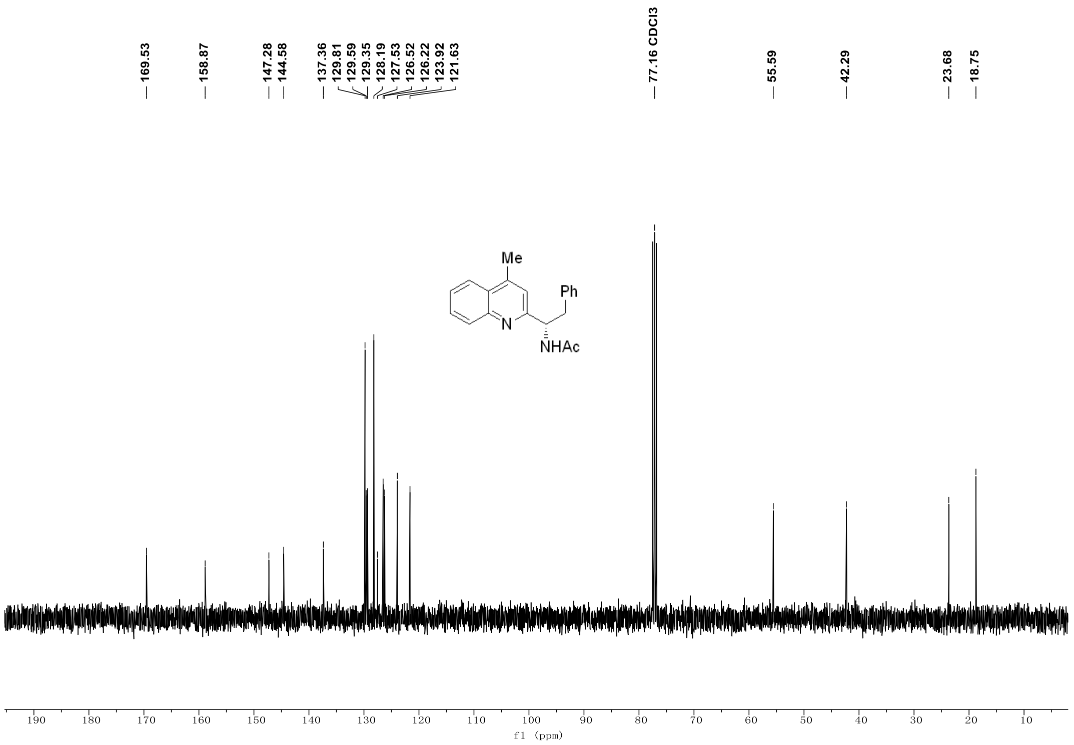

Supplementary Figure 77. ^{13}C NMR spectrum of (*S*)-N-(1-(4-methylquinolin-2-yl)-2-phenylethyl)acetamide.

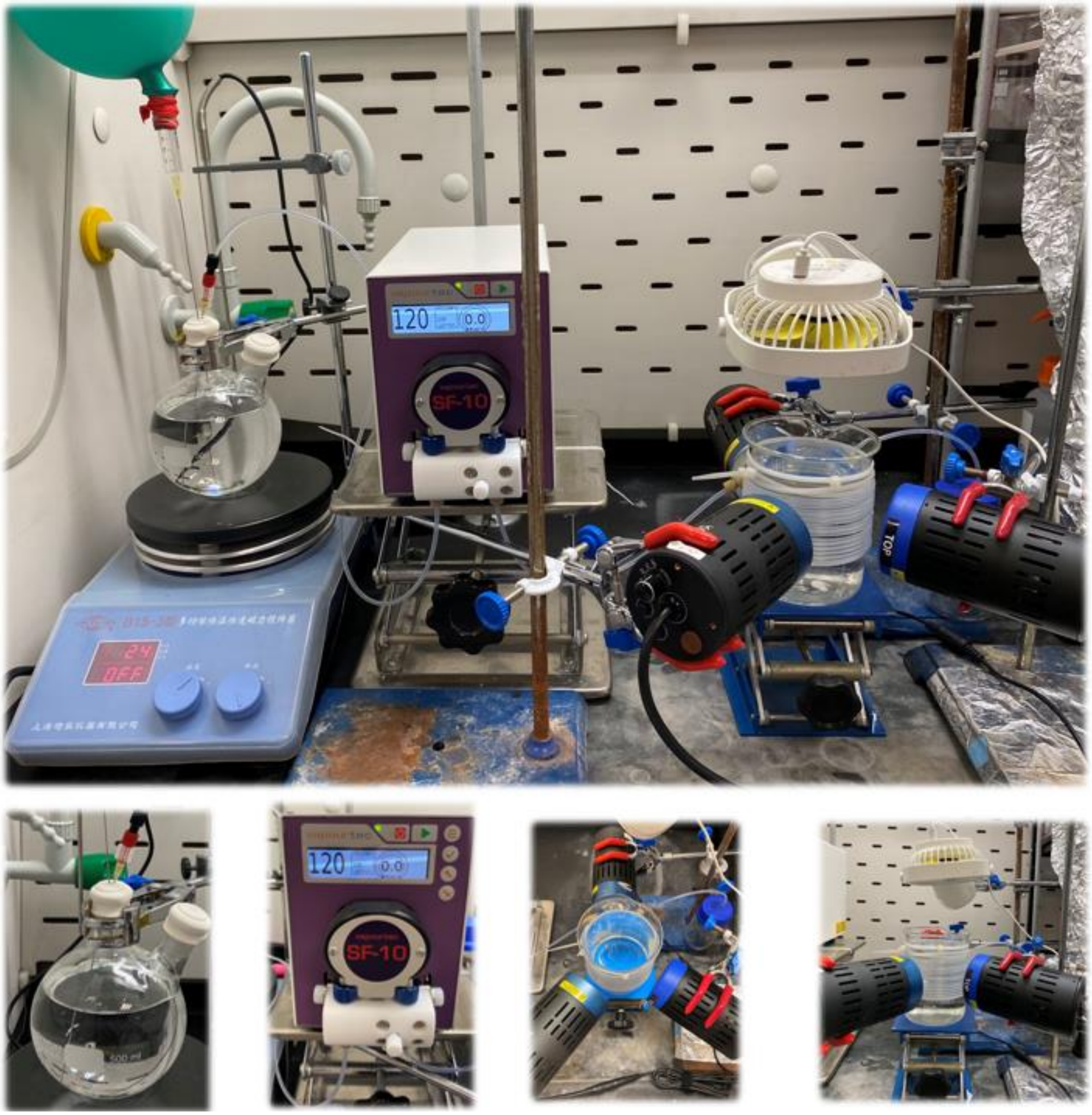

Supplementary Figure 78. Flow setup for the NaDT-catalyzed C-H fluorination reaction.

Note: The HPFA (high purity perfluoroalkoxyalkane) tubing (O.D. = 1/8 inch, I.D. = 1/16 inch, length = 3 m, volume = 6 mL) was rounded on a glass cylinder. The flow apparatus was cooled by a fan. The flow apparatus itself was set up with a residence time (t_r) of 50 min and a flow rate of 120 μL/min.

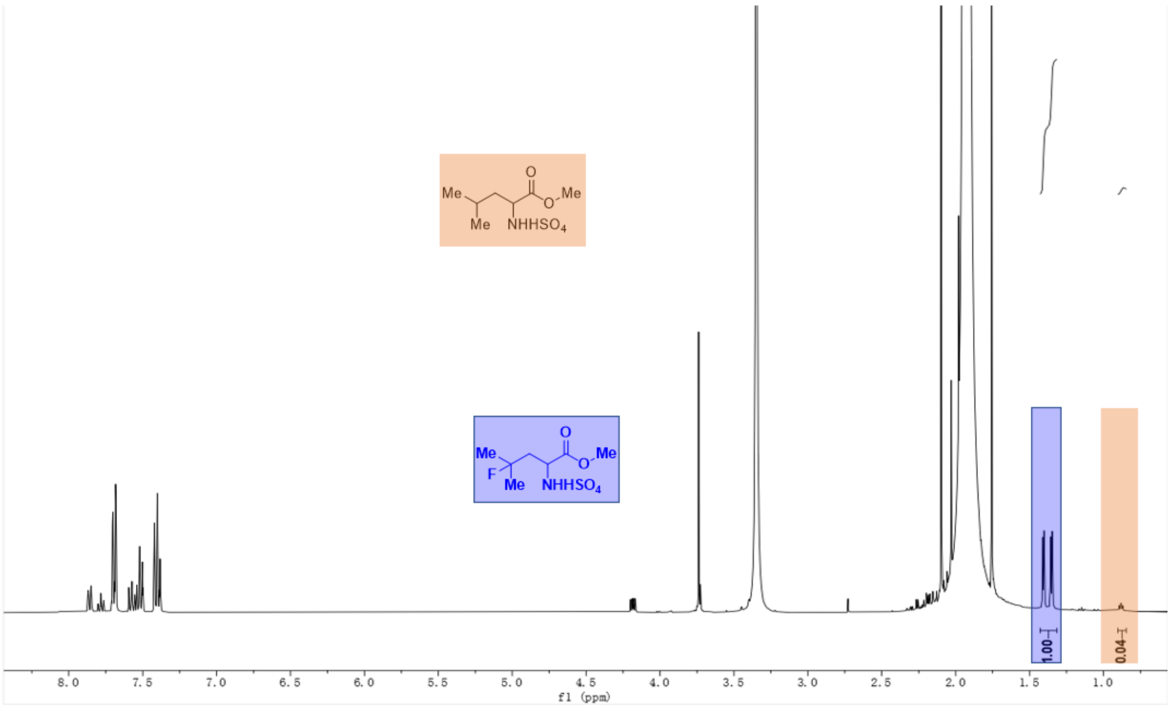

Supplementary Figure 79. Crude ^{1}H NMR spectrum for the NaDT-catalyzed C-H fluorination reaction.

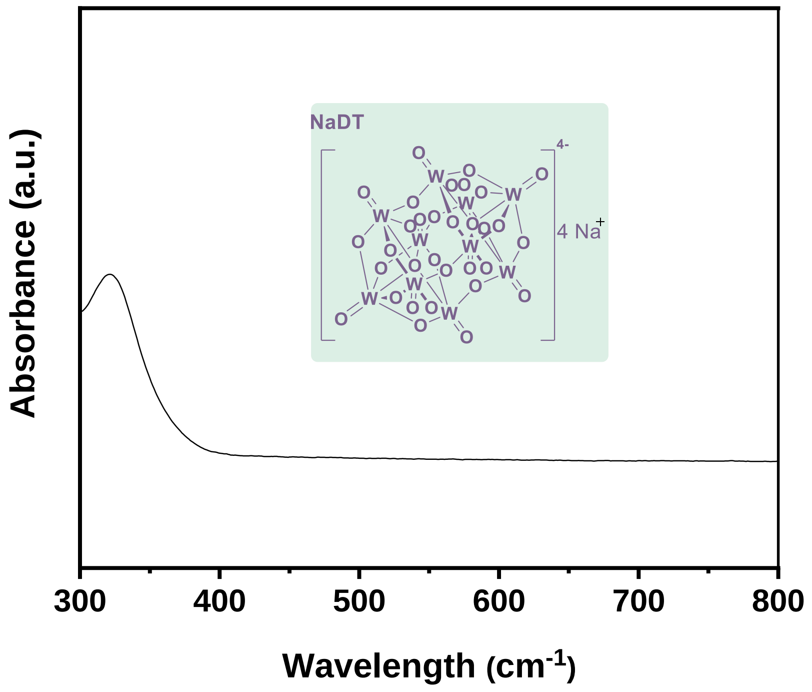

Supplementary Figure 80. UV-Vis spectra of the recovered NaDT for the C-H fluorination.

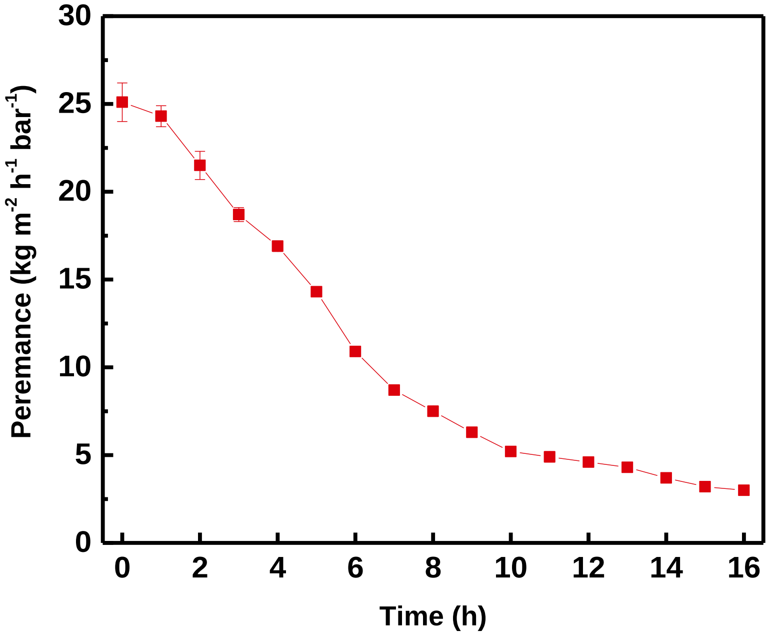

Supplementary Figure 81. Permeance of catalyst recovery as a function of time for the C-H fluorination. Error bars represent standard deviations for 3 measurements.

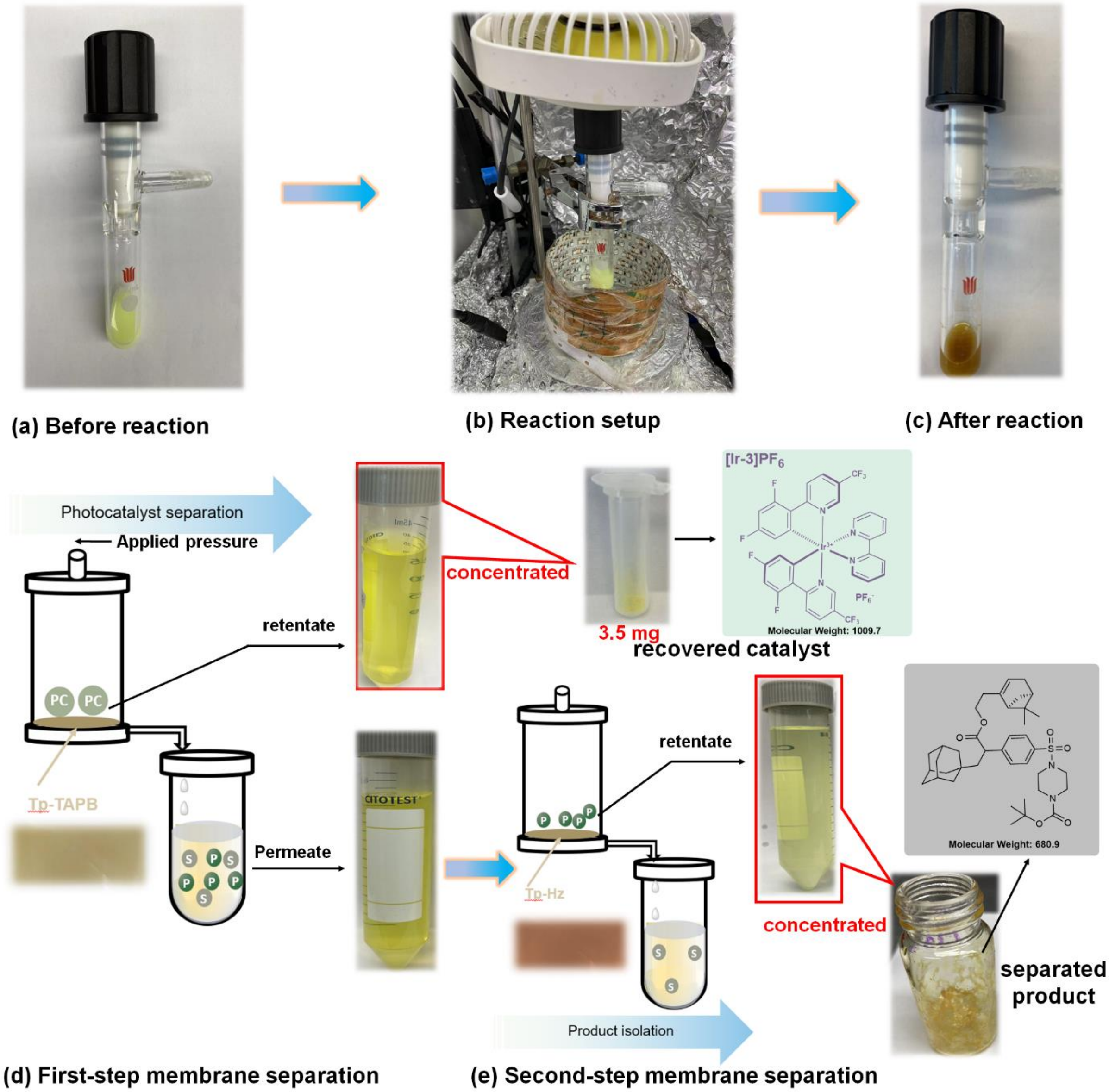

Supplementary Figure 82. Procedure for the three-component olefin difunctionalization and stepwise separation of photocatalysts and products. (a-c) Photos of the photocatalysis reaction. (d) Schematic of the first-step membrane separation for photocatalyst recovery. (e) Schematic of the second-step membrane separation for product separation.

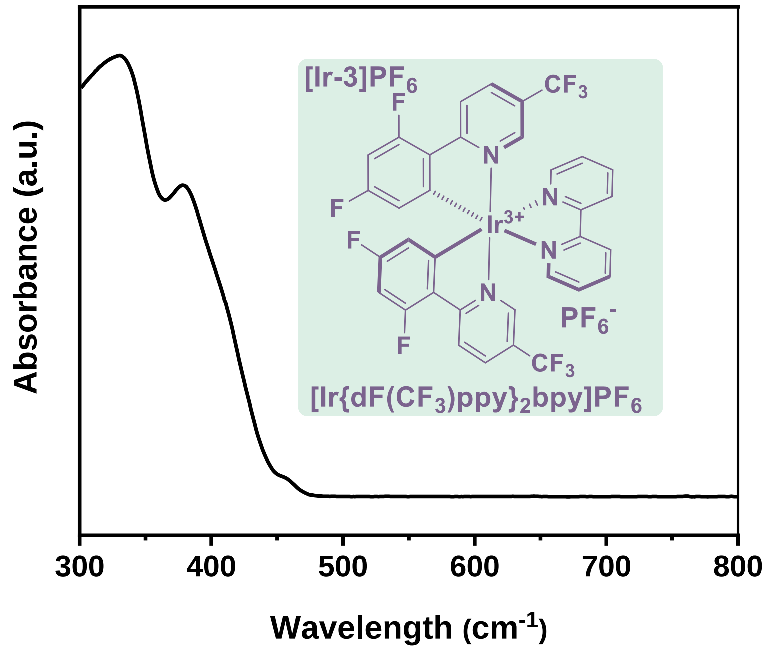

Supplementary Figure 83. UV-Vis spectra of the recovered [Ir-3]PF_6 catalyst for the three-component olefin difunctionalization.

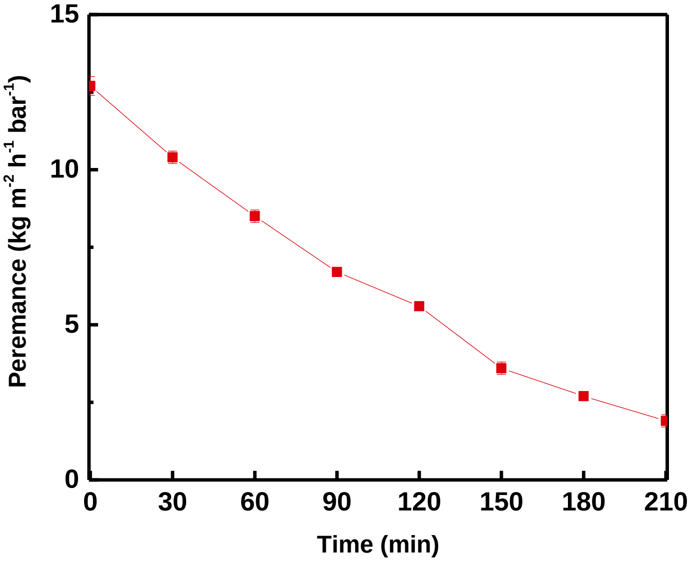

Supplementary Figure 84. Permeance of catalyst recovery as a function of time for the three-component olefin difunctionalization. Error bars represent standard deviations for 3 measurements.

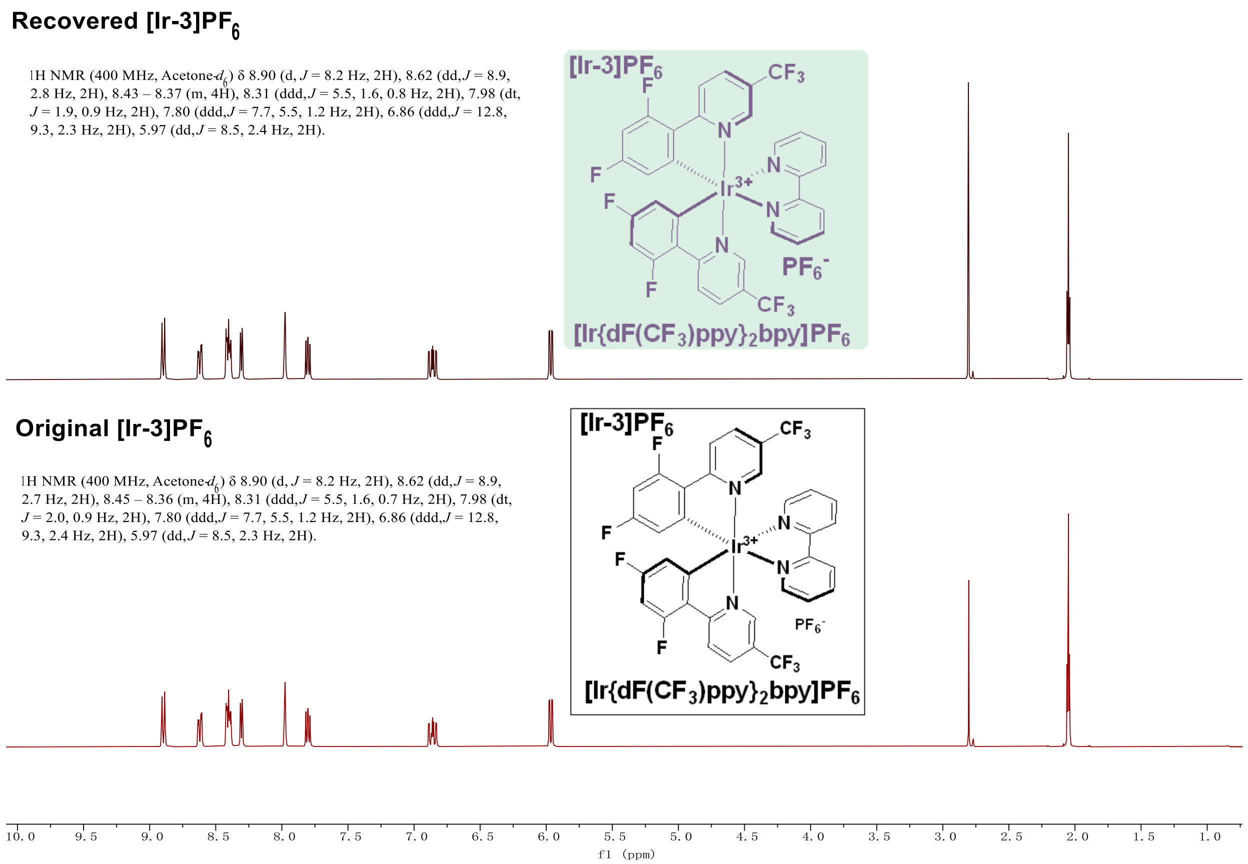

Supplementary Figure 85. ^{1}H NMR spectra of original and recovered photocatalyst [Ir-3]PF_6 for the three-component olefin difunctionalization.

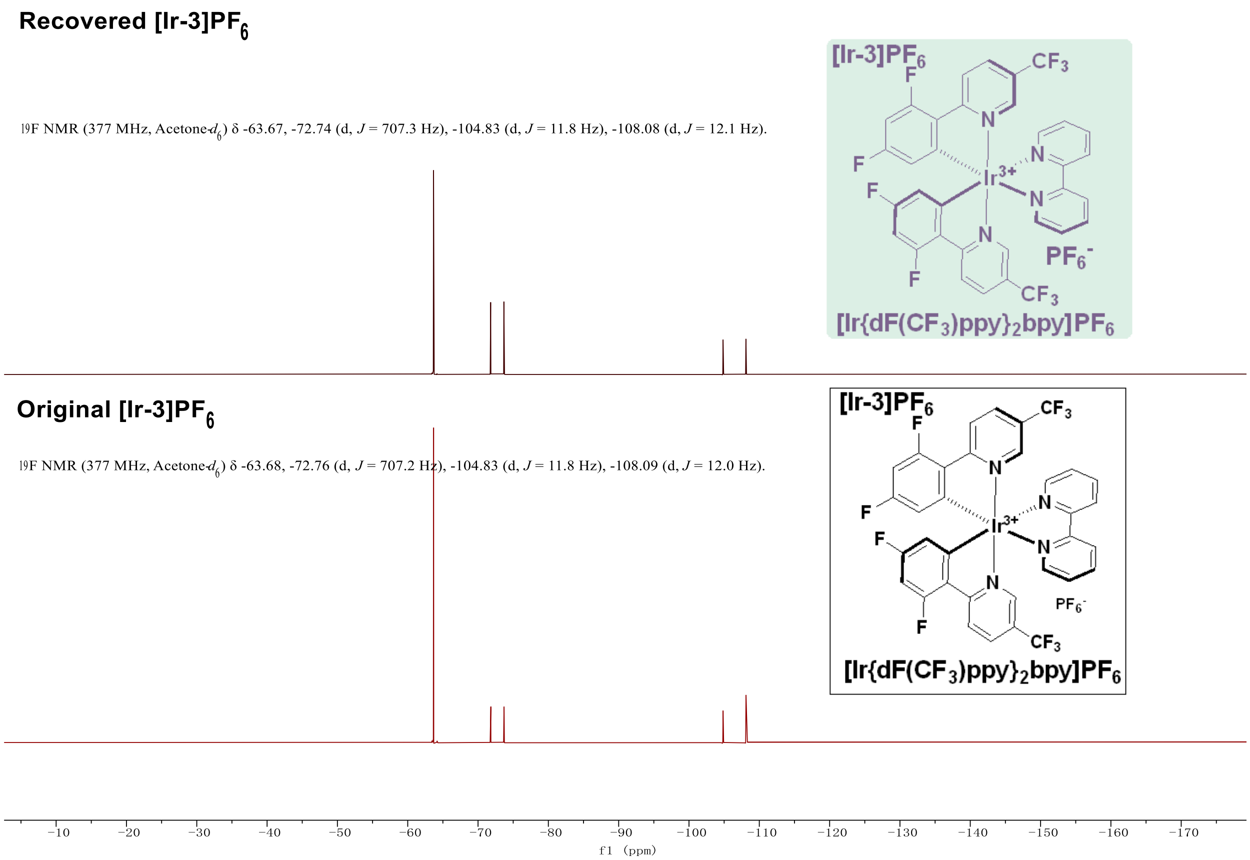

Supplementary Figure 86. ^{19}F NMR spectra of original and recovered photocatalyst [Ir-3]PF_6 for the three-component olefin difunctionalization.

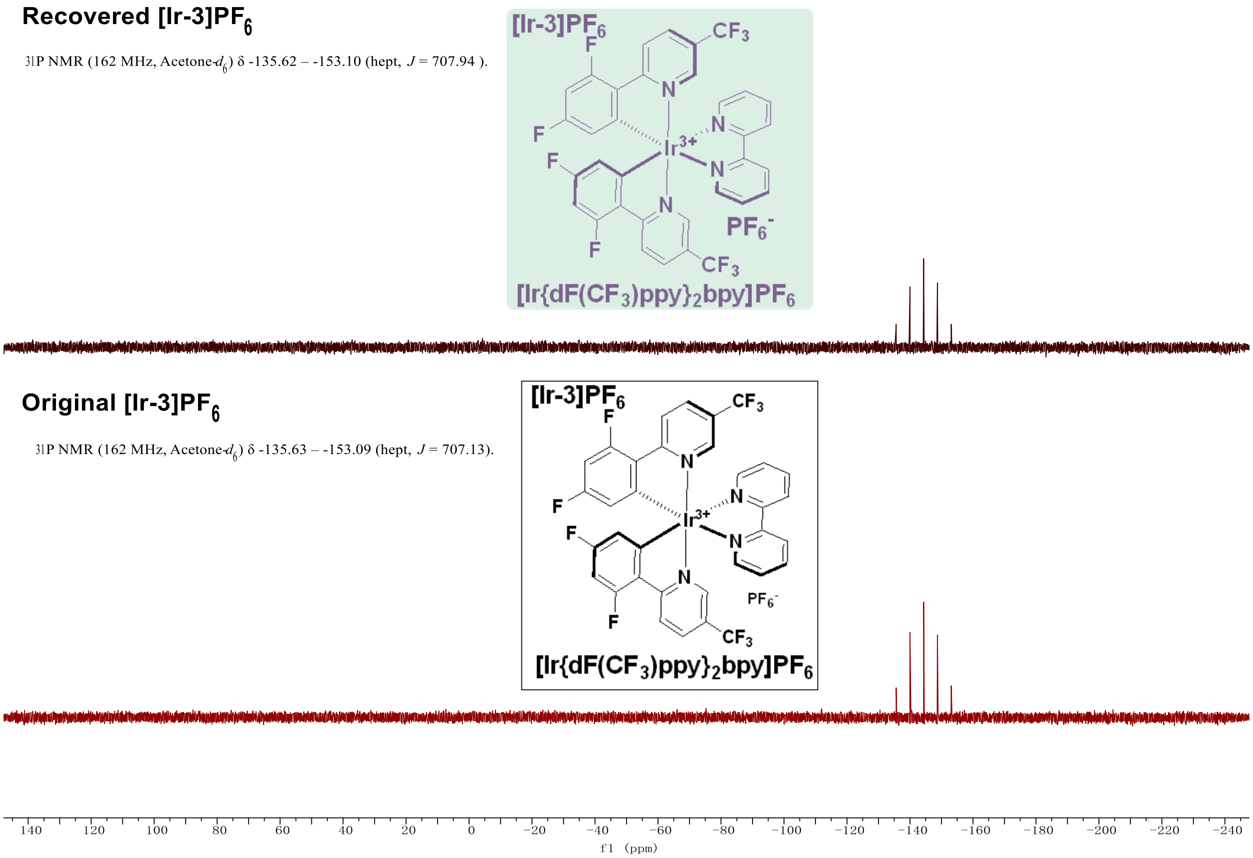

Supplementary Figure 87. ^{31}P NMR spectra of original and recovered photocatalyst [Ir-3]PF_6 for the three-component olefin difunctionalization.

Note: After nanofiltration, the recovered catalyst **[Ir-3]PF_6** was dried under vacuum and analyzed by NMR in acetone-*d*$_6$. The commercial photocatalyst **[Ir-3]PF_6** was used as the original **[Ir-3]PF_6** to compare with the recovered photocatalysts. As shown in **Supplementary Figs. 85-87**, all the spectra, including ^{1}H, ^{19}F and ^{31}P NMR of the recovered catalyst **[Ir-3]PF_6**, were consistent with the original ones, indicating the high purity of the recovered noble metal catalyst with no degradation.

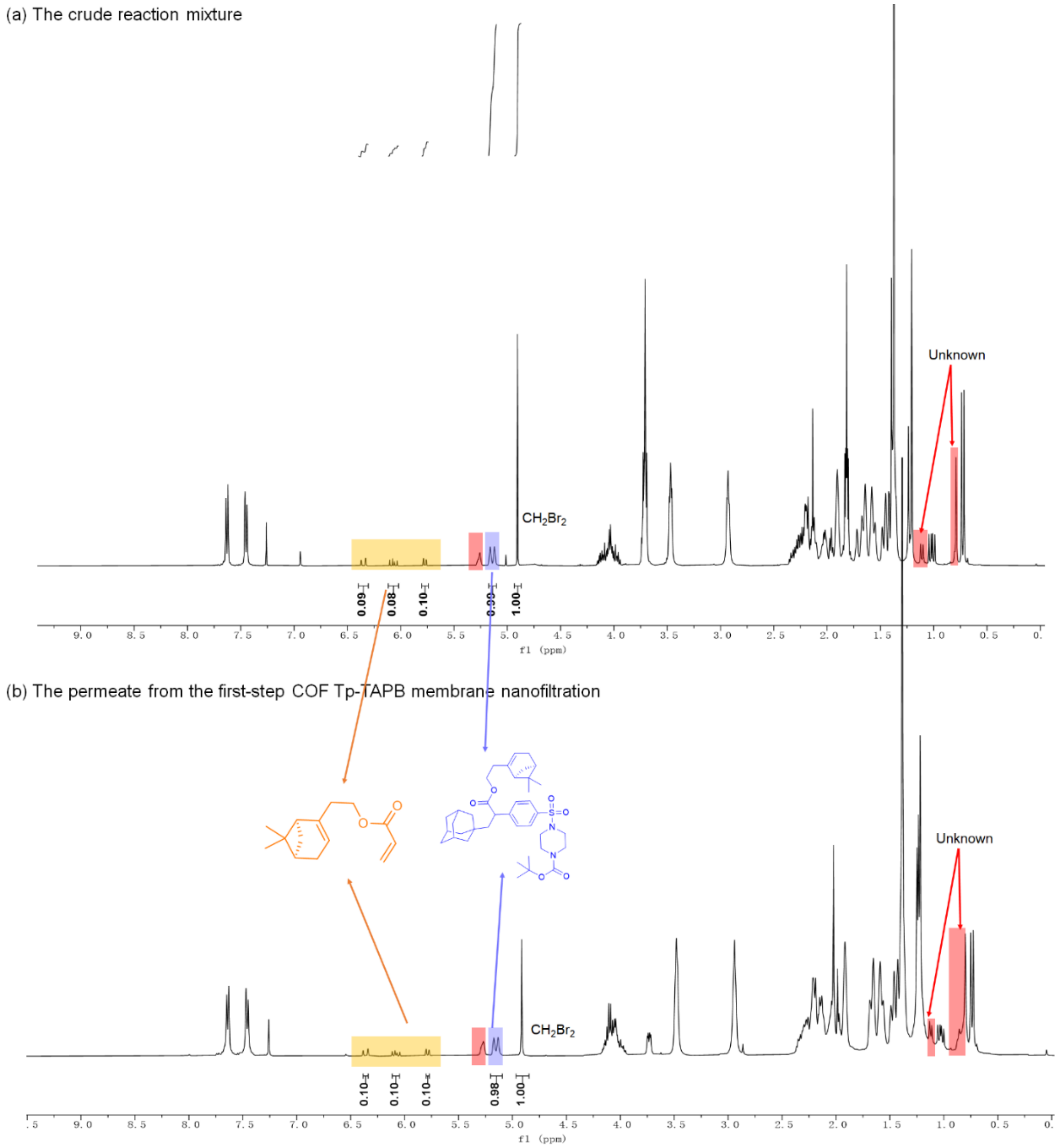

Supplementary Figure 88. ^1H NMR spectra for the three-component olefin difunctionalization using CH_2Br_2 as an internal standard: (a) the crude reaction mixture and (b) the permeate from the first-step COF Tp-TAPB membrane nanofiltration.

Note: As shown in the crude ^1H NMR spectra **Supplementary Fig. 88a**, the three-component olefin difunctionalization reaction performed well. The **[Ir-3]PF$_6$** photocatalyst was successfully recovered by a COF Tp-TAPB membrane (**Supplementary Figs. 85-87**). After recovering the **[Ir-3]PF$_6$**, the ^1H NMR spectrum of permeate solution (**Supplementary Fig. 88b**) was almost the same as the crude mixture, indicating that the starting materials and products, as well as some unknown components, were permeated through the COF Tp-TAPB membrane. The resulting permeate solution was further filtered through a COF Tp-HZ membrane to selectively separate the products from the reactant residues (**Supplementary Fig. 89)**.

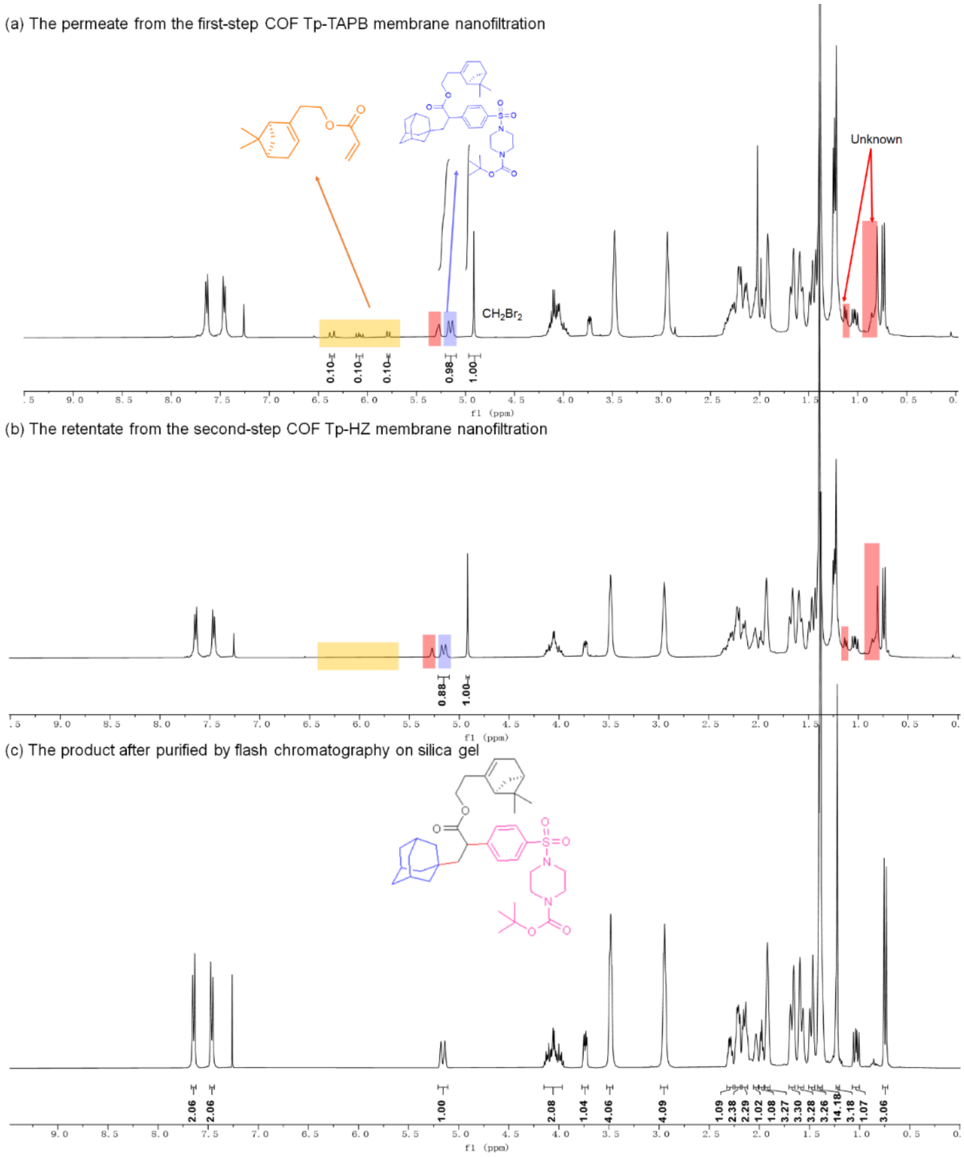

Supplementary Figure 89. ^{1}H NMR spectra of (a) the permeate from the first-step COF Tp-TAPB membrane nanofiltration, (b) the retentate from the second-step COF Tp-HZ membrane nanofiltration, and (c) the product purified by flash chromatography over silica gel.

Note: After the first-step COF Tp-TAPB membrane nanofiltration, the permeate was subsequently subjected to COF Tp-HZ membrane to isolate the desired product from the remaining starting materials and nickel catalysts with a 90% yield. Compared with **Supplementary Fig. 89a-b**, the remaining starting material of 2-((1*S*,5*R*)-6,6-dimethylbicyclo[3.1.1]hept-2-en-2-yl)ethyl acrylate was successfully separated from the product by COF Tp-HZ membrane nanofiltration. However, compared with the product purified by flash chromatography over silica gel (**Supplementary Fig. 89c**), the product separated from the second-step COF Tp-HZ membrane nanofiltration contained some unknown impurities in ^{1}H NMR spectra. The purity of these three solutions was further analyzed with analytic HPLC, as shown in **Supplementary Fig. 90**.

(a) The permeate from the first-step COF Tp-TAPB membrane nanofiltration

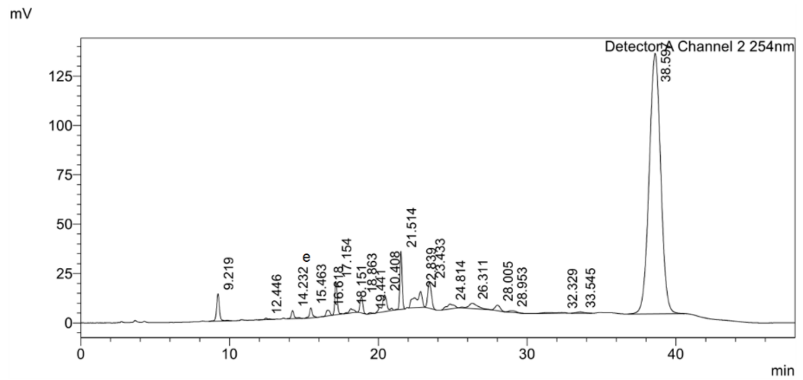

Detector A Channel 2 254nm

Peak#	Ret. Time	Area	Height	Conc.
1	9.219	185830	13773	2.020
2	12.446	13250	734	0.144
3	14.232	52758	3952	0.574
4	15.463	69209	5006	0.752
5	16.618	50050	2838	0.544
6	17.154	186595	16684	2.029
7	18.151	49422	2218	0.537
8	18.863	109799	8098	1.194
9	19.441	6174	501	0.067
10	20.408	201665	8504	2.193
11	21.514	305119	29354	3.317
12	22.839	268023	7995	2.914
13	23.433	224561	13164	2.441
14	24.814	85374	2421	0.928
15	26.311	119002	2695	1.294
16	28.005	66734	2785	0.726
17	28.953	27717	794	0.301
18	32.329	30033	334	0.327
19	33.545	24781	677	0.269
20	38.597	7121690	132013	77.428
Total		9197787	254539	

(b) The retentate from the second-step COF Tp-HZ membrane nanofiltration

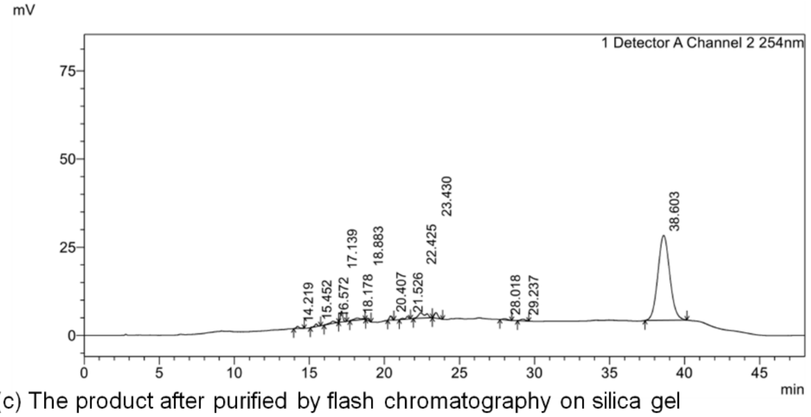

Detector A Channel 2 254nm

Peak#	Ret. Time	Area	Height	Conc.
1	14.219	7984	605	0.519
2	15.452	9722	720	0.632
3	16.572	13216	613	0.860
4	17.139	33493	3084	2.178
5	18.178	18133	536	1.179
6	18.883	5507	631	0.358
7	20.407	12706	1158	0.826
8	21.526	13591	766	0.884
9	22.425	52389	1500	3.407
10	23.430	25364	1563	1.650
11	28.018	7397	316	0.481
12	29.237	10473	475	0.681
13	38.603	1327606	24057	86.344
Total		1537580	36025	

(c) The product after purified by flash chromatography on silica gel

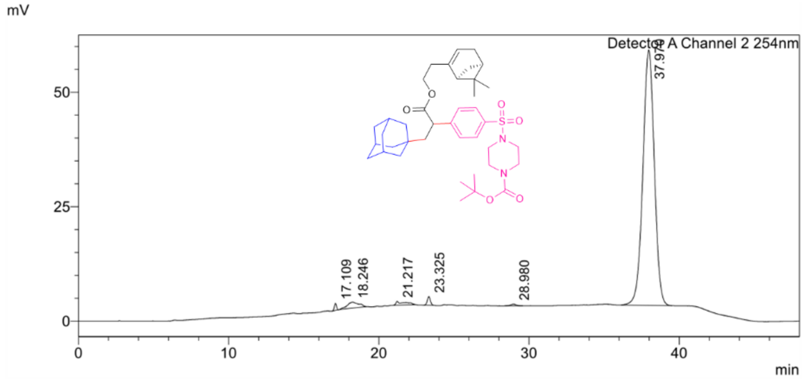

Detector A Channel 2 254nm

Peak#	Ret. Time	Area	Height	Conc.
1	17.109	14926	1490	0.479
2	18.246	70291	1292	2.257
3	21.217	33947	779	1.090
4	23.325	26725	1848	0.858
5	28.980	9832	359	0.316
6	37.970	2958602	55784	95.000
Total		3114324	61552	

Supplementary Figure 90. Analytic HPLC results of (a) the permeate from the first-step COF Tp-TAPB membrane nanofiltration, (b) the retentate from the second-step COF Tp-HZ membrane nanofiltration, and (c) the product purified by flash chromatography over silica gel.

Note: After the second-step COF Tp-HZ membrane nanofiltration, the product purity was improved from 77% (**Supplementary Fig. 90a**) to 86% (**Supplementary Fig. 90b**) based on HPLC analysis, but still lower than the product purified by flash chromatography over silica gel (95%, **Supplementary Fig. 90c**). Analytic HPLC was performed using a Shimadzu Prominence System equipped with a Welch Ultimate@ XB-C18 column (10 uM, 250 mm x 4.60 mm i.d.) in a mixed solvent system of water and methanol (total flow rate = 1.0 mL/min, 30 °C). A low-pressure gradient program was used as shown in **Supplementary Fig. 91.**

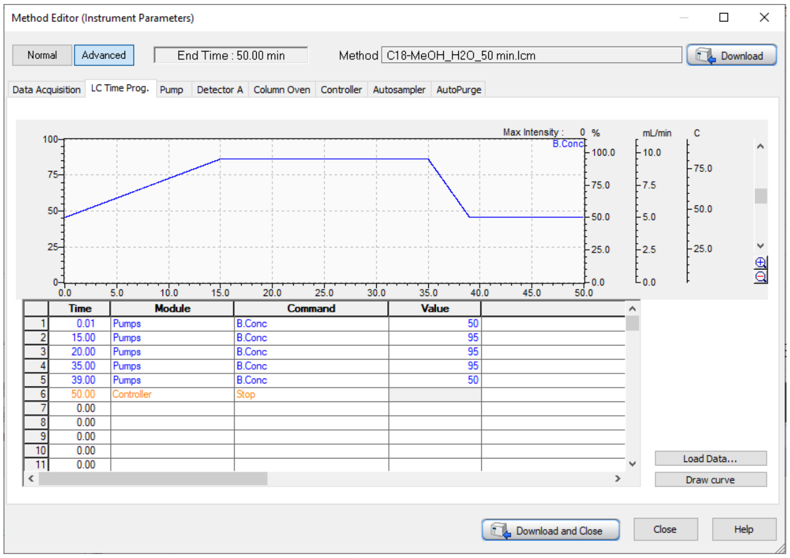

Supplementary Figure 91. A low-pressure gradient program used in analytic HPLC (B.Con is the concentration of methanol in water (v/v)).

To identify the unknown compounds, which induced the impurity of product, we isolated it by prepared TLC. The structure of the byproduct was characterized by NMR (**Supplementary Figs. 94** and **95**) and HRMS.

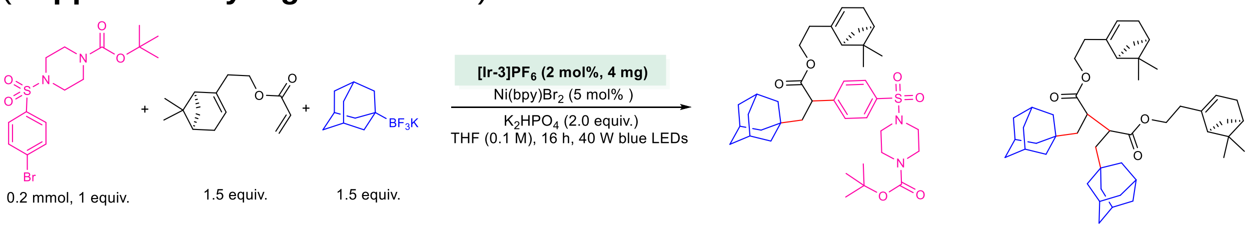

Chemical Formula: $C_{39}H_{56}N_2O_6S$
Molecular Weight: 680.4
Calculated $[M+Na]^+$: 703.3751
Found $[M+Na]^+$: 703.3750

product

Chemical Formula: $C_{48}H_{70}O_4$
Molecular Weight: 711.0840
Calculated $[M+Na]^+$: 733.5166
Found $[M+Na]^+$: 733.5165

byproduct

Due to the very similar molecular weight between that of the product and that of the byproduct (680.4 *vs.* 711.1), separation using COF Tp-HZ membrane nanofiltration is unsuccessful at the current stage. However, membrane separation proved very effective in separating the product from small molecule starting materials.

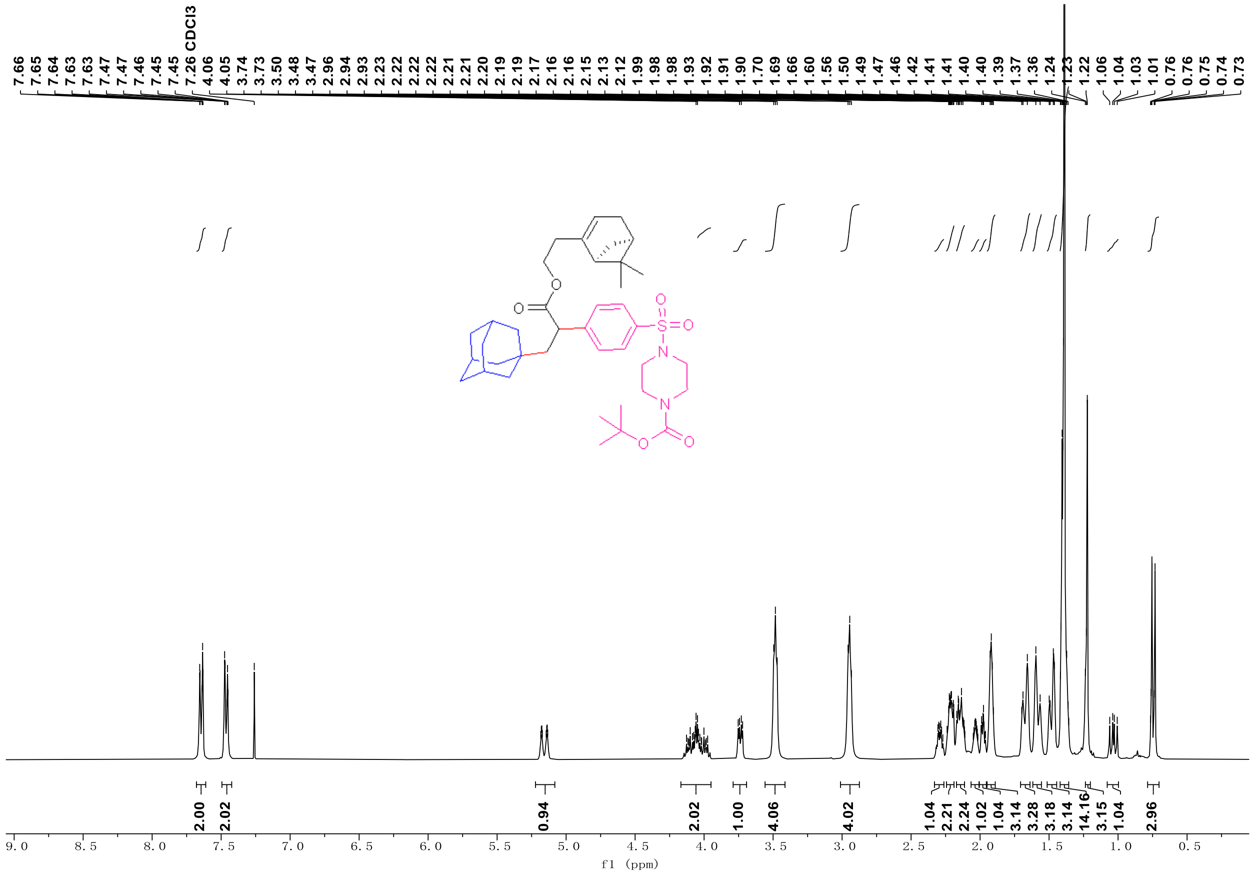

Supplementary Figure 92. ^{1}H NMR spectrum of tert-butyl 4-((4-(3-((3*S*,5*S*,7*S*)-adamantan-1-yl)-1-(2-((1*S*,5*R*)-6,6-dimethylbicyclo[3.1.1]hept-2-en-2-yl)ethoxy)-1-oxopropan-2-yl)phenyl)sulfonyl)piperazine-1-carboxylate.

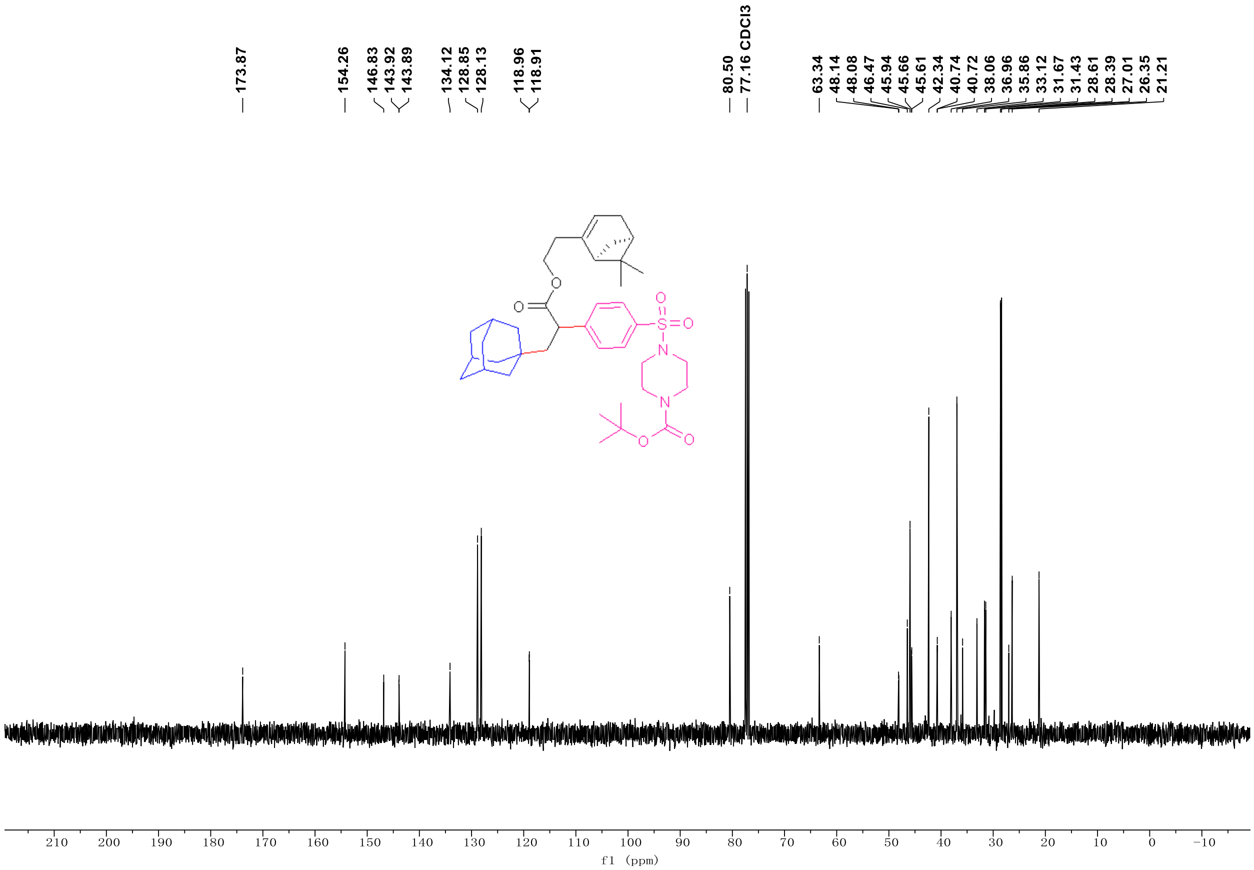

Supplementary Figure 93. ^{13}C NMR spectrum of tert-butyl 4-((4-(3-((3*S*,5*S*,7*S*)-adamantan-1-yl)-1-(2-((1*S*,5*R*)-6,6-dimethylbicyclo[3.1.1]hept-2-en-2-yl)ethoxy)-1-oxopropan-2-yl)phenyl)sulfonyl)piperazine-1-carboxylate.

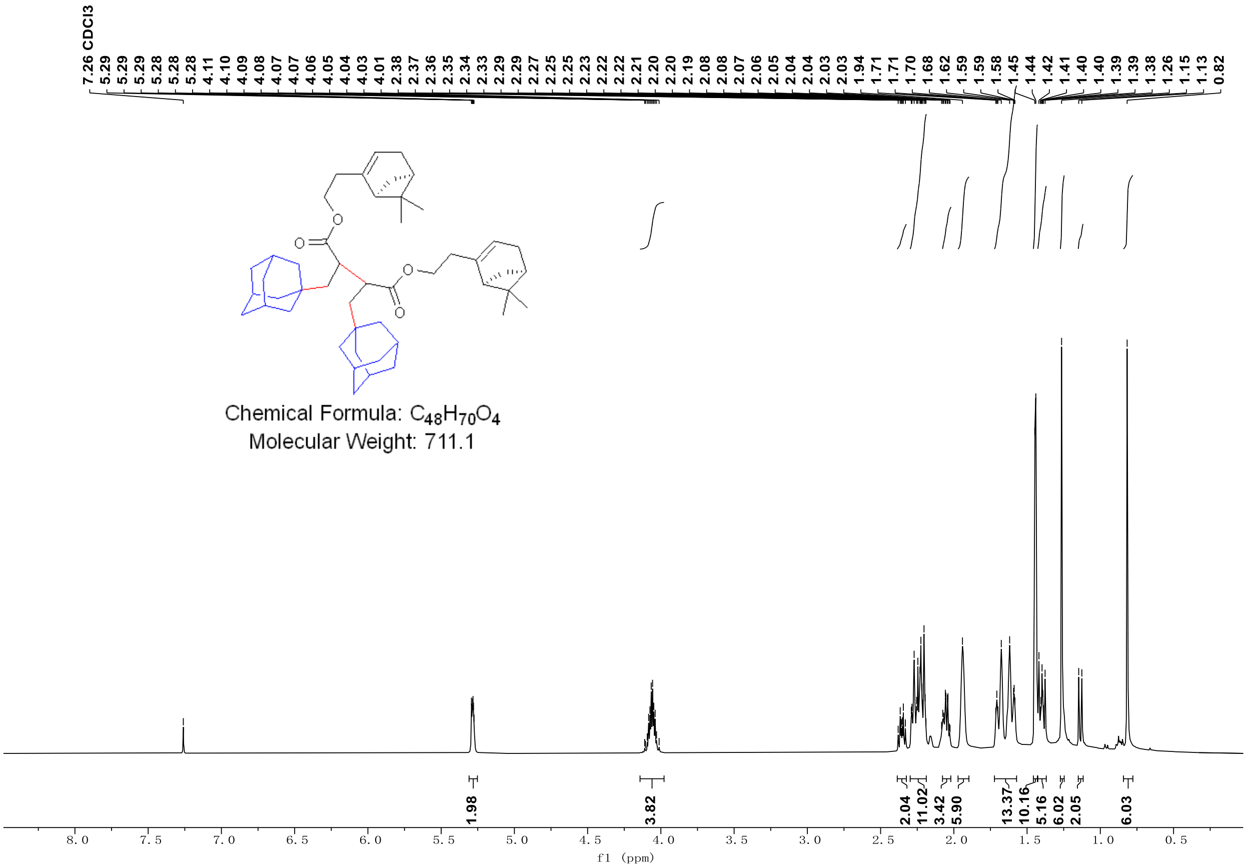

Supplementary Figure 94. ^{1}H NMR spectrum of byproduct bis(2-((1*S*,5*R*)-6,6-dimethylbicyclo[3.1.1]hept-2-en-2-yl)ethyl) 2,3-bis(((3*S*,5*S*,7*S*)-adamantan-1-yl)methyl)succinate.

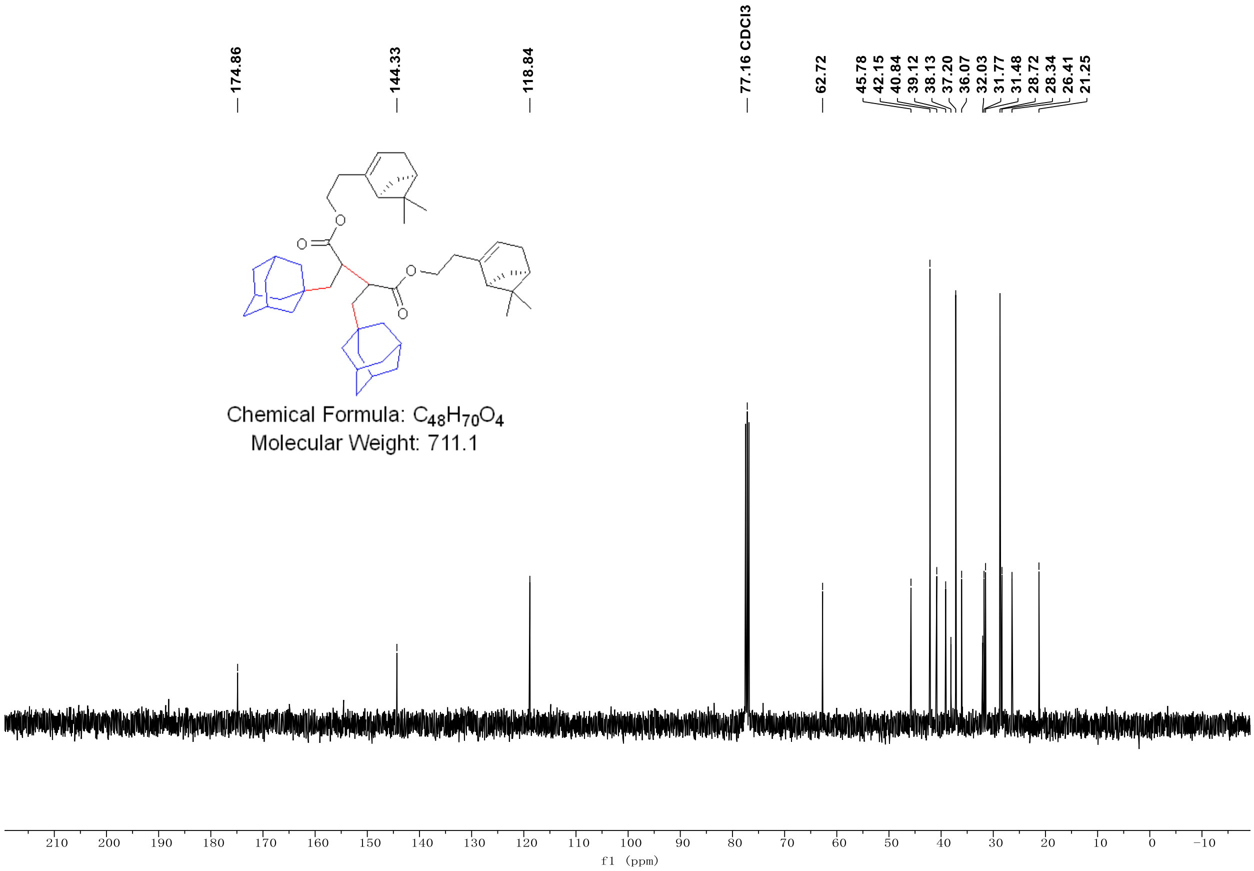

Supplementary Figure 95. ^{13}C NMR spectrum of byproduct bis(2-((1*S*,5*R*)-6,6-dimethylbicyclo[3.1.1]hept-2-en-2-yl)ethyl) 2,3-bis(((3*S*,5*S*,7*S*)-adamantan-1-yl)methyl)succinate.

X-Ray Crystallographic Data for NaDT

The single-crystal XRD data set for NaDT is deposited at the Cambridge Crystallographic Data Center (CCDC 2189883).

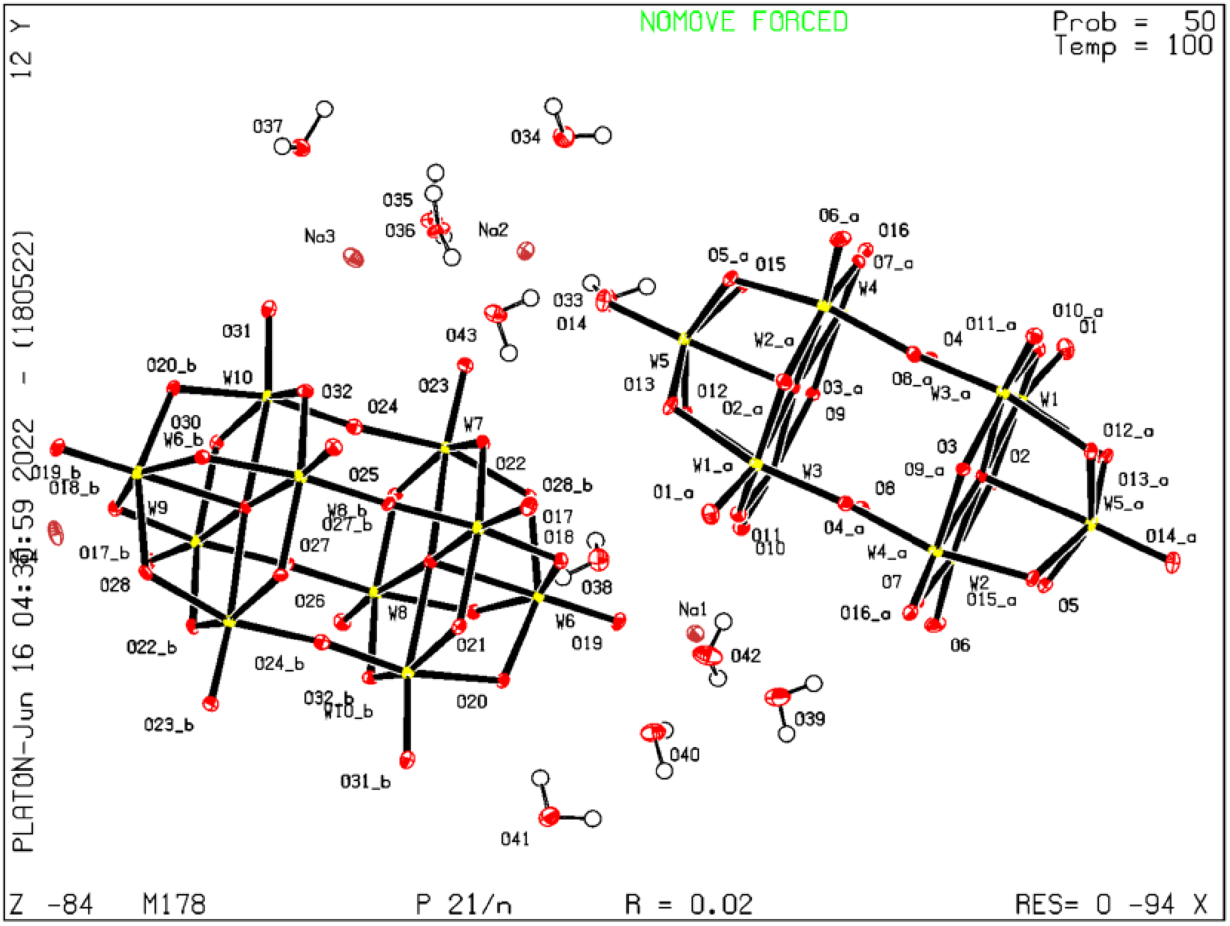

Supplementary Figure 96. Molecular structure of NaDT derived by single crystal XRD.

Supplementary Table 8. Crystal data and structure refinement for NaDT.

Identification code	NaDT
Empirical formula	$H_{22}Na_4O_{43}W_{10}$
Formula weight	2640.63
Temperature	100(2) K
Wavelength	0.71073 Å
Crystal system	Monoclinic
Space group	$P2_1/n$
Unit cell dimensions	a = 11.4274(4) Å, a= 90°.<br>b = 15.8405(5) Å, b= 105.5440(10)°.<br>c = 21.0258(8) Å, g = 90°.
Volume	3666.8(2) Å^3
Z	4
Density (calculated)	4.783 Mg/m^3
Absorption coefficient	31.403 mm^{-1}
F(000)	4600
Crystal size	0.044 x 0.073 x 0.114mm^3
Theta range for data collection	2.256 to 30.566°.
Index ranges	-16<=h<=16, -21<=k<=22, -30<=l<=30
Reflections collected	172686
Independent reflections	11200 [R(int) = 0.0726]
Completeness to theta = 25.242°	99.8 %
Absorption correction	Semi-empirical from equivalents
Max. and min. transmission	0.7461 and 0.4075
Refinement method	Full-matrix least-squares on F^2
Data / restraints / parameters	11200 / 4 / 580
Goodness-of-fit on F^2	1.054
Final R indices [I>2sigma(I)]	R1 = 0.0203, wR2 = 0.0379
R indices (all data)	R1 = 0.0273, wR2 = 0.0396
Extinction coefficient	n/a
Largest diff. peak and hole	1.178 and -1.503 e.Å^{-3}

Supplementary Table 9. Atomic coordinates (×10^4) and equivalent isotropic displacement parameters ($Å^2$×10^3) for NaDT. U(eq) is defined as one third of the trace of the orthogonalized U^{ij} tensor.

	x	y	z	U(eq)
W(1)	11084(1)	4104(1)	11238(1)	6(1)
W(2)	8181(1)	4497(1)	10733(1)	6(1)
W(3)	8362(1)	3912(1)	9026(1)	5(1)
W(4)	11258(1)	3519(1)	9524(1)	6(1)
W(5)	10100(1)	4385(1)	8061(1)	7(1)
W(6)	5021(1)	4337(1)	6950(1)	6(1)
W(7)	6756(1)	3868(1)	5980(1)	5(1)
W(8)	3854(1)	3490(1)	5477(1)	5(1)
W(9)	3801(1)	4152(1)	3745(1)	6(1)
W(10)	6702(1)	4532(1)	4246(1)	5(1)
Na(1)	4980(2)	2999(1)	8507(1)	11(1)
Na(2)	10213(2)	3023(1)	6574(1)	12(1)
Na(3)	9064(2)	3036(1)	4983(1)	13(1)
Na(4)	2578(2)	2338(1)	2516(1)	12(1)
O(1)	11941(3)	3292(2)	11653(2)	12(1)
O(2)	9451(2)	3702(2)	11075(2)	9(1)
O(3)	9921(2)	5298(2)	10892(1)	6(1)
O(4)	11118(2)	3881(2)	10381(1)	8(1)
O(5)	8432(2)	4931(2)	11608(1)	8(1)
O(6)	6852(3)	3965(2)	10671(2)	12(1)
O(7)	7569(2)	5584(2)	10404(1)	6(1)
O(8)	8374(2)	4259(2)	9870(1)	8(1)
O(9)	9634(2)	3115(2)	9362(1)	7(1)
O(10)	7082(3)	3310(2)	8818(2)	9(1)
O(11)	7752(2)	5006(2)	8690(1)	8(1)
O(12)	8750(2)	3767(2)	8163(1)	7(1)
O(13)	9221(3)	5394(2)	7939(1)	9(1)
O(14)	10176(3)	4127(2)	7282(2)	11(1)
O(15)	11102(2)	3490(2)	8599(1)	8(1)
O(16)	12167(2)	2656(2)	9741(1)	10(1)

O(17)	6428(2)	3713(2)	6812(1)	7(1)
O(18)	5998(2)	5302(2)	7072(1)	7(1)
O(19)	5001(3)	4033(2)	7729(1)	10(1)
O(20)	3603(2)	4986(2)	6664(1)	7(1)
O(21)	4111(2)	3409(2)	6409(1)	8(1)
O(22)	7370(2)	4976(2)	6261(1)	7(1)
O(23)	8058(2)	3291(2)	6108(2)	9(1)
O(24)	6649(2)	4256(2)	5089(1)	8(1)
O(25)	5481(2)	3106(2)	5591(1)	7(1)
O(26)	2937(2)	2638(2)	5240(2)	10(1)
O(27)	3888(2)	3899(2)	4618(1)	7(1)
O(28)	2926(3)	3340(2)	3342(2)	11(1)
O(29)	4979(2)	5329(2)	4100(1)	6(1)
O(30)	5423(2)	3753(2)	3855(1)	6(1)
O(31)	7984(3)	4007(2)	4198(2)	9(1)
O(32)	7319(2)	5636(2)	4531(1)	7(1)
O(33)	9650(3)	2143(2)	7406(2)	16(1)
O(34)	12203(3)	2642(3)	6841(2)	22(1)
O(35)	9860(3)	2004(2)	5724(2)	12(1)
O(36)	10304(3)	4012(2)	5697(2)	13(1)
O(37)	10522(3)	2960(2)	4395(2)	12(1)
O(38)	5373(3)	2176(2)	7582(2)	15(1)
O(39)	4746(3)	4097(2)	9273(2)	21(1)
O(40)	2976(3)	2527(2)	8231(2)	15(1)
O(41)	1889(3)	5804(2)	7182(2)	14(1)
O(42)	5442(3)	5682(2)	8502(2)	20(1)
O(43)	9703(3)	5515(2)	6227(2)	14(1)

Supplementary Table 10. Bond lengths [Å] and angles [°] for NaDT.

W(1)-O(1)	1.708(3)
W(1)-O(4)	1.847(3)
W(1)-O(2)	1.914(3)
W(1)-O(11)#1	1.916(3)
W(1)-O(13)#1	2.016(3)
W(1)-O(3)	2.314(3)
W(2)-O(6)	1.710(3)
W(2)-O(2)	1.909(3)
W(2)-O(5)	1.911(3)
W(2)-O(7)	1.917(3)
W(2)-O(8)	1.925(3)
W(2)-O(3)	2.307(3)
W(3)-O(10)	1.703(3)
W(3)-O(8)	1.853(3)
W(3)-O(9)	1.912(3)
W(3)-O(11)	1.929(3)
W(3)-O(12)	1.993(3)
W(3)-O(3)#1	2.294(3)
W(3)-Na(4)#2	3.6437(17)
W(4)-O(16)	1.703(3)
W(4)-O(9)	1.905(3)
W(4)-O(15)	1.905(3)
W(4)-O(7)#1	1.932(3)
W(4)-O(4)	1.938(3)
W(4)-O(3)#1	2.336(3)
W(5)-O(14)	1.712(3)
W(5)-O(13)	1.869(3)
W(5)-O(12)	1.888(3)
W(5)-O(5)#1	1.962(3)
W(5)-O(15)	1.977(3)
W(5)-O(3)#1	2.265(3)
W(6)-O(19)	1.714(3)
W(6)-O(18)	1.870(3)
W(6)-O(20)	1.877(3)

W(6)-O(17)	1.974(3)
W(6)-O(21)	1.975(3)
W(6)-O(29)#3	2.271(3)
W(7)-O(23)	1.706(3)
W(7)-O(17)	1.900(3)
W(7)-O(25)	1.900(3)
W(7)-O(22)	1.923(3)
W(7)-O(24)	1.945(3)
W(7)-O(29)#3	2.323(3)
W(7)-Na(4)#2	3.6516(17)
W(8)-O(26)	1.700(3)
W(8)-O(21)	1.907(3)
W(8)-O(25)	1.910(3)
W(8)-O(32)#3	1.924(3)
W(8)-O(27)	1.929(3)
W(8)-O(29)#3	2.331(3)
W(9)-O(28)	1.709(3)
W(9)-O(27)	1.855(3)
W(9)-O(30)	1.913(3)
W(9)-O(22)#3	1.920(3)
W(9)-O(18)#3	1.991(3)
W(9)-O(29)	2.303(3)
W(10)-O(31)	1.710(3)
W(10)-O(24)	1.841(3)
W(10)-O(32)	1.921(3)
W(10)-O(30)	1.921(3)
W(10)-O(20)#3	2.002(3)
W(10)-O(29)	2.290(3)
W(10)-Na(3)	3.6138(16)
Na(1)-O(19)	2.319(3)
Na(1)-O(40)	2.330(4)
Na(1)-O(37)#4	2.357(3)
Na(1)-O(10)	2.366(3)
Na(1)-O(39)	2.435(4)
Na(1)-O(38)	2.481(4)
Na(1)-Na(3)#4	3.893(2)

Na(1)-Na(4)#2	4.093(2)
Na(2)-O(34)	2.273(4)
Na(2)-O(14)	2.304(3)
Na(2)-O(35)	2.363(4)
Na(2)-O(23)	2.431(3)
Na(2)-O(36)	2.443(4)
Na(2)-O(33)	2.454(4)
Na(2)-Na(3)	3.250(3)
Na(2)-Na(4)#2	4.064(2)
Na(2)-H(34A)	2.63(6)
Na(3)-O(35)	2.272(4)
Na(3)-O(37)	2.328(4)
Na(3)-O(36)	2.349(4)
Na(3)-O(31)	2.350(3)
Na(3)-O(16)#5	2.359(3)
Na(3)-O(23)	2.921(3)
Na(4)-O(28)	2.307(3)
Na(4)-O(1)#6	2.325(3)
Na(4)-O(17)#5	2.376(3)
Na(4)-O(12)#5	2.393(3)
Na(4)-O(33)#5	2.575(4)
Na(4)-O(38)#5	2.674(4)
O(33)-H(33A)	0.84(4)
O(33)-H(33B)	0.85(4)
O(34)-H(34A)	0.73(7)
O(34)-H(34B)	0.83(6)
O(35)-H(35A)	0.82(6)
O(35)-H(35B)	0.93(5)
O(36)-H(36A)	0.90(5)
O(36)-H(36B)	0.75(5)
O(37)-H(37A)	0.87(4)
O(37)-H(37B)	0.88(4)
O(38)-H(38A)	0.80(6)
O(38)-H(38B)	0.76(6)
O(39)-H(39A)	0.72(4)
O(39)-H(39B)	0.73(4)

O(40)-H(40A)	0.78(4)
O(40)-H(40B)	0.77(4)
O(41)-H(41A)	0.82(6)
O(41)-H(41B)	0.84(6)
O(42)-H(42A)	0.75(6)
O(42)-H(42B)	0.85(6)
O(43)-H(43A)	0.83(6)
O(43)-H(43B)	0.78(6)

O(1)-W(1)-O(4)	101.26(13)
O(1)-W(1)-O(2)	103.83(13)
O(4)-W(1)-O(2)	92.03(12)
O(1)-W(1)-O(11)#1	102.92(13)
O(4)-W(1)-O(11)#1	91.27(12)
O(2)-W(1)-O(11)#1	151.84(11)
O(1)-W(1)-O(13)#1	93.77(13)
O(4)-W(1)-O(13)#1	164.96(12)
O(2)-W(1)-O(13)#1	84.30(12)
O(11)#1-W(1)-O(13)#1	85.32(12)
O(1)-W(1)-O(3)	168.14(12)
O(4)-W(1)-O(3)	90.56(11)
O(2)-W(1)-O(3)	76.41(10)
O(11)#1-W(1)-O(3)	75.60(10)
O(13)#1-W(1)-O(3)	74.41(10)
O(6)-W(2)-O(2)	105.94(13)
O(6)-W(2)-O(5)	98.78(13)
O(2)-W(2)-O(5)	88.44(12)
O(6)-W(2)-O(7)	100.58(13)
O(2)-W(2)-O(7)	153.46(12)
O(5)-W(2)-O(7)	88.29(12)
O(6)-W(2)-O(8)	98.91(13)
O(2)-W(2)-O(8)	87.58(12)
O(5)-W(2)-O(8)	162.28(12)
O(7)-W(2)-O(8)	87.59(12)
O(6)-W(2)-O(3)	174.42(12)
O(2)-W(2)-O(3)	76.68(11)

O(5)-W(2)-O(3)	76.23(11)
O(7)-W(2)-O(3)	76.97(10)
O(8)-W(2)-O(3)	86.06(11)
O(10)-W(3)-O(8)	101.45(13)
O(10)-W(3)-O(9)	103.92(12)
O(8)-W(3)-O(9)	91.82(12)
O(10)-W(3)-O(11)	102.00(12)
O(8)-W(3)-O(11)	89.65(12)
O(9)-W(3)-O(11)	153.19(12)
O(10)-W(3)-O(12)	94.92(13)
O(8)-W(3)-O(12)	163.54(12)
O(9)-W(3)-O(12)	85.86(12)
O(11)-W(3)-O(12)	85.23(12)
O(10)-W(3)-O(3)#1	169.54(12)
O(8)-W(3)-O(3)#1	88.83(11)
O(9)-W(3)-O(3)#1	77.39(10)
O(11)-W(3)-O(3)#1	75.87(10)
O(12)-W(3)-O(3)#1	74.76(10)
O(10)-W(3)-Na(4)#2	58.47(10)
O(8)-W(3)-Na(4)#2	158.74(9)
O(9)-W(3)-Na(4)#2	87.04(9)
O(11)-W(3)-Na(4)#2	100.86(9)
O(12)-W(3)-Na(4)#2	37.51(8)
O(3)#1-W(3)-Na(4)#2	111.55(7)
O(16)-W(4)-O(9)	105.89(12)
O(16)-W(4)-O(15)	97.92(13)
O(9)-W(4)-O(15)	89.43(12)
O(16)-W(4)-O(7)#1	102.04(12)
O(9)-W(4)-O(7)#1	152.03(11)
O(15)-W(4)-O(7)#1	88.29(12)
O(16)-W(4)-O(4)	100.64(13)
O(9)-W(4)-O(4)	86.92(12)
O(15)-W(4)-O(4)	161.38(11)
O(7)#1-W(4)-O(4)	86.42(12)
O(16)-W(4)-O(3)#1	173.15(12)
O(9)-W(4)-O(3)#1	76.46(10)

O(15)-W(4)-O(3)#1	75.56(11)
O(7)#1-W(4)-O(3)#1	75.98(10)
O(4)-W(4)-O(3)#1	85.84(10)
O(14)-W(5)-O(13)	103.68(13)
O(14)-W(5)-O(12)	103.76(13)
O(13)-W(5)-O(12)	91.87(12)
O(14)-W(5)-O(5)#1	102.37(13)
O(13)-W(5)-O(5)#1	87.33(12)
O(12)-W(5)-O(5)#1	153.28(12)
O(14)-W(5)-O(15)	101.93(13)
O(13)-W(5)-O(15)	154.07(12)
O(12)-W(5)-O(15)	85.91(12)
O(5)#1-W(5)-O(15)	83.33(11)
O(14)-W(5)-O(3)#1	177.60(12)
O(13)-W(5)-O(3)#1	78.33(11)
O(12)-W(5)-O(3)#1	77.38(11)
O(5)#1-W(5)-O(3)#1	76.31(10)
O(15)-W(5)-O(3)#1	75.97(10)
O(19)-W(6)-O(18)	105.02(13)
O(19)-W(6)-O(20)	103.25(13)
O(18)-W(6)-O(20)	91.64(11)
O(19)-W(6)-O(17)	102.72(12)
O(18)-W(6)-O(17)	87.11(11)
O(20)-W(6)-O(17)	153.41(12)
O(19)-W(6)-O(21)	101.64(13)
O(18)-W(6)-O(21)	152.90(12)
O(20)-W(6)-O(21)	86.93(11)
O(17)-W(6)-O(21)	82.34(11)
O(19)-W(6)-O(29)#3	177.09(12)
O(18)-W(6)-O(29)#3	77.68(11)
O(20)-W(6)-O(29)#3	77.59(11)
O(17)-W(6)-O(29)#3	76.19(10)
O(21)-W(6)-O(29)#3	75.58(11)
O(23)-W(7)-O(17)	99.52(13)
O(23)-W(7)-O(25)	105.24(13)
O(17)-W(7)-O(25)	89.83(12)

O(23)-W(7)-O(22)	102.13(12)
O(17)-W(7)-O(22)	88.96(12)
O(25)-W(7)-O(22)	152.42(11)
O(23)-W(7)-O(24)	98.46(13)
O(17)-W(7)-O(24)	161.98(12)
O(25)-W(7)-O(24)	86.68(12)
O(22)-W(7)-O(24)	86.03(12)
O(23)-W(7)-O(29)#3	175.33(12)
O(17)-W(7)-O(29)#3	76.31(10)
O(25)-W(7)-O(29)#3	77.05(10)
O(22)-W(7)-O(29)#3	75.91(10)
O(24)-W(7)-O(29)#3	85.68(10)
O(23)-W(7)-Na(4)#2	64.80(10)
O(17)-W(7)-Na(4)#2	35.48(8)
O(25)-W(7)-Na(4)#2	92.00(9)
O(22)-W(7)-Na(4)#2	102.72(9)
O(24)-W(7)-Na(4)#2	162.24(8)
O(29)#3-W(7)-Na(4)#2	111.30(7)
O(26)-W(8)-O(21)	99.24(13)
O(26)-W(8)-O(25)	106.68(12)
O(21)-W(8)-O(25)	88.47(12)
O(26)-W(8)-O(32)#3	101.25(12)
O(21)-W(8)-O(32)#3	88.42(12)
O(25)-W(8)-O(32)#3	152.04(11)
O(26)-W(8)-O(27)	99.08(13)
O(21)-W(8)-O(27)	161.61(11)
O(25)-W(8)-O(27)	87.75(12)
O(32)#3-W(8)-O(27)	86.52(12)
O(26)-W(8)-O(29)#3	173.74(12)
O(21)-W(8)-O(29)#3	75.38(11)
O(25)-W(8)-O(29)#3	76.68(10)
O(32)#3-W(8)-O(29)#3	75.65(10)
O(27)-W(8)-O(29)#3	86.23(10)
O(28)-W(9)-O(27)	101.41(13)
O(28)-W(9)-O(30)	103.33(12)
O(27)-W(9)-O(30)	90.94(12)

O(28)-W(9)-O(22)#3	102.92(12)
O(27)-W(9)-O(22)#3	90.83(12)
O(30)-W(9)-O(22)#3	152.78(11)
O(28)-W(9)-O(18)#3	94.92(13)
O(27)-W(9)-O(18)#3	163.66(11)
O(30)-W(9)-O(18)#3	85.11(11)
O(22)#3-W(9)-O(18)#3	85.61(11)
O(28)-W(9)-O(29)	169.59(12)
O(27)-W(9)-O(29)	89.00(11)
O(30)-W(9)-O(29)	76.42(10)
O(22)#3-W(9)-O(29)	76.46(10)
O(18)#3-W(9)-O(29)	74.66(10)
O(31)-W(10)-O(24)	101.03(13)
O(31)-W(10)-O(32)	102.27(12)
O(24)-W(10)-O(32)	91.19(12)
O(31)-W(10)-O(30)	103.17(12)
O(24)-W(10)-O(30)	92.45(12)
O(32)-W(10)-O(30)	153.08(11)
O(31)-W(10)-O(20)#3	93.53(12)
O(24)-W(10)-O(20)#3	165.39(11)
O(32)-W(10)-O(20)#3	84.54(11)
O(30)-W(10)-O(20)#3	85.28(11)
O(31)-W(10)-O(29)	168.33(12)
O(24)-W(10)-O(29)	90.63(11)
O(32)-W(10)-O(29)	76.71(10)
O(30)-W(10)-O(29)	76.59(10)
O(20)#3-W(10)-O(29)	74.80(10)
O(31)-W(10)-Na(3)	32.19(10)
O(24)-W(10)-Na(3)	69.24(9)
O(32)-W(10)-Na(3)	107.12(8)
O(30)-W(10)-Na(3)	99.09(8)
O(20)#3-W(10)-Na(3)	125.37(8)
O(29)-W(10)-Na(3)	159.37(7)
O(19)-Na(1)-O(40)	104.24(13)
O(19)-Na(1)-O(37)#4	164.47(13)
O(40)-Na(1)-O(37)#4	91.29(12)

O(19)-Na(1)-O(10)	81.15(11)
O(40)-Na(1)-O(10)	173.21(13)
O(37)#4-Na(1)-O(10)	83.33(11)
O(19)-Na(1)-O(39)	89.17(13)
O(40)-Na(1)-O(39)	96.84(13)
O(37)#4-Na(1)-O(39)	89.34(13)
O(10)-Na(1)-O(39)	87.26(12)
O(19)-Na(1)-O(38)	77.30(12)
O(40)-Na(1)-O(38)	90.46(13)
O(37)#4-Na(1)-O(38)	102.60(12)
O(10)-Na(1)-O(38)	86.69(12)
O(39)-Na(1)-O(38)	165.88(14)
O(19)-Na(1)-Na(3)#4	156.22(10)
O(40)-Na(1)-Na(3)#4	66.88(10)
O(37)#4-Na(1)-Na(3)#4	33.59(8)
O(10)-Na(1)-Na(3)#4	109.73(9)
O(39)-Na(1)-Na(3)#4	70.87(10)
O(38)-Na(1)-Na(3)#4	123.22(10)
O(19)-Na(1)-Na(4)#2	65.63(8)
O(40)-Na(1)-Na(4)#2	129.05(11)
O(37)#4-Na(1)-Na(4)#2	104.29(9)
O(10)-Na(1)-Na(4)#2	49.18(8)
O(39)-Na(1)-Na(4)#2	130.79(10)
O(38)-Na(1)-Na(4)#2	39.12(8)
Na(3)#4-Na(1)-Na(4)#2	137.53(5)
O(34)-Na(2)-O(14)	103.50(14)
O(34)-Na(2)-O(35)	87.76(14)
O(14)-Na(2)-O(35)	168.16(13)
O(34)-Na(2)-O(23)	169.67(15)
O(14)-Na(2)-O(23)	86.35(11)
O(35)-Na(2)-O(23)	82.22(11)
O(34)-Na(2)-O(36)	96.55(13)
O(14)-Na(2)-O(36)	90.74(12)
O(35)-Na(2)-O(36)	84.25(12)
O(23)-Na(2)-O(36)	80.03(11)
O(34)-Na(2)-O(33)	96.27(14)

O(14)-Na(2)-O(33)	85.25(12)
O(35)-Na(2)-O(33)	97.30(13)
O(23)-Na(2)-O(33)	87.51(12)
O(36)-Na(2)-O(33)	167.13(13)
O(34)-Na(2)-Na(3)	110.87(12)
O(14)-Na(2)-Na(3)	126.02(10)
O(35)-Na(2)-Na(3)	44.35(9)
O(23)-Na(2)-Na(3)	59.82(8)
O(36)-Na(2)-Na(3)	46.08(8)
O(33)-Na(2)-Na(3)	128.79(10)
O(34)-Na(2)-Na(4)#2	131.73(11)
O(14)-Na(2)-Na(4)#2	69.36(9)
O(35)-Na(2)-Na(4)#2	105.93(10)
O(23)-Na(2)-Na(4)#2	54.47(8)
O(36)-Na(2)-Na(4)#2	130.18(10)
O(33)-Na(2)-Na(4)#2	37.12(8)
Na(3)-Na(2)-Na(4)#2	110.79(6)
O(34)-Na(2)-H(34A)	15.0(15)
O(14)-Na(2)-H(34A)	93.2(15)
O(35)-Na(2)-H(34A)	98.5(15)
O(23)-Na(2)-H(34A)	172.1(15)
O(36)-Na(2)-H(34A)	107.9(15)
O(33)-Na(2)-H(34A)	84.6(15)
Na(3)-Na(2)-H(34A)	125.8(15)
Na(4)#2-Na(2)-H(34A)	118.1(15)
O(35)-Na(3)-O(37)	96.60(12)
O(35)-Na(3)-O(36)	88.48(13)
O(37)-Na(3)-O(36)	88.77(12)
O(35)-Na(3)-O(31)	172.12(13)
O(37)-Na(3)-O(31)	88.65(12)
O(36)-Na(3)-O(31)	97.53(12)
O(35)-Na(3)-O(16)#5	89.42(12)
O(37)-Na(3)-O(16)#5	127.48(13)
O(36)-Na(3)-O(16)#5	143.67(13)
O(31)-Na(3)-O(16)#5	82.71(11)
O(35)-Na(3)-O(23)	73.58(11)

O(37)-Na(3)-O(23)	158.39(12)
O(36)-Na(3)-O(23)	72.07(11)
O(31)-Na(3)-O(23)	103.36(11)
O(16)#5-Na(3)-O(23)	72.58(10)
O(35)-Na(3)-Na(2)	46.64(9)
O(37)-Na(3)-Na(2)	113.41(10)
O(36)-Na(3)-Na(2)	48.52(9)
O(31)-Na(3)-Na(2)	135.89(10)
O(16)#5-Na(3)-Na(2)	108.27(10)
O(23)-Na(3)-Na(2)	46.02(7)
O(35)-Na(3)-W(10)	151.80(10)
O(37)-Na(3)-W(10)	111.36(9)
O(36)-Na(3)-W(10)	95.47(9)
O(31)-Na(3)-W(10)	22.81(7)
O(16)#5-Na(3)-W(10)	71.01(8)
O(23)-Na(3)-W(10)	81.10(7)
Na(2)-Na(3)-W(10)	119.63(6)
O(35)-Na(3)-Na(1)#7	95.72(10)
O(37)-Na(3)-Na(1)#7	34.06(8)
O(36)-Na(3)-Na(1)#7	122.82(10)
O(31)-Na(3)-Na(1)#7	85.33(9)
O(16)#5-Na(3)-Na(1)#7	93.48(9)
O(23)-Na(3)-Na(1)#7	162.15(8)
Na(2)-Na(3)-Na(1)#7	134.23(6)
W(10)-Na(3)-Na(1)#7	105.29(5)
O(28)-Na(4)-O(1)#6	95.40(12)
O(28)-Na(4)-O(17)#5	154.95(13)
O(1)#6-Na(4)-O(17)#5	87.92(11)
O(28)-Na(4)-O(12)#5	96.80(12)
O(1)#6-Na(4)-O(12)#5	158.42(13)
O(17)#5-Na(4)-O(12)#5	88.51(11)
O(28)-Na(4)-O(33)#5	82.55(11)
O(1)#6-Na(4)-O(33)#5	79.92(12)
O(17)#5-Na(4)-O(33)#5	122.42(12)
O(12)#5-Na(4)-O(33)#5	84.09(11)
O(28)-Na(4)-O(38)#5	74.79(11)

O(1)#6-Na(4)-O(38)#5	75.54(11)
O(17)#5-Na(4)-O(38)#5	82.09(11)
O(12)#5-Na(4)-O(38)#5	124.95(12)
O(33)#5-Na(4)-O(38)#5	144.54(12)
O(28)-Na(4)-W(3)#5	76.48(9)
O(1)#6-Na(4)-W(3)#5	170.50(10)
O(17)#5-Na(4)-W(3)#5	97.37(8)
O(12)#5-Na(4)-W(3)#5	30.46(7)
O(33)#5-Na(4)-W(3)#5	103.57(9)
O(38)#5-Na(4)-W(3)#5	97.29(9)
O(28)-Na(4)-W(7)#5	167.67(10)
O(1)#6-Na(4)-W(7)#5	72.37(8)
O(17)#5-Na(4)-W(7)#5	27.65(7)
O(12)#5-Na(4)-W(7)#5	95.31(8)
O(33)#5-Na(4)-W(7)#5	96.47(9)
O(38)#5-Na(4)-W(7)#5	99.99(9)
W(3)#5-Na(4)-W(7)#5	115.55(4)
O(28)-Na(4)-Na(2)#5	117.56(9)
O(1)#6-Na(4)-Na(2)#5	80.15(9)
O(17)#5-Na(4)-Na(2)#5	87.47(9)
O(12)#5-Na(4)-Na(2)#5	78.44(8)
O(33)#5-Na(4)-Na(2)#5	35.11(8)
O(38)#5-Na(4)-Na(2)#5	153.80(10)
W(3)#5-Na(4)-Na(2)#5	107.87(5)
W(7)#5-Na(4)-Na(2)#5	62.87(4)
O(28)-Na(4)-Na(1)#5	73.09(9)
O(1)#6-Na(4)-Na(1)#5	111.35(9)
O(17)#5-Na(4)-Na(1)#5	82.57(9)
O(12)#5-Na(4)-Na(1)#5	89.26(9)
O(33)#5-Na(4)-Na(1)#5	153.79(10)
O(38)#5-Na(4)-Na(1)#5	35.82(8)
W(3)#5-Na(4)-Na(1)#5	61.86(4)
W(7)#5-Na(4)-Na(1)#5	109.40(5)
Na(2)#5-Na(4)-Na(1)#5	164.37(5)
W(1)-O(1)-Na(4)#8	154.91(18)
W(2)-O(2)-W(1)	117.05(14)

W(5)#1-O(3)-W(3)#1	92.18(9)
W(5)#1-O(3)-W(2)	91.64(10)
W(3)#1-O(3)-W(2)	176.12(14)
W(5)#1-O(3)-W(1)	91.72(10)
W(3)#1-O(3)-W(1)	90.81(9)
W(2)-O(3)-W(1)	89.75(9)
W(5)#1-O(3)-W(4)#1	91.76(9)
W(3)#1-O(3)-W(4)#1	89.24(9)
W(2)-O(3)-W(4)#1	89.96(9)
W(1)-O(3)-W(4)#1	176.51(13)
W(1)-O(4)-W(4)	172.89(16)
W(2)-O(5)-W(5)#1	115.73(14)
W(2)-O(7)-W(4)#1	117.02(13)
W(3)-O(8)-W(2)	171.04(16)
W(4)-O(9)-W(3)	116.89(13)
W(3)-O(10)-Na(1)	157.85(16)
W(1)#1-O(11)-W(3)	117.19(14)
W(5)-O(12)-W(3)	115.60(14)
W(5)-O(12)-Na(4)#2	131.59(15)
W(3)-O(12)-Na(4)#2	112.03(13)
W(5)-O(13)-W(1)#1	115.47(14)
W(5)-O(14)-Na(2)	144.41(16)
W(4)-O(15)-W(5)	116.65(14)
W(4)-O(16)-Na(3)#2	152.81(16)
W(7)-O(17)-W(6)	116.09(13)
W(7)-O(17)-Na(4)#2	116.87(13)
W(6)-O(17)-Na(4)#2	126.52(14)
W(6)-O(18)-W(9)#3	116.09(14)
W(6)-O(19)-Na(1)	151.37(16)
W(6)-O(20)-W(10)#3	115.49(14)
W(8)-O(21)-W(6)	116.99(14)
W(9)#3-O(22)-W(7)	117.26(13)
W(7)-O(23)-Na(2)	154.62(16)
W(7)-O(23)-Na(3)	117.62(14)
Na(2)-O(23)-Na(3)	74.16(9)
W(10)-O(24)-W(7)	172.91(17)

W(7)-O(25)-W(8)	117.37(14)
W(9)-O(27)-W(8)	171.79(16)
W(9)-O(28)-Na(4)	148.39(17)
W(6)#3-O(29)-W(10)	92.00(10)
W(6)#3-O(29)-W(9)	91.50(10)
W(10)-O(29)-W(9)	90.24(9)
W(6)#3-O(29)-W(7)#3	91.36(9)
W(10)-O(29)-W(7)#3	176.57(14)
W(9)-O(29)-W(7)#3	90.37(9)
W(6)#3-O(29)-W(8)#3	91.96(9)
W(10)-O(29)-W(8)#3	90.44(9)
W(9)-O(29)-W(8)#3	176.45(14)
W(7)#3-O(29)-W(8)#3	88.75(9)
W(9)-O(30)-W(10)	116.17(13)
W(10)-O(31)-Na(3)	125.00(16)
W(10)-O(32)-W(8)#3	117.12(13)
Na(2)-O(33)-Na(4)#2	107.77(13)
Na(2)-O(33)-H(33A)	105(4)
Na(4)#2-O(33)-H(33A)	118(4)
Na(2)-O(33)-H(33B)	131(4)
Na(4)#2-O(33)-H(33B)	108(4)
H(33A)-O(33)-H(33B)	87(5)
Na(2)-O(34)-H(34A)	111(5)
Na(2)-O(34)-H(34B)	121(4)
H(34A)-O(34)-H(34B)	107(6)
Na(3)-O(35)-Na(2)	89.02(12)
Na(3)-O(35)-H(35A)	134(4)
Na(2)-O(35)-H(35A)	104(4)
Na(3)-O(35)-H(35B)	123(3)
Na(2)-O(35)-H(35B)	110(3)
H(35A)-O(35)-H(35B)	94(5)
Na(3)-O(36)-Na(2)	85.40(11)
Na(3)-O(36)-H(36A)	127(3)
Na(2)-O(36)-H(36A)	106(3)
Na(3)-O(36)-H(36B)	113(4)
Na(2)-O(36)-H(36B)	121(4)

H(36A)-O(36)-H(36B)	105(5)
Na(3)-O(37)-Na(1)#7	112.35(14)
Na(3)-O(37)-H(37A)	110(3)
Na(1)#7-O(37)-H(37A)	103(4)
Na(3)-O(37)-H(37B)	118(3)
Na(1)#7-O(37)-H(37B)	99(3)
H(37A)-O(37)-H(37B)	113(5)
Na(1)-O(38)-Na(4)#2	105.06(13)
Na(1)-O(38)-H(38A)	130(4)
Na(4)#2-O(38)-H(38A)	92(4)
Na(1)-O(38)-H(38B)	112(4)
Na(4)#2-O(38)-H(38B)	113(4)
H(38A)-O(38)-H(38B)	102(6)
Na(1)-O(39)-H(39A)	119(5)
Na(1)-O(39)-H(39B)	120(5)
H(39A)-O(39)-H(39B)	100(7)
Na(1)-O(40)-H(40A)	122(4)
Na(1)-O(40)-H(40B)	109(4)
H(40A)-O(40)-H(40B)	102(6)
H(41A)-O(41)-H(41B)	98(5)
H(42A)-O(42)-H(42B)	94(6)
H(43A)-O(43)-H(43B)	102(6)

Symmetry transformations used to generate equivalent atoms:

#1 -x+2,-y+1,-z+2 #2 x+1/2,-y+1/2,z+1/2 #3 -x+1,-y+1,-z+1

#4 x-1/2,-y+1/2,z+1/2 #5 x-1/2,-y+1/2,z-1/2

#6 x-1,y,z-1 #7 x+1/2,-y+1/2,z-1/2 #8 x+1,y,z+1

Supplementary Table 11. Anisotropic displacement parameters ($Å^2 \times 10^3$) for NaDT. The anisotropic displacement factor exponent takes the form of $-2p^2 [h^2 a^{*2}U^{11} + ... + 2 h k a^* b^* U^{12}]$

	U^{11}	U^{22}	U^{33}	U^{23}	U^{13}	U^{12}
W(1)	6(1)	5(1)	6(1)	1(1)	1(1)	1(1)
W(2)	6(1)	6(1)	7(1)	0(1)	2(1)	-1(1)
W(3)	5(1)	5(1)	6(1)	0(1)	1(1)	0(1)
W(4)	5(1)	6(1)	7(1)	0(1)	2(1)	1(1)
W(5)	9(1)	7(1)	5(1)	-1(1)	3(1)	-1(1)
W(6)	7(1)	6(1)	4(1)	1(1)	2(1)	1(1)
W(7)	5(1)	6(1)	5(1)	0(1)	1(1)	0(1)
W(8)	5(1)	5(1)	6(1)	0(1)	1(1)	0(1)
W(9)	6(1)	6(1)	6(1)	0(1)	2(1)	-1(1)
W(10)	5(1)	6(1)	5(1)	1(1)	2(1)	1(1)
Na(1)	9(1)	12(1)	11(1)	2(1)	3(1)	-1(1)
Na(2)	12(1)	13(1)	10(1)	-2(1)	4(1)	1(1)
Na(3)	10(1)	12(1)	15(1)	4(1)	1(1)	-1(1)
Na(4)	17(1)	7(1)	9(1)	0(1)	-3(1)	0(1)
O(1)	14(2)	11(1)	10(2)	1(1)	1(1)	-1(1)
O(2)	9(1)	8(1)	9(2)	2(1)	2(1)	1(1)
O(3)	7(1)	6(1)	4(1)	-1(1)	1(1)	-1(1)
O(4)	8(1)	8(1)	8(2)	0(1)	2(1)	0(1)
O(5)	10(1)	9(1)	7(2)	0(1)	4(1)	0(1)
O(6)	10(1)	14(1)	15(2)	-2(1)	6(1)	-3(1)
O(7)	6(1)	7(1)	6(1)	1(1)	1(1)	0(1)
O(8)	8(1)	8(1)	8(2)	2(1)	1(1)	1(1)
O(9)	6(1)	7(1)	6(2)	2(1)	2(1)	2(1)
O(10)	10(1)	9(1)	10(2)	1(1)	3(1)	-1(1)
O(11)	9(1)	7(1)	9(2)	-1(1)	2(1)	-1(1)
O(12)	9(1)	7(1)	6(1)	-1(1)	2(1)	-1(1)
O(13)	13(1)	8(1)	6(2)	1(1)	6(1)	0(1)
O(14)	16(2)	11(1)	7(2)	0(1)	3(1)	-1(1)
O(15)	10(1)	9(1)	6(2)	-1(1)	3(1)	1(1)
O(16)	10(1)	12(1)	8(2)	0(1)	3(1)	2(1)

O(17)	6(1)	9(1)	7(2)	2(1)	0(1)	1(1)
O(18)	9(1)	6(1)	7(2)	0(1)	2(1)	1(1)
O(19)	12(1)	12(1)	6(2)	2(1)	5(1)	0(1)
O(20)	9(1)	8(1)	5(1)	2(1)	3(1)	0(1)
O(21)	7(1)	8(1)	8(2)	1(1)	3(1)	-1(1)
O(22)	7(1)	6(1)	7(1)	-1(1)	0(1)	0(1)
O(23)	7(1)	13(1)	8(2)	1(1)	2(1)	1(1)
O(24)	8(1)	7(1)	10(2)	-1(1)	2(1)	-1(1)
O(25)	6(1)	9(1)	8(2)	-1(1)	2(1)	0(1)
O(26)	9(1)	10(1)	10(2)	1(1)	2(1)	0(1)
O(27)	8(1)	9(1)	5(1)	-1(1)	3(1)	0(1)
O(28)	14(2)	9(1)	10(2)	-2(1)	4(1)	0(1)
O(29)	7(1)	7(1)	6(1)	1(1)	3(1)	0(1)
O(30)	9(1)	4(1)	6(1)	1(1)	3(1)	1(1)
O(31)	11(1)	10(1)	8(2)	0(1)	3(1)	-1(1)
O(32)	6(1)	7(1)	9(2)	1(1)	3(1)	0(1)
O(33)	18(2)	14(2)	16(2)	2(1)	4(1)	-1(1)
O(34)	13(2)	39(2)	15(2)	9(2)	6(1)	-3(2)
O(35)	10(2)	13(1)	16(2)	1(1)	6(1)	2(1)
O(36)	10(2)	13(1)	18(2)	-4(1)	9(1)	-3(1)
O(37)	11(1)	12(1)	12(2)	0(1)	2(1)	2(1)
O(38)	19(2)	11(1)	14(2)	0(1)	3(1)	3(1)
O(39)	13(2)	27(2)	24(2)	-3(2)	8(1)	1(2)
O(40)	15(2)	12(2)	21(2)	1(1)	8(1)	1(1)
O(41)	16(2)	14(2)	14(2)	1(1)	6(1)	3(1)
O(42)	13(2)	12(2)	33(2)	-5(1)	5(2)	2(1)
O(43)	9(2)	14(2)	18(2)	1(1)	4(1)	-1(1)

Supplementary Table 12. Hydrogen coordinates (×10^4) and isotropic displacement parameters ($Å^2$×10^3) for NaDT.

	x	y	z	U(eq)
H(33A)	9700(50)	1650(30)	7280(30)	24
H(33B)	10070(50)	1980(30)	7780(20)	24
H(34A)	12450(50)	2600(40)	7200(30)	33
H(34B)	12710(50)	2910(40)	6700(30)	33
H(35A)	9560(50)	1600(30)	5870(30)	18
H(35B)	10580(50)	1720(30)	5730(30)	18
H(36A)	10080(50)	4520(30)	5820(30)	19
H(36B)	10890(50)	4090(30)	5610(30)	19
H(37A)	10570(50)	3440(30)	4200(20)	18
H(37B)	11240(40)	2750(30)	4590(20)	18
H(38A)	5530(50)	1690(40)	7560(30)	22
H(38B)	4890(50)	2240(40)	7260(30)	22
H(39A)	4200(40)	4100(40)	9390(30)	31
H(39B)	5200(50)	4150(40)	9590(20)	31
H(40A)	2480(40)	2750(30)	8360(30)	23
H(40B)	2960(50)	2080(20)	8360(30)	23
H(41A)	2380(50)	5490(30)	7080(30)	22
H(41B)	2100(50)	5730(30)	7590(30)	22
H(42A)	6030(60)	5450(40)	8630(30)	30
H(42B)	5030(50)	5310(40)	8640(30)	30
H(43A)	9120(50)	5450(30)	6390(30)	21
H(43B)	10210(50)	5670(30)	6530(30)	21

Supplementary Table 13. Torsion angles [°] for NaDT.

O(4)-W(1)-O(1)-Na(4)#8	-154.9(4)
O(2)-W(1)-O(1)-Na(4)#8	-59.9(4)
O(11)#1-W(1)-O(1)-Na(4)#8	111.2(4)
O(13)#1-W(1)-O(1)-Na(4)#8	25.1(4)
O(3)-W(1)-O(1)-Na(4)#8	29.8(9)
O(8)-W(3)-O(10)-Na(1)	72.5(5)
O(9)-W(3)-O(10)-Na(1)	167.3(4)
O(11)-W(3)-O(10)-Na(1)	-19.6(5)
O(12)-W(3)-O(10)-Na(1)	-105.8(5)
O(3)#1-W(3)-O(10)-Na(1)	-96.8(7)
Na(4)#2-W(3)-O(10)-Na(1)	-115.1(5)
O(14)-W(5)-O(12)-W(3)	-179.70(14)
O(13)-W(5)-O(12)-W(3)	-75.13(15)
O(5)#1-W(5)-O(12)-W(3)	12.6(3)
O(15)-W(5)-O(12)-W(3)	79.00(15)
O(3)#1-W(5)-O(12)-W(3)	2.47(12)
O(14)-W(5)-O(12)-Na(4)#2	11.4(2)
O(13)-W(5)-O(12)-Na(4)#2	115.98(19)
O(5)#1-W(5)-O(12)-Na(4)#2	-156.24(19)
O(15)-W(5)-O(12)-Na(4)#2	-89.89(19)
O(3)#1-W(5)-O(12)-Na(4)#2	-166.4(2)
O(14)-W(5)-O(13)-W(1)#1	179.07(14)
O(12)-W(5)-O(13)-W(1)#1	74.42(15)
O(5)#1-W(5)-O(13)-W(1)#1	-78.85(15)
O(15)-W(5)-O(13)-W(1)#1	-10.1(4)
O(3)#1-W(5)-O(13)-W(1)#1	-2.28(13)
O(13)-W(5)-O(14)-Na(2)	-146.4(3)
O(12)-W(5)-O(14)-Na(2)	-51.0(3)
O(5)#1-W(5)-O(14)-Na(2)	123.3(3)
O(15)-W(5)-O(14)-Na(2)	37.7(3)
O(9)-W(4)-O(16)-Na(3)#2	170.9(3)
O(15)-W(4)-O(16)-Na(3)#2	79.2(4)
O(7)#1-W(4)-O(16)-Na(3)#2	-10.7(4)
O(4)-W(4)-O(16)-Na(3)#2	-99.3(4)

O(23)-W(7)-O(17)-W(6)	175.99(15)
O(25)-W(7)-O(17)-W(6)	-78.58(15)
O(22)-W(7)-O(17)-W(6)	73.87(15)
O(24)-W(7)-O(17)-W(6)	0.1(5)
O(29)#3-W(7)-O(17)-W(6)	-1.88(12)
Na(4)#2-W(7)-O(17)-W(6)	-172.3(2)
O(23)-W(7)-O(17)-Na(4)#2	-11.75(17)
O(25)-W(7)-O(17)-Na(4)#2	93.68(15)
O(22)-W(7)-O(17)-Na(4)#2	-113.88(15)
O(24)-W(7)-O(17)-Na(4)#2	172.4(3)
O(29)#3-W(7)-O(17)-Na(4)#2	170.38(16)
O(19)-W(6)-O(18)-W(9)#3	-178.81(14)
O(20)-W(6)-O(18)-W(9)#3	-74.62(15)
O(17)-W(6)-O(18)-W(9)#3	78.80(15)
O(21)-W(6)-O(18)-W(9)#3	11.9(3)
O(29)#3-W(6)-O(18)-W(9)#3	2.30(12)
O(18)-W(6)-O(19)-Na(1)	-143.5(3)
O(20)-W(6)-O(19)-Na(1)	121.1(3)
O(17)-W(6)-O(19)-Na(1)	-53.1(4)
O(21)-W(6)-O(19)-Na(1)	31.6(4)
O(19)-W(6)-O(20)-W(10)#3	179.82(14)
O(18)-W(6)-O(20)-W(10)#3	73.97(15)
O(17)-W(6)-O(20)-W(10)#3	-12.8(3)
O(21)-W(6)-O(20)-W(10)#3	-78.94(15)
O(29)#3-W(6)-O(20)-W(10)#3	-3.04(12)
O(17)-W(7)-O(23)-Na(2)	-74.1(4)
O(25)-W(7)-O(23)-Na(2)	-166.6(4)
O(22)-W(7)-O(23)-Na(2)	16.8(4)
O(24)-W(7)-O(23)-Na(2)	104.6(4)
Na(4)#2-W(7)-O(23)-Na(2)	-81.6(4)
O(17)-W(7)-O(23)-Na(3)	173.26(13)
O(25)-W(7)-O(23)-Na(3)	80.82(15)
O(22)-W(7)-O(23)-Na(3)	-95.75(15)
O(24)-W(7)-O(23)-Na(3)	-8.02(15)
Na(4)#2-W(7)-O(23)-Na(3)	165.75(16)
O(27)-W(9)-O(28)-Na(4)	-137.6(3)

O(30)-W(9)-O(28)-Na(4)	-43.9(3)
O(22)#3-W(9)-O(28)-Na(4)	128.8(3)
O(18)#3-W(9)-O(28)-Na(4)	42.2(3)
O(29)-W(9)-O(28)-Na(4)	43.5(8)
O(24)-W(10)-O(31)-Na(3)	9.04(19)
O(32)-W(10)-O(31)-Na(3)	102.70(17)
O(30)-W(10)-O(31)-Na(3)	-86.15(18)
O(20)#3-W(10)-O(31)-Na(3)	-172.12(17)
O(29)-W(10)-O(31)-Na(3)	-173.6(4)

Symmetry transformations used to generate equivalent atoms:

#1 -x+2,-y+1,-z+2 #2 x+1/2,-y+1/2,z+1/2 #3 -x+1,-y+1,-z+1

#4 x-1/2,-y+1/2,z+1/2 #5 x-1/2,-y+1/2,z-1/2

#6 x-1,y,z-1 #7 x+1/2,-y+1/2,z-1/2 #8 x+1,y,z+1

Supplementary Table 14. Hydrogen bonds for NaDT [Å and °].

D-H...A	d(D-H)	d(H...A)	d(D...A)	<(DHA)
O(33)-H(33A)...O(42)#9	0.84(4)	2.22(4)	2.984(5)	152(5)
O(33)-H(33B)...O(30)#2	0.85(4)	2.47(4)	3.259(4)	156(5)
O(34)-H(34B)...O(21)#10	0.83(6)	2.02(6)	2.848(4)	171(6)
O(35)-H(35A)...O(42)#9	0.82(6)	1.96(6)	2.730(5)	155(5)
O(35)-H(35B)...O(6)#7	0.93(5)	1.85(5)	2.772(4)	171(5)
O(36)-H(36A)...O(43)	0.90(5)	1.90(6)	2.792(5)	171(5)
O(36)-H(36B)...O(32)#11	0.75(5)	2.19(6)	2.935(4)	174(6)
O(37)-H(37A)...O(43)#11	0.87(4)	1.87(4)	2.726(4)	167(5)
O(37)-H(37B)...O(26)#10	0.88(4)	2.06(4)	2.900(4)	160(5)
O(38)-H(38A)...O(13)#9	0.80(6)	2.35(6)	3.107(4)	158(5)
O(38)-H(38B)...O(21)	0.76(6)	2.56(6)	3.173(4)	139(5)
O(39)-H(39A)...O(7)#12	0.72(4)	2.24(4)	2.945(4)	166(7)
O(39)-H(39B)...O(6)	0.73(4)	2.55(4)	3.268(5)	167(6)
O(40)-H(40A)...O(15)#13	0.78(4)	2.13(4)	2.898(4)	172(6)
O(40)-H(40B)...O(31)#4	0.77(4)	2.45(4)	3.167(4)	155(6)
O(40)-H(40B)...O(41)#14	0.77(4)	2.34(5)	2.880(4)	128(5)
O(41)-H(41A)...O(20)	0.82(6)	2.01(6)	2.801(4)	163(5)
O(41)-H(41B)...O(5)#12	0.84(6)	2.20(6)	2.910(4)	142(5)
O(42)-H(42A)...O(11)	0.75(6)	2.06(6)	2.778(4)	161(6)
O(42)-H(42B)...O(39)	0.85(6)	2.41(6)	3.203(5)	156(5)
O(43)-H(43A)...O(22)	0.83(6)	2.09(6)	2.818(4)	147(5)
O(43)-H(43B)...O(41)#10	0.78(6)	2.05(6)	2.792(5)	160(6)

Symmetry transformations used to generate equivalent atoms:

#1 -x+2,-y+1,-z+2 #2 x+1/2,-y+1/2,z+1/2 #3 -x+1,-y+1,-z+1

#4 x-1/2,-y+1/2,z+1/2 #5 x-1/2,-y+1/2,z-1/2

#6 x-1,y,z-1 #7 x+1/2,-y+1/2,z-1/2 #8 x+1,y,z+1

#9 -x+3/2,y-1/2,-z+3/2 #10 x+1,y,z #11 -x+2,-y+1,-z+1

#12 -x+1,-y+1,-z+2 #13 x-1,y,z #14 -x+1/2,y-1/2,-z+3/2

Supplementary References

1. Le Manach, C. et al. Medicinal chemistry optimization of antiplasmodial imidazopyridazine hits from high throughput screening of a softfocus kinase library: Part 2. *J. Med. Chem.* **57**, 8839-8848 (2014).
2. Campbell, M. W., Compton, J. S., Kelly, C. B. & Molander, G. A. Three-component olefin dicarbofunctionalization enabled by nickel/photoredox dual catalysis. *J. Am. Chem. Soc.* **141**, 20069-20078 (2019).
3. Proctor, R. S., Davis, H. J. & Phipps, R. J. Catalytic enantioselective Minisci-type addition to heteroarenes. *Science* **360**, 419-422 (2018).
4. Zhang, G. et al. External oxidant-free oxidative cross-coupling: a photoredox cobalt-catalyzed aromatic C–H thiolation for constructing C–S bonds. *J. Am. Chem. Soc.* **137**, 9273-9280 (2015).
5. Zhao, J. et al. Intramolecular crossed [2+ 2] photocycloaddition through visible light-induced energy transfer. *J. Am. Chem. Soc.* **139**, 9807-9810 (2017).
6. Corcoran, E. B. et al. Aryl amination using ligand-free Ni (II) salts and photoredox catalysis. *Science* **353**, 279-283 (2016).
7. Ma, J. et al. Photochemical intermolecular dearomative cycloaddition of bicyclic azaarenes with alkenes. *Science* **371**, 1338-1345 (2021).
8. Jin, J. & MacMillan, D. W. Alcohols as alkylating agents in heteroarene C–H functionalization. *Nature* **525**, 87-90 (2015).
9. Jin, X. et al. Effects of thermal cross-linking on the structure and property of asymmetric membrane prepared from the polyacrylonitrile. *Polymers* **10**, 539 (2018).
10. Standage, A. & Matkowsky, R. Thermal oxidation of polyacrylonitrile. *Eur. Polym. J.* **7**, 775-783 (1971).
11. Liang, Y. Z. et al. Graphene quantum dots (GQDs)-polyethyleneimine as interlayer for the fabrication of high performance organic solvent nanofiltration (OSN) membranes. *Chem. Eng. J.* **380**, 16 (2020).
12. Li, S. X. et al. Amino-functionalized graphene quantum dots (aGQDs)-embedded thin film nanocomposites for solvent resistant nanofiltration (SRNF) membranes based on covalence interactions. *J. Membr. Sci.* **588**, 13 (2019).
13. Guo, Y., Li, S. X., Su, B. W. & Mandal, B. Fluorine incorporation for enhancing solvent resistance of organic solvent nanofiltration membrane. *Chem. Eng. J.* **369**, 498-510 (2019).
14. Li, Y. Y. et al. Graphene oxide (GO)-interlayered thin-film nanocomposite (TFN) membranes with high solvent resistance for organic solvent nanofiltration (OSN). *J. Mater. Chem. A* **7**, 13315-13330 (2019).
15. Li, S. X. et al. Graphene Quantum Dots-Doped Thin Film Nanocomposite Polyimide Membranes with Enhanced Solvent Resistance for Solvent-Resistant Nanofiltration. *ACS Appl. Mater. Interfaces* **11**, 6527-6540 (2019).
16. Abdellah, M. H. et al. A catechin/cellulose composite membrane for organic solvent nanofiltration. *J. Membr. Sci.* **567**, 139-145 (2018).
17. Mertens, M., Van Goethem, C., Thijs, M., Koeckelberghs, G. & Vankelecom, I. F. J. Crosslinked PVDF-membranes for solvent resistant nanofiltration. *J. Membr. Sci.* **566**, 223-230 (2018).

18. Gao, T. T., Wu, H. B., Tao, L., Qu, L. T. & Li, C. Enhanced stability and separation efficiency of graphene oxide membranes in organic solvent nanofiltration. *J. Mater. Chem. A* **6**, 19563-19569 (2018).
19. Li, C., Li, S. X., Lv, L., Su, B. W. & Hu, M. Z. High solvent-resistant and integrally crosslinked polyimide-based composite membranes for organic solvent nanofiltration. *J. Membr. Sci.* **564**, 10-21 (2018).
20. Ilyas, S. et al. Weak polyelectrolyte multilayers as tunable membranes for solvent resistant nanofiltration. *J. Membr. Sci.* **514**, 322-331 (2016).
21. Yuan, S. S. et al. New promising polymer for organic solvent nanofiltration: Oxidized poly(arylene sulfide sulfone). *J. Membr. Sci.* **549**, 438-445 (2018).
22. Aburabie, J. & Peinemann, K. V. Crosslinked poly(ether block amide) composite membranes for organic solvent nanofiltration applications. *J. Membr. Sci.* **523**, 264-272 (2017).
23. Lu, T. & Chen, F. Multiwfn: a multifunctional wavefunction analyzer. *J. Comput. Chem.* **33**, 580-592 (2012).
